# Supplementary material for: Cross‐species complementation reveals conserved functions for EARLY FLOWERING 3 between monocots and dicots
Source: Plant Direct. 2017 Oct 16;1(4):e00018. doi: 10.1002/pld3.18 (PMC6508535; doi:10.1002/pld3.18)
Supplement: Supplementary file 1 [file PLD3-1-e00018-s001.pdf]

Supplemental Figure S1. Multiple sequence alignments of ELF3 orthologs **(A)** and percentage of identical amino acid sequences **(B)**. Protein sequences of AtELF3 (AT2G25930.1), BdELF3 (Bradi2g14290.1), SvELF3a (Sevir.5G206400.1) and SvELF3b (Sevir.3G123200.1) were used for multiple sequence alignments and for generating percentage of identical amino acids by Clustal Omega alignment with default parameters.

Supplemental Figure S2. Diel and circadian expression of *BdELF3* from the DIURNAL database. GCRMA (GeneChip Robust Multiarray Averaging) values from the DIURNAL database (Mockler et al., 2007) were plotted to show time-course expression profiles of *Bradi2g14290 (BdELF3)* under either diel **(A)** or circadian conditions **(B)**. Diel expression of *AtELF3* from DIURNAL database was used for comparison in **(A)**. Shade boxes indicate dark periods. In **(B)**, Circadian expression data was obtained by entraining plants with either photo- (LDHH) or thermo- (LLHC) conditions followed by sampling under the Free-Running condition (F) with constant light and temperature.

Supplemental Figure S3. Example of the Diel Explorer interface. The search interface (left) and plotting interface of Diel Explorer are shown (right). Users can search by gene or ortholog id, or by gene ontology term. Alternately, users can filter data by period, phase (lag) or significance cut offs. Data can be plotted in a line graph or heatmap.

Supplemental Figure S4. Summary of circadian regulated genes in *S. viridis*. Distribution of circadian regulated genes in *S. viridis* was plotted by their phases, with the y axis showing the number of genes considered significantly (Bonferroni Adjusted P-Value < 0.001) cycling under photo- (LDHH) or thermo- (LLHC) entrainment in *S. viridis* followed by free-running condition (F).

Supplemental Figure S5. Circadian expression of selected *A. thaliana* clock genes from the DIURNAL database. GCRMA (GeneChip Robust Multiarray Averaging) values were plotted to show time-course expression profiles of selected *A. thaliana* clock genes under

either photo- (LL23_LDHH) or thermo-entrainment (LL_LLHC) from the DIURNAL database (Mockler et al., 2007). Each gene cycles with a correlation of > 0.9 when compared to a best fit model (24-hour rhythm).

Supplemental Figure S6. Anti-FLAG western of ELF3 transgenic lines used for complementation analysis. Representative blot of protein extracts from day 12 seedlings taken at Zeitgeber time 12 grown under 12-hour light :12-hour dark growth conditions at 22 ˚C that were probed with FLAG antibody to detect the 3xFLAG epitope. RPT5 is used as a loading control.

Supplemental Figure S7. Relative Amplitude Error vs period plots. The periods and relative amplitude error (RAE) of 8 AtELF3 *elf3-2* (**A**), BdELF3 *elf3-2* (**B**), and SvELF3 *elf3-2* (**C**) seedlings were plotted along with wild type and *elf3-2* mutants (Note, only 3 of 8 *elf3* seedlings has measurable rhythms). RAE=0.5 was used as a cutoff (dotted line), above which a seedling is not considered rhythmic (Plautz et al., 1997). Note that wild type and *elf3* mutant data were reproduced on all plots for comparison purposes.

Supplemental Figure 1

A

```
AtELF3     1 MKR---------GKDEEKILEPMFPRLHVNDADK-GGPRAPPRNKMALYEQLSIPSQRFGD
BdELF3     1 MRR-GGGPAPAGKEDKVMGPLFPRLHVNDTLK-GGPRAPPRNKMALYEQFSVPSQRFTP
SvELF3a    1 MTRGGAAGGGGREEQGKVMGPLFPRLHVSDAGKGGPRAPPRNKMALYEQFTVPSNRFSS
SvELF3b    1 MRR-GAGK---DEAPDKVMGPLFPRLHVNDTVK-GGPRAPPRNKMALYEQFSVPSHRFSA

AtELF3    52 HGTMNSRSNNT-------------STLVHPGPSSQPCGVERNLSV-------------QH
BdELF3    59 HRAS--------------------SSALSSASPGGQIGGSDRPLFPSFCVPSNEPARSSEH
SvELF3a   61 SAASTRA---------------AGGSLVISTSASQVYSYDRPLFQPFDVPSNGPAHSSEK
SvELF3b   56 AAAAPAAPAPAPPWHAHRPAPGAATSAVPSTSASQAGGSDRPLFPSFCVPSTEPVRSSDH

AtELF3    86 LDSSAANQ---AT----EKFVSQM-------------SFMENVRSSAQHDQRKMVREEE
BdELF3    99 INTNSNGRDGNATRVESGRHSTQLKSKDTYAAGSTAECSSQRRE-NSVKNSSGKKLTNDD
SvELF3a  106 FKGNSINGQSNSTRRESGRMPTQTKNNDVYASKSIAESTSQHRVGNINKNSSGKKVANND
SvELF3b  116 MNANSNGRAGNATRAESGRISTHLKSKDTNAAGPTAECSSKHRE-NTTKNSSGKKLTNDD

AtELF3   125 DFAVPVYINSRRSQSHGRTKSGIEKEKHTPMVAPSSHHSIRFQEVNQTGSKQNVCLATCS
BdELF3   158 DFTVPSVFCSGVPPHSTQEVVRIQ-EKSTAFPSTSPYKSGP---------TMSKSSAKCS
SvELF3a  166 EFMVPSICSPRFSRYSTQEHAGVQ-DKSNPLSATNPHKSPS---------AMPKSSAECY
SvELF3b  175 DFTVPSVLYSGIPPHSTQE-------KFTPFPTKSPYKSMP---------AMSKSSAKCS

AtELF3   185 KPEVRDQVKA---NARS---GGFVISLDVSVTEEIDLE-------------------KSAS
BdELF3   208 NTDKRYLEGTNVSDMRSRDSPSIKDKAPLKTMTNLDVEERSSSFQISKEKAGKADDKISS
SvELF3a  216 SAVNRHLERIDESDMRSMSSSKIKEKESVQGSKIVEVEEKSLPVQAFKEKFKNKDAKACQ
SvELF3b  219 NTDKTHLEGMKVSDAISMGSPGIKEKEPTKVRIDLEIEERTSSFQTSKEKSGRLDPKVSS

AtELF3   221 SHDRVNDYNASLRQESRNRLYRDGGKTR--------LKDTDNGAESHLATENHSQEGHGSP
BdELF3   268 HRDKLSDLNVFDKQHARTEVHQARRTNENAAESQNAPKAGNGPSS---TNV---ERNGAS
SvELF3a  276 MGDNATNIDSYDNPHFGNSRRQPTSMNGSSMEAKNPTTTRNTVSCKPCTDLN--DSNKNS
SvELF3b  279 YRDKLNKYNVADKQSSEIASYQTRNRKENAGETQNPPEAEMAPSAKPYAGM---EQNGNS

AtELF3   274 EDIDND-REYSKSRACASLQQINEEASDDVSDDSMVDSISSIDVSPDDVVGILGQKRFWR
BdELF3   322 NLLEKGLRVTGEKRK--RSEGHHNV-QKDDSSDLSVESLPGLEISPDDVVGAIGPKHFWK
SvELF3a  334 NLLDRSLREAGSKRK--RGHQ-DVE-QNDDLSDSSVECIPGWEVSPDEIVGAIGPKHFWK
SvELF3b  336 DLLELGLRETGEKRK--RSHH-GVE-HNDDLSDSSVESIPEMEISPDDVVSAIGPKHFWK

AtELF3   333 ARKAIANQQRVFAVQLFELHRLIKVQKLIAASPDLLLDEISFLGKVSAKSYPVKKLLPSE
BdELF3   379 ARRAIVNQQRVFAVQVFELHRLIKVQKLIAASPHLLIEGDPCLGSALATSK---KKLAAG
SvELF3a  390 ARKAIQNQQRVFAVQVFELHKLIKVQKLIAASPHLLIEGDPVLGSALVGKK---TKLPKG
SvELF3b  392 ARRAIVNQQRVFAVQVFELHRLIKVQKLIAASPHLLIEGDPCLDKALAASK---KKLAGG

AtELF3   393 FLVKPPLPHVVVKQRGDSEKTDQHKMESSAENVV----------GRLSNQGHHQQSNYMPF
BdELF3   436 NVEKQPP---SAKNKDDAQLT-LQQVEYSKDNIEGNQASPSQDDVVVQHNNQAASNGGD
SvELF3a  447 NLKVQTL---SIANKDDIQPT-PEQPELSKQNTEGNPPSPCRDDGLGGNGHDQAAINETF
SvELF3b  449 DAEKQHQ---SAKYKDDVQQT-LQQLEHSKDNTEAAQPSPTQDDVVAAQHNNQAAATAAV

AtELF3   444 ANNPPASPAP-----NGYCFPPQPPPSGNHQQWLIPVMSPSEGLIYKPHPGMAHTG--HY
BdELF3   492 TSNPPAIPAAPDNKQSNWCTP-P-------QNQWLVPVMSPSEGLVYKPYTGPCPPAGSFL
SvELF3a  503 TSNPPVMPAAPDNKQNNWCMNPP-------QNQWLVPVMSPSEGLVYKPYAGPCPPVGSLL
SvELF3b  505 NSNPPTMPTPSDNKQNSWCIPPP-------PNQWLVPVMSPSEGLVYKPYAGHCPPAGSFL

AtELF3   497 GGYY-------------GHYMPTPMVM--PQYHPGMGF----PPPGNGYFPPYGMMPTIMN
BdELF3   545 APFYASCAPLSLPSTAGDFMNSPYGIHMPHQPQHMGLGGP-PPMPPMYFPPFSMP--VMN
SvELF3a  557 APFYANCTPLKLPS-------TPYGVPMPHQPQHMAPPGA-PAMHMNYFPPFSVP--VMN
SvELF3b  559 APFYPSCAPVSLPSTAGDFMSSPYGIPMPHQPQHMGVPGPPPPMPPMYFPPFSMP--VMN

AtELF3   539 PYCSSQQQQQ------QPNEQMNQFGHPG-NLQNTQQQQQRSDNEPAPQQQQQPTKSYP
BdELF3   602 PVVSASAVEQVSRIAPARPNAHVEHYSRNSCNM---------RNEAMSAGI------WRFH
SvELF3a  607 PGAPASAVEQGSHAAVPQPHGRAEQQSLISCNMSH-----------PSGI------WRFH
SvELF3b  617 TAVSASAVEQVSHVAASRPNGHIEQHSRSSCNMSN-----LRSEALSADI------WRFH

AtELF3   592 RARKS-RQG-STGSSPSGPQGISG--SKSFRPFAAVDEDSNINNAPEQTMTTTTTTTRTT
BdELF3   648 ASRDSELQASSAASSPFDRQQGEARGPAAPPPIPTS----SAGNG---------------Q
SvELF3a  650 ASRDREPQASS-ASSPFDRIQVQGDGSGLVSVFPSA----PAQNA---------------Q
SvELF3b  666 ASKDSELQGSS-ASSTFDRQQGEGRGPAQ--PFPSS----SVGNG---------------Q

AtELF3   648 VTQTTRDGGGVTRVIKVVPHNAKLASENAARIFQSIQEERKRYDSSKP
BdELF3   690 PQPSTGSKENPAGVIRVVPHTARTASESAARIFRSIQMERQQNDP---
SvELF3a  691 PQPSSGSRDQQNHVIRVVPHNAQTASESAARIFRSIQMERRQNDP---
SvELF3b  705 PQPSSGSRENPGRVIRVVPHTSRTASESAARIFESIKMERQQND----
```

B

AtELF3	AT2G25930.1
BdELF3	Bradi2g14290.1
SvELF3a	Sevir.5G206400.1
SvELF3b	Sevir.3G123200.1

	AtELF3	BdELF3	SvELF3a	SvELF3b
AtELF3	100			
BdELF3	36.79	100		
SvELF3a	34.74	57.36	100	
SvELF3b	35.81	74.31	56.75	100

Supplemental Figure 2

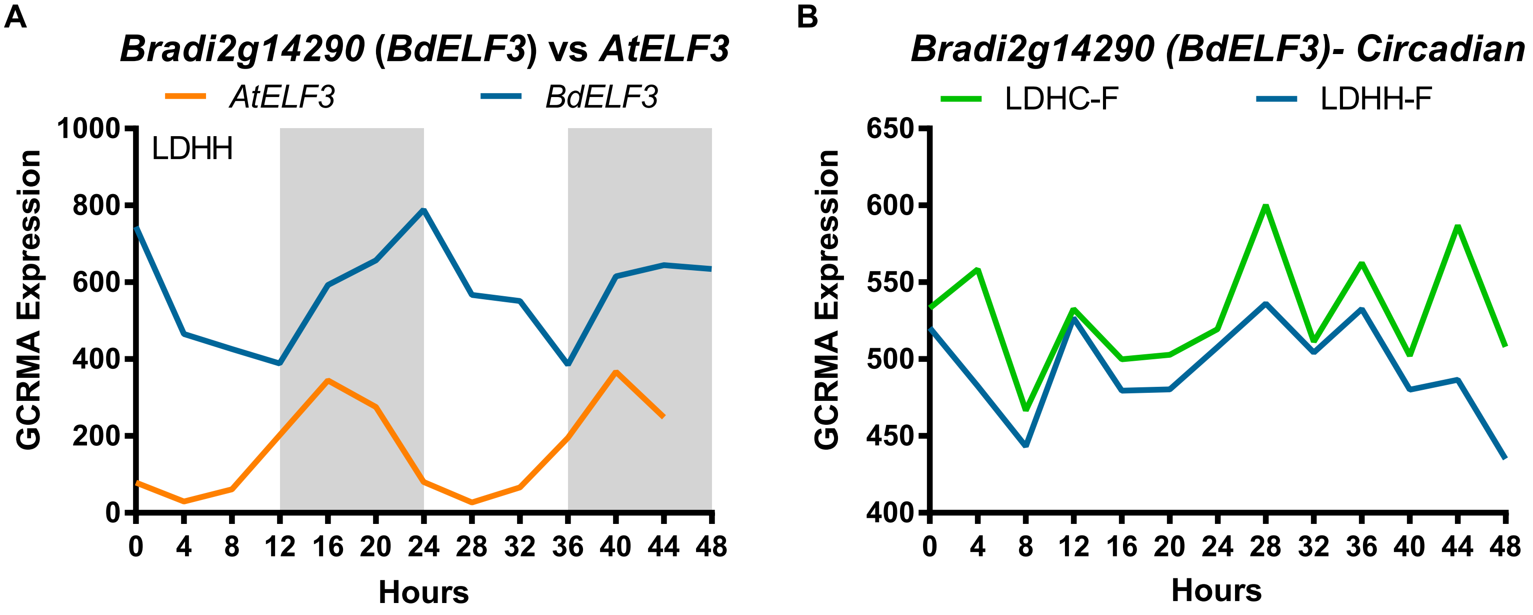

Supplemental Figure 3

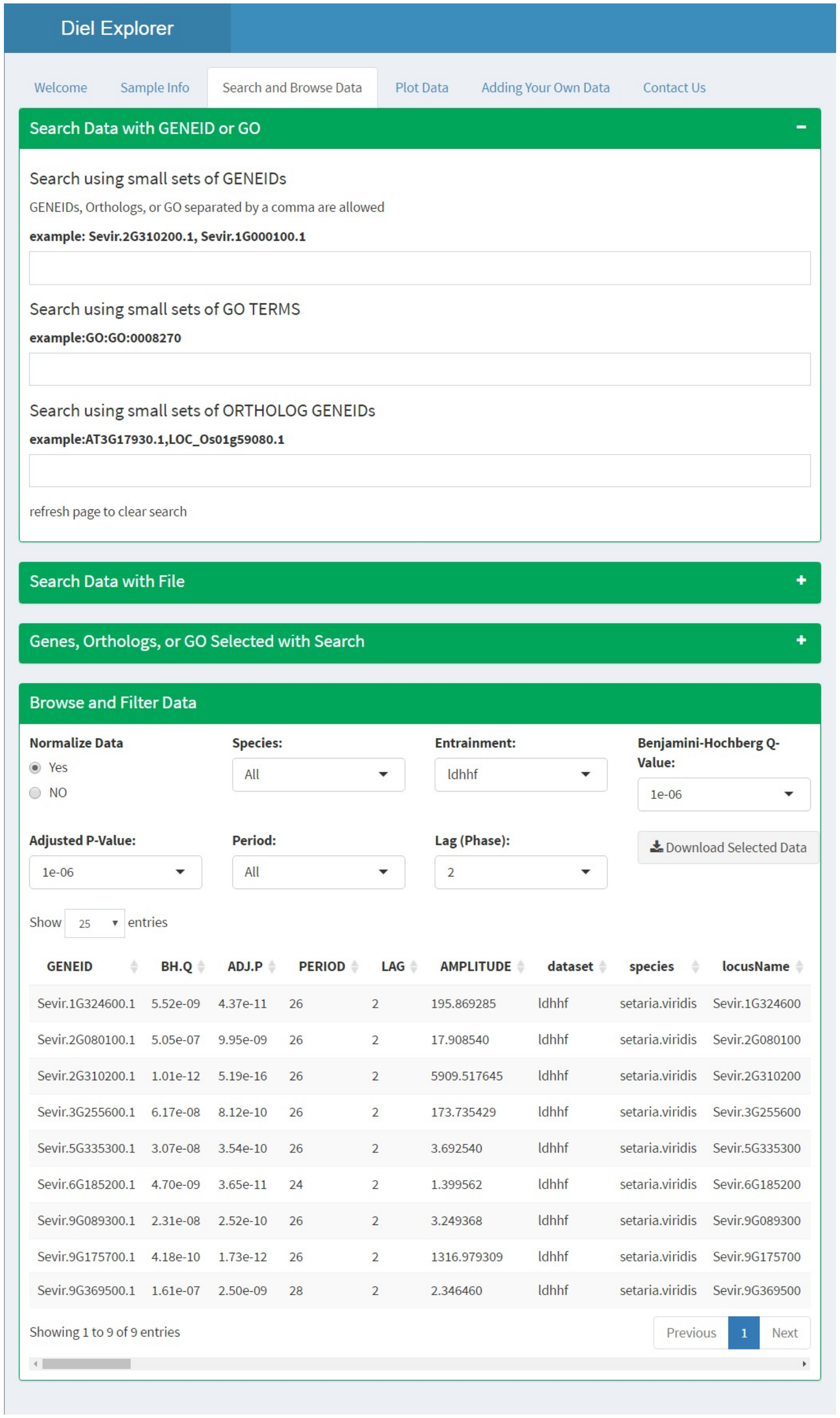

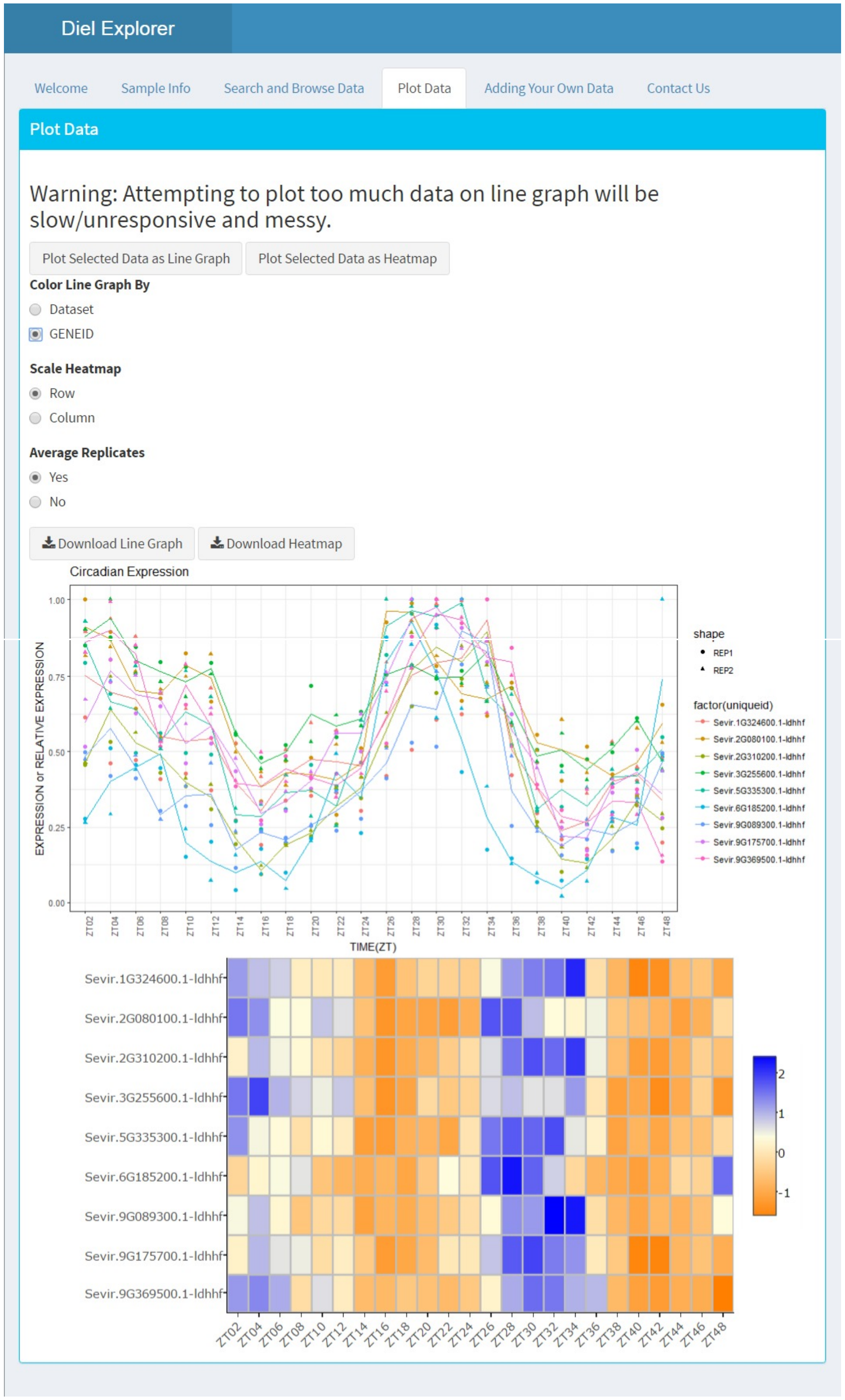

Supplemental Figure 4

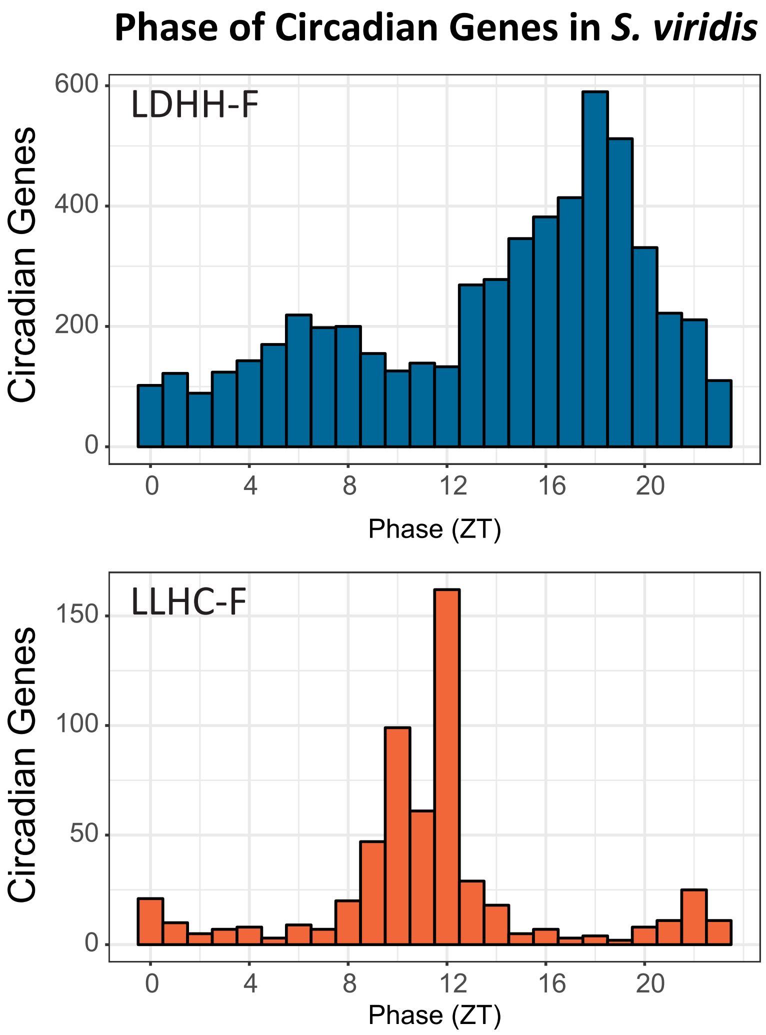

Supplemental Figure 5

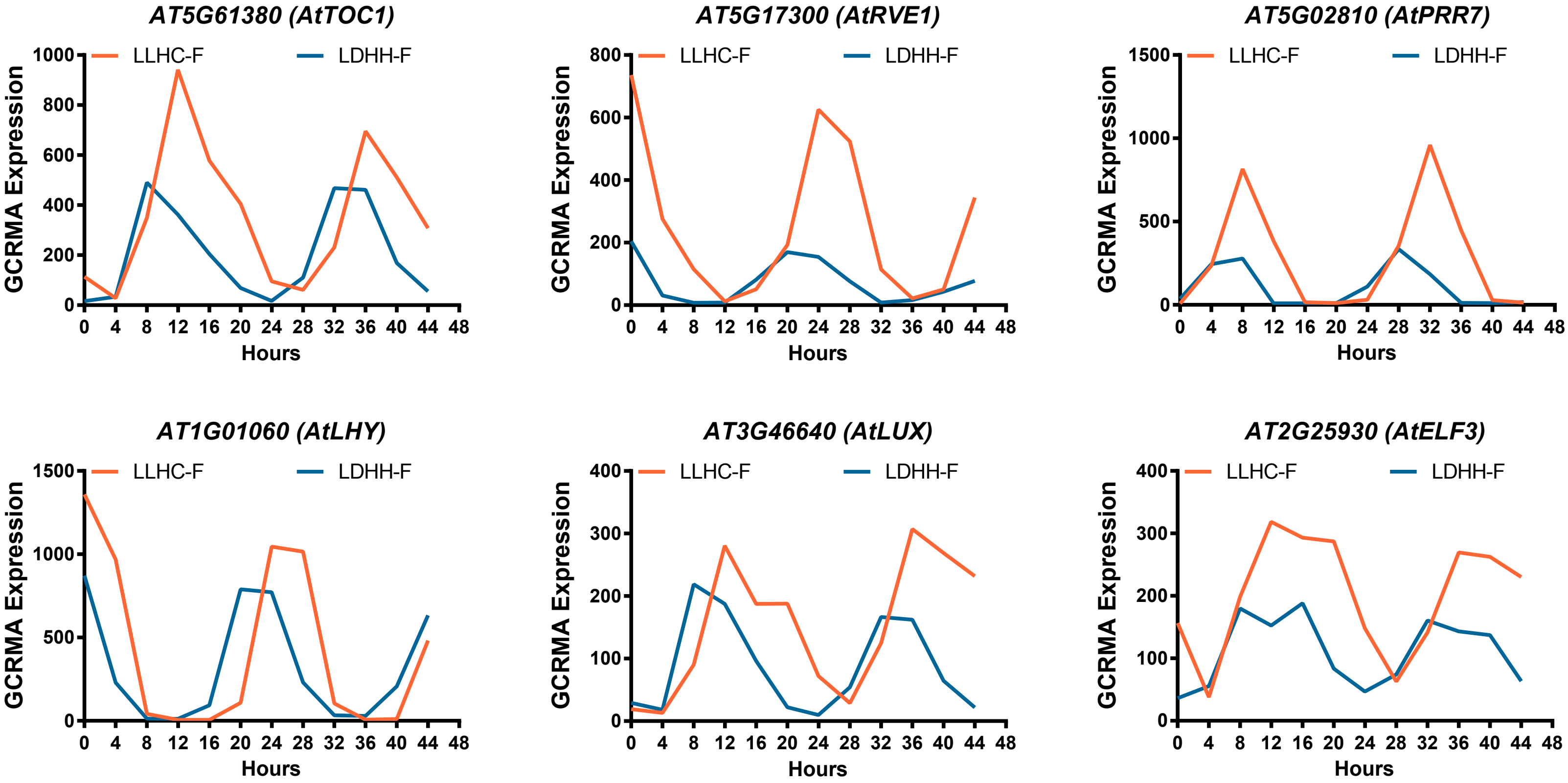

Supplemental Figure 6

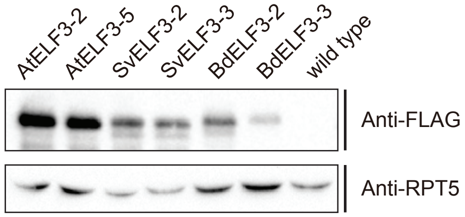

Supplemental Figure 7

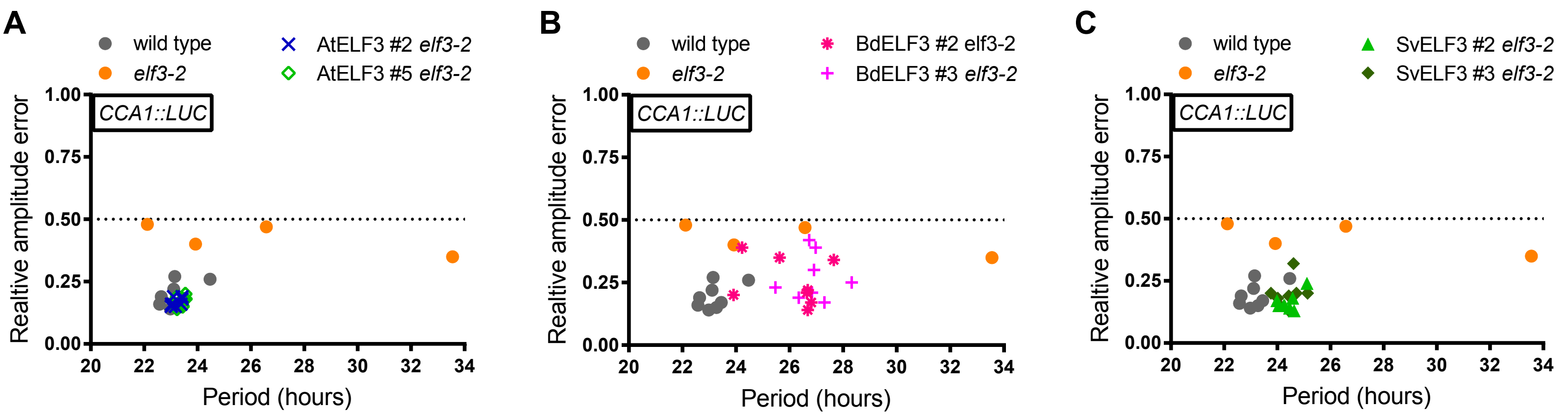

Supplemental Table 1. List of all primers used.

**Primers used for cloning At/Bd/SvELF3 coding sequence (CDS) without any stop codon [a]**		
Amplified Fragments	Forward primer (5'->3')	Reverse primer (5'->3')
AtELF3	*CACCATGAAGAGAGGGAAAGATGAG*	*AGGCTTAGAGGAGTCATAGCG*
SvELF3	*CACCATGACGAGGGGAGGTG*	*TGGGTCATTTTGTCGCCTCTCC*

**Primers used to generate yeast two-hybrid constructs, all with a stop codon [a]**		
Amplified fragments	Forward primer (5'->3')	Reverse primer (5'->3')
BdELF3-stop	*CACCATGAGGAGGGGCGGCGG*	*TTACGGATCATTCTGTTGCCTCTCC*
SvELF3-stop	*CACCATGACGAGGGGAGGTG*	*CTATGGGTCATTTTGTCGCCTCTCC*
AtELF4	*CACCATGAAGAGGAACGGCGAGACGA*	*TTAAGCTCTAGTTCCGGCAGCACC*
AtLUX-Nt (amino acids 1-143)	*CACCATGGGAGAGGAAGTACAAA*	*CTATTTAAGTGTTTTCCCAGATAG*
AtLUX-Ct (amino acids 144-324)	*CACCATGCGACCGCGTTTAGTGTGGACA*	*TTAATTCTCATTTGCGCTTCCACCT*

**Primer sets for genotyping**		
Mutant name	for wild type PCR (5'->3')	for mutant PCR (5'->3')
elf3-2 [b]	*TGAGTATTTGTTTCTTCTCGAGC* and CATATGGAGGGAAGTAGCCATTAC	*TGGTTATTTATTCTCCGCTCTTTC* and *TTGTTCCATTAGCTGTTCAACCTA*

[a] CACC (underscored) were added to forward primers for cloning into the pENTR/D-TOPO vector

[b] (Nusinow et al., 2011)

Supplemental Table 2. A full list of At/Bd/SvELF3 associated proteins identified from AP-MS

Total Unique Peptide Count

Identified Proteins (283)	Accession Number	Molecular Weight	AtELF3-1	AtELF3-2	SvELF3#2 rep1	SvELF3#2 rep2	SvELF3#3 rep1	SvELF3#3 rep2	BdELF3#2 rep1	BdELF3#2 rep2	BdELF3#3 rep1	BdELF3#3 rep2
| Symbols: PHYB, HY3, OOP1 | phytochrome B | chr2:8140079-8144151 FORWARD LENGTH=1172	AT2G18790.1	129 kDa	33	34	41	40	43	40	31	31	36	35
| Symbols: PHYD | phytochrome D | chr4:9195602-9199486 REVERSE LENGTH=1164	AT4G16250.1	129 kDa	10	11	11	13	13	12	11	10	13	12
| Symbols: HSC70-1, HSP70-1, AT-HSC70-1, HSC70 | heat shock cognate protein 70-1 | chr5:554055-556334 REVERSE LENGTH=651	AT5G02500.1	71 kDa	15	12	25	22	24	30	12	12	7	9
| Symbols: | Heat shock protein 70 (Hsp 70) family protein | chr3:2903434-2905632 REVERSE LENGTH=649	AT3G09440.1 (+1)	71 kDa	16	15	22	21	22	26	15	14	6	10
| Symbols: HSP70, ATHSP70 | heat shock protein 70 | chr3:3991487-3993689 REVERSE LENGTH=650	AT3G12580.1	71 kDa	12	9	15	16	17	20	10	10	4	6
| Symbols: | Heat shock protein 70 (Hsp 70) family protein | chr5:550296-552565 REVERSE LENGTH=653	AT5G02490.1	71 kDa	13	11	13	13	14	18	10	9	4	6
| Symbols: Hsp70b | heat shock protein 70B | chr1:5502386-5504326 REVERSE LENGTH=646	AT1G16030.1	71 kDa	0	0	0	0	0	0	0	0	0	0
| Symbols: BdELF3_HFC	ATMGcustom02	86 kDa	1	0	1	0	1	0	26	27	22	20
| Symbols: PHYC | phytochrome C | chr5:14008049-14011619 FORWARD LENGTH=1111	AT5G35840.1	124 kDa	22	23	27	32	27	33	20	18	19	22
| Symbols: SvELF3_HFC	ATMGcustom01	87 kDa	1	0	20	29	23	33	1	1	1	1
| Symbols: AtELF3_HFC	ATMGcustom03	84 kDa	21	19	0	0	0	0	0	0	0	1
| Symbols: TZP | zinc knuckle (CCHC-type) family protein | chr5:17527261-17530207 FORWARD LENGTH=831	AT5G43630.1	91 kDa	14	12	13	20	13	24	12	13	14	14
| Symbols: RBCL | ribulose-bisphosphate carboxylases | chrC:54958-56397 REVERSE LENGTH=479	ATCG00490.1	53 kDa	14	13	13	18	11	19	14	14	9	10
| Symbols: PHYE | phytochrome E | chr4:10042312-10045948 REVERSE LENGTH=1112	AT4G18130.1	123 kDa	12	19	13	18	12	17	6	10	15	14
| Symbols: TUB2 | tubulin beta chain 2 | chr5:25181560-25183501 FORWARD LENGTH=450	AT5G62690.1 (+1)	51 kDa	10	9	2	7	3	9	7	5	4	3
| Symbols: TUB5 | tubulin beta-5 chain | chr1:6938033-6940481 REVERSE LENGTH=449	AT1G20010.1	50 kDa	9	7	0	7	0	9	7	4	3	2
| Symbols: TUB6 | beta-6 tubulin | chr5:3961317-3962971 REVERSE LENGTH=449	AT5G12250.1	51 kDa	7	6	0	4	0	6	6	4	3	2
| Symbols: TUB9 | tubulin beta-9 chain | chr4:11182218-11183840 FORWARD LENGTH=444	AT4G20890.1	50 kDa	9	8	0	6	2	7	5	4	3	2
| Symbols: TUB7 | tubulin beta-7 chain | chr2:12644258-12645932 REVERSE LENGTH=449	AT2G29550.1	51 kDa	9	8	0	5	3	8	6	5	3	2
| Symbols: TUB4 | tubulin beta chain 4 | chr5:17859442-17860994 REVERSE LENGTH=444	AT5G44340.1	50 kDa	9	8	0	5	2	7	6	4	3	2
| Symbols: TUB8 | tubulin beta 8 | chr5:8042962-8044528 FORWARD LENGTH=449	AT5G23860.1 (+1)	51 kDa	9	8	0	6	3	6	6	5	3	0
| Symbols: PCH1 | F-box family protein | chr2:7074940-7079181 FORWARD LENGTH=778	AT2G16365.1 (+2)	89 kDa	9	9	9	14	11	16	9	9	11	11
| Symbols: GAPC-2, GAPC2 | glyceraldehyde-3-phosphate dehydrogenase C2 | chr1:4608465-4610494 REVERSE LENGTH=338	AT1G13440.1	37 kDa	8	7	4	15	6	12	4	3	6	2
| Symbols: GAPC, GAPC-1, GAPC1 | glyceraldehyde-3-phosphate dehydrogenase C subunit 1 | chr3:1081077-1083131 FORWARD LENGTH=338	AT3G04120.1	37 kDa	7	7	4	15	6	12	4	3	6	2
| Symbols: ATPA | ATP synthase subunit alpha | chrC:9938-11461 REVERSE LENGTH=507	ATCG00120.1	55 kDa	12	9	1	10	0	13	9	8	7	4
| Symbols: TUA4, TOR2 | tubulin alpha-4 chain | chr1:1356421-1358266 REVERSE LENGTH=450	AT1G04820.1 (+1)	50 kDa	6	7	5	9	6	10	2	5	2	1
| Symbols: TUA3 | tubulin alpha-3 | chr5:6682761-6684474 REVERSE LENGTH=450	AT5G19770.1 (+1)	50 kDa	4	4	3	6	3	8	1	3	1	0
| Symbols: TUA6 | Tubulin/FtsZ family protein | chr4:8548753-8550319 REVERSE LENGTH=427	AT4G14960.1	47 kDa	6	6	4	8	5	8	2	6	2	1
| Symbols: ACT8 | actin 8 | chr1:18216539-18217947 FORWARD LENGTH=377	AT1G49240.1 (+1)	42 kDa	3	3	2	5	3	4	3	3	3	3
| Symbols: ACT7 | actin 7 | chr5:3052809-3054220 FORWARD LENGTH=377	AT5G09810.1	42 kDa	4	4	1	6	4	6	4	3	2	1
| Symbols: ACT1, AAc1 | actin 1 | chr2:15779761-15781241 FORWARD LENGTH=377	AT2G37620.1 (+2)	42 kDa	1	0	0	4	1	2	0	0	0	0
| Symbols: ACT4 | actin 4 | chr5:23950109-23951586 FORWARD LENGTH=377	AT5G59370.1 (+1)	42 kDa	0	0	0	4	0	3	1	1	1	0
| Symbols: | Actin family protein | chr2:17578683-17580222 FORWARD LENGTH=329	AT2G42170.1	37 kDa	1	1	1	1	1	1	1	1	1	0
| Symbols: | Actin-like ATPase superfamily protein | chr2:17560211-17561945 FORWARD LENGTH=378	AT2G42100.1	42 kDa	0	0	0	0	0	0	0	0	0	0
| Symbols: ATRAB8D, ATRABE1B, RABE1b | RAB GTPase homolog E1B | chr4:10990036-10991466 FORWARD LENGTH=476	AT4G20360.1	52 kDa	6	5	9	10	8	12	5	4	3	3
| Symbols: | Ribosomal protein S5 family protein | chr2:17460016-17461398 REVERSE LENGTH=285	AT2G41840.1	31 kDa	5	6	2	3	3	4	5	5	2	1
| Symbols: XW6 | Ribosomal protein S5 family protein | chr1:21689115-21690085 FORWARD LENGTH=284	AT1G58380.1 (+3)	31 kDa	6	5	3	2	5	5	3	3	1	0
| Symbols: | Ribosomal protein S5 family protein | chr3:21279824-21280887 REVERSE LENGTH=276	AT3G57490.1	30 kDa	3	0	0	0	0	0	0	0	0	0
| Symbols: TIC | time for coffee | chr3:7913181-7918967 FORWARD LENGTH=1555	AT3G22380.2	165 kDa	5	5	4	12	3	15	4	3	1	1
| Symbols: ELF4 | Protein of unknown function (DUF1313) | chr2:16734545-16734880 REVERSE LENGTH=111	AT2G40080.1	12 kDa	4	4	3	5	4	4	4	4	5	5
| Symbols: GAPA, GAPA-1 | glyceraldehyde 3-phosphate dehydrogenase A subunit | chr3:9795226-9796848 FORWARD LENGTH=396	AT3G26650.1	42 kDa	11	8	3	13	4	15	5	4	3	2
| Symbols: GAPA-2 | glyceraldehyde 3-phosphate dehydrogenase A subunit 2 | chr1:4392634-4393850 REVERSE LENGTH=350	AT1G12900.3 (+1)	38 kDa	11	8	3	11	4	12	5	3	2	1
| Symbols: | Ribosomal protein L6 family | chr1:12010986-12012223 FORWARD LENGTH=194	AT1G33120.1 (+1)	22 kDa	4	4	6	10	5	9	4	5	5	5
| Symbols: | Ribosomal protein L6 family | chr4:6463201-6464458 REVERSE LENGTH=194	AT4G10450.1	22 kDa	1	2	0	3	0	3	0	2	2	2
| Symbols: COP1, ATCOP1, DET340, FUS1, EMB168 | Transducin/WD40 repeat-like superfamily protein | chr2:13978000-13983282 FORWARD LENGTH=675	AT2G32950.1	76 kDa	8	9	6	4	3	5	1	2	6	4
| Symbols: IMPA-2 | importin alpha isoform 2 | chr4:9134450-9137134 REVERSE LENGTH=535	AT4G16143.1 (+1)	59 kDa	2	2	1	8	0	8	10	9	2	4
| Symbols: AT-IMP, ATKAP ALPHA, AIMP ALPHA, IMPA-1, IMPA1 | importin alpha isoform 1 | chr3:2120559-2123555 FORWARD LENGTH=532	AT3G06720.1 (+1)	59 kDa	0	0	0	2	0	2	2	1	0	0
| Symbols: IMPA-6 | importin alpha isoform 6 | chr1:584397-587036 FORWARD LENGTH=538	AT1G02690.1 (+1)	59 kDa	0	0	0	0	0	0	0	0	0	0
| Symbols: IMPA-4 | importin alpha isoform 4 | chr1:2994506-2997833 FORWARD LENGTH=538	AT1G09270.1 (+2)	59 kDa	0	0	0	0	0	0	0	0	0	0
| Symbols: MTO3, SAMS3, MAT4 | S-adenosylmethionine synthetase family protein | chr3:5952484-5953665 REVERSE LENGTH=393	AT3G17390.1	43 kDa	9	7	2	5	0	6	1	0	0	0
| Symbols: MAT3 | methionine adenosyltransferase 3 | chr2:15479721-15480893 REVERSE LENGTH=390	AT2G36880.1 (+1)	42 kDa	4	2	2	1	0	2	0	0	0	0
| Symbols: SAM-2, MAT2, SAM2, AtSAM2 | S-adenosylmethionine synthetase 2 | chr4:796298-797479 REVERSE LENGTH=393	AT4G01850.1 (+1)	43 kDa	5	2	0	1	0	2	0	0	0	0
| Symbols: SAM1, SAM-1, MAT1, AtSAM1 | S-adenosylmethionine synthetase 1 | chr1:519037-520218 FORWARD LENGTH=393	AT1G02500.1 (+1)	43 kDa	4	1	0	2	0	3	0	0	0	0
| Symbols: RCA | rubisco activase | chr2:16570951-16573345 REVERSE LENGTH=474	AT2G39730.1	52 kDa	7	7	3	4	3	6	7	6	3	2
| Symbols: PCL1, LUX | Homeodomain-like superfamily protein | chr3:17183248-17184219 FORWARD LENGTH=323	AT3G46640.1 (+1)	35 kDa	1	1	3	6	3	7	1	1	1	3
| Symbols: GS2, GLN2, ATGSL1 | glutamine synthetase 2 | chr5:13831220-13833239 FORWARD LENGTH=430	AT5G35630.1 (+2)	47 kDa	6	5	3	8	3	7	5	4	1	1
| Symbols: CAT3, SEN2, ATCAT3 | catalase 3 | chr1:7143142-7146193 FORWARD LENGTH=485	AT1G20620.5	56 kDa	13	11	2	8	1	9	7	4	1	1
| Symbols: | Clathrin, heavy chain | chr3:3482575-3491667 REVERSE LENGTH=1705	AT3G11130.1	193 kDa	16	10	0	2	0	5	2	1	0	0
| Symbols: | Clathrin, heavy chain | chr3:2587171-2595411 REVERSE LENGTH=1703	AT3G08530.1	193 kDa	15	9	0	2	0	5	2	1	0	0
| Symbols: | Ribosomal protein S3 family protein | chr3:19951547-19952782 FORWARD LENGTH=249	AT3G53870.1	27 kDa	4	4	3	4	3	5	2	2	2	1
| Symbols: | Ribosomal protein S3 family protein | chr2:13450384-13451669 FORWARD LENGTH=250	AT2G31610.1	28 kDa	5	5	3	4	3	6	3	2	1	1
| Symbols: | Ribosomal protein S3 family protein | chr5:13710355-13712192 REVERSE LENGTH=248	AT5G35530.1	27 kDa	5	5	3	4	3	5	3	1	1	1
| Symbols: EIF4A1, RH4, TIF4A1 | eukaryotic translation initiation factor 4A1 | chr3:4592635-4594128 REVERSE LENGTH=412	AT3G13920.1 (+1)	47 kDa	7	6	3	5	5	7	3	2	2	2
| Symbols: EIF4A-2 | eif4a-2 | chr1:20260495-20262018 FORWARD LENGTH=412	AT1G54270.1	47 kDa	5	4	3	5	5	7	3	2	2	2
| Symbols: EIF4A-III | eukaryotic initiation factor 4A-III | chr3:6863790-6866242 FORWARD LENGTH=408	AT3G19760.1	?	0	0	0	0	0	0	0	0	0	0
| Symbols: BIP, BIP2 | Heat shock protein 70 (Hsp 70) family protein | chr5:16807697-16810480 REVERSE LENGTH=668	AT5G42020.1	74 kDa	3	5	5	9	7	11	1	2	3	2
| Symbols: DAYSLEEPER | BED zinc finger ;hAT family dimerisation domain | chr3:14321838-14323928 FORWARD LENGTH=696	AT3G42170.1	79 kDa	8	5	0	7	0	12	3	4	2	0
MLK2 | Symbols: | Protein kinase family protein | chr3:1014412-1018244 REVERSE LENGTH=701	AT3G03940.1	78 kDa	3	4	0	3	0	1	8	6	6	5
MLK1 | Symbols: | Protein kinase family protein | chr5:6010215-6013724 REVERSE LENGTH=691	AT5G18190.1	77 kDa	5	5	0	2	0	1	6	6	5	5
| Symbols: GAPB | glyceraldehyde-3-phosphate dehydrogenase B subunit | chr1:16127552-16129584 FORWARD LENGTH=447	AT1G42970.1	48 kDa	9	7	1	11	1	15	5	4	3	1
| Symbols: SPA1 | SPA (suppressor of phyA-105) protein family | chr2:19022572-19026821 REVERSE LENGTH=1029	AT2G46340.1	115 kDa	8	7	2	4	2	4	0	0	6	5
| Symbols: | Ribulose bisphosphate carboxylase (small chain) family protein | chr5:15377501-15378306 REVERSE LENGTH=181	AT5G38410.1	20 kDa	5	4	4	7	5	7	5	3	3	2
| Symbols: | Ribulose bisphosphate carboxylase (small chain) family protein | chr5:15384350-15385155 REVERSE LENGTH=181	AT5G38430.1	20 kDa	4	3	2	3	2	3	3	1	2	1
| Symbols: PHYA, FHY2, FRE1, HY8 | phytochrome A | chr1:3095498-3099216 REVERSE LENGTH=1122	AT1G09570.1	125 kDa	1	1	7	5	7	2	0	2	6	3
| Symbols: | Ribosomal protein L4/L1 family | chr3:2953813-2955444 FORWARD LENGTH=406	AT3G09630.1 (+1)	45 kDa	1	1	1	2	1	7	2	1	1	0
| Symbols: | Ribosomal protein L4/L1 family | chr5:657830-659526 FORWARD LENGTH=407	AT5G02870.1 (+1)	45 kDa	2	2	1	2	1	6	2	1	0	0
| Symbols: P5CS1, ATP5CS | delta1-pyrroline-5-carboxylate synthase 1 | chr2:16598516-16602939 REVERSE LENGTH=717	AT2G39800.1 (+1)	78 kDa	7	6	1	4	1	9	4	3	2	0
| Symbols: P5CS2 | delta 1-pyrroline-5-carboxylate synthase 2 | chr3:20624278-20628989 REVERSE LENGTH=726	AT3G55610.1	79 kDa	0	0	0	0	0	0	0	0	0	0
| Symbols: RBCS1A | ribulose bisphosphate carboxylase small chain 1A | chr1:25048465-25049249 REVERSE LENGTH=180	AT1G67090.1	20 kDa	5	5	4	7	5	7	4	3	1	3
| Symbols: ATCIMS, ATMETS, ATMS1 | Cobalamin-independent synthase family protein | chr5:5935771-5939195 FORWARD LENGTH=765	AT5G17920.1 (+1)	84 kDa	4	5	3	5	2	7	2	1	0	0
| Symbols: ATMS3, MS3 | methionine synthase 3 | chr5:7124397-7128353 REVERSE LENGTH=812	AT5G20980.1 (+1)	91 kDa	2	2	0	0	0	2	1	0	0	0

<table>
<tr><td>| Symbols: PGK1 | phosphoglycerate kinase 1 | chr3:4061127-4063140 REVERSE LENGTH=481</td><td>AT3G12780.1</td><td>50 kDa</td><td>5</td><td>4</td><td>0</td><td>6</td><td>0</td><td>9</td><td>3</td><td>2</td><td>1</td><td>0</td></tr>
<tr><td>| Symbols: PGK | phosphoglycerate kinase | chr1:29924347-29926295 REVERSE LENGTH=401</td><td>AT1G79550.1 (+1)</td><td>42 kDa</td><td>2</td><td>0</td><td>0</td><td>0</td><td>0</td><td>0</td><td>1</td><td>1</td><td>1</td><td>0</td></tr>
<tr><td>| Symbols: | Phosphoglycerate kinase family protein | chr1:21028622-21030454 FORWARD LENGTH=405</td><td>AT1G56190.2</td><td>43 kDa</td><td>3</td><td>0</td><td>0</td><td>3</td><td>0</td><td>4</td><td>0</td><td>0</td><td>0</td><td>0</td></tr>
<tr><td>| Symbols: LOS1 | Ribosomal protein S5/Elongation factor G/III/V family protein | chr1:20968245-20971077 REVERSE LENGTH=843</td><td>AT1G56070.1</td><td>94 kDa</td><td>4</td><td>3</td><td>1</td><td>3</td><td>2</td><td>9</td><td>1</td><td>1</td><td>0</td><td>0</td></tr>
<tr><td>| Symbols: | structural constituent of ribosome | chrC:82826-83482 REVERSE LENGTH=218</td><td>ATCG00800.1</td><td>25 kDa</td><td>6</td><td>4</td><td>1</td><td>4</td><td>1</td><td>6</td><td>1</td><td>0</td><td>0</td><td>0</td></tr>
<tr><td>| Symbols: CAT2 | catalase 2 | chr4:16700937-16703215 REVERSE LENGTH=492</td><td>AT4G35090.1</td><td>57 kDa</td><td>10</td><td>6</td><td>2</td><td>8</td><td>1</td><td>7</td><td>4</td><td>4</td><td>1</td><td>1</td></tr>
<tr><td>| Symbols: | Ribosomal protein S27a / Ubiquitin family protein | chr1:8314940-8315410 FORWARD LENGTH=156</td><td>AT1G23410.1 (+3)</td><td>18 kDa</td><td>3</td><td>3</td><td>1</td><td>2</td><td>1</td><td>2</td><td>3</td><td>2</td><td>1</td><td>2</td></tr>
<tr><td>| Symbols: UBQ9 | ubiquitin 9 | chr5:14952782-14953750 REVERSE LENGTH=322</td><td>AT5G37640.1</td><td>36 kDa</td><td>0</td><td>2</td><td>0</td><td>0</td><td>0</td><td>0</td><td>0</td><td>0</td><td>0</td><td>0</td></tr>
<tr><td>| Symbols: UBQ12 | ubiquitin 12 | chr1:20549533-20550225 FORWARD LENGTH=230</td><td>AT1G55060.1</td><td>26 kDa</td><td>0</td><td>0</td><td>0</td><td>0</td><td>0</td><td>0</td><td>0</td><td>0</td><td>0</td><td>0</td></tr>
<tr><td>| Symbols: UBQ8 | ubiquitin 8 | chr3:3004111-3006006 REVERSE LENGTH=631</td><td>AT3G09790.1</td><td>72 kDa</td><td>0</td><td>0</td><td>0</td><td>0</td><td>0</td><td>0</td><td>0</td><td>0</td><td>0</td><td>0</td></tr>
<tr><td>| Symbols: | GTP binding Elongation factor Tu family protein | chr1:2455559-2457001 FORWARD LENGTH=449</td><td>AT1G07920.1 (+5)</td><td>50 kDa</td><td>4</td><td>3</td><td>1</td><td>4</td><td>1</td><td>6</td><td>3</td><td>1</td><td>2</td><td>1</td></tr>
<tr><td>| Symbols: | Ribosomal protein L13 family protein | chr3:9064613-9065871 FORWARD LENGTH=206</td><td>AT3G24830.1</td><td>23 kDa</td><td>1</td><td>1</td><td>1</td><td>1</td><td>2</td><td>1</td><td>1</td><td>2</td><td>0</td><td>0</td></tr>
<tr><td>| Symbols: | Ribosomal protein L13 family protein | chr3:2252092-2253332 FORWARD LENGTH=206</td><td>AT3G07110.1 (+1)</td><td>23 kDa</td><td>2</td><td>3</td><td>1</td><td>1</td><td>0</td><td>1</td><td>2</td><td>3</td><td>0</td><td>0</td></tr>
<tr><td>| Symbols: | Ribosomal protein L13 family protein | chr5:19771315-19772686 REVERSE LENGTH=206</td><td>AT5G48760.1 (+1)</td><td>24 kDa</td><td>0</td><td>0</td><td>0</td><td>1</td><td>0</td><td>1</td><td>1</td><td>1</td><td>0</td><td>0</td></tr>
<tr><td>| Symbols: | Ribosomal protein L13 family protein | chr4:7655133-7656542 REVERSE LENGTH=206</td><td>AT4G13170.1</td><td>24 kDa</td><td>0</td><td>2</td><td>0</td><td>1</td><td>0</td><td>0</td><td>0</td><td>2</td><td>0</td><td>0</td></tr>
<tr><td>| Symbols: HOG1, EMB1395, SAHH1, MEE58, ATSAHH1 | S-adenosyl-L-homocysteine hydrolase | chr4:8054931-8056676 FORWARD LENGTH=485</td><td>AT4G13940.1</td><td>53 kDa</td><td>3</td><td>4</td><td>1</td><td>3</td><td>1</td><td>5</td><td>1</td><td>2</td><td>0</td><td>0</td></tr>
<tr><td>| Symbols: SAHH2, ATSAHH2 | S-adenosyl-l-homocysteine (SAH) hydrolase 2 | chr3:8588013-8589671 REVERSE LENGTH=485</td><td>AT3G23810.1</td><td>53 kDa</td><td>2</td><td>2</td><td>0</td><td>1</td><td>0</td><td>3</td><td>0</td><td>1</td><td>0</td><td>0</td></tr>
<tr><td>| Symbols: HOG1, SAHH1 | S-adenosyl-L-homocysteine hydrolase | chr4:8054931-8056763 FORWARD LENGTH=325</td><td>AT4G13940.4</td><td>36 kDa</td><td>0</td><td>0</td><td>0</td><td>0</td><td>0</td><td>0</td><td>0</td><td>0</td><td>0</td><td>0</td></tr>
<tr><td>| Symbols: CRB, CSP41B, HIP1.3 | chloroplast RNA binding | chr1:3015473-3018035 FORWARD LENGTH=378</td><td>AT1G09340.1</td><td>43 kDa</td><td>4</td><td>3</td><td>1</td><td>1</td><td>1</td><td>4</td><td>1</td><td>1</td><td>0</td><td>0</td></tr>
<tr><td>| Symbols: ACC1, AT-ACC1, EMB22, GK, PAS3 | acetyl-CoA carboxylase 1 | chr1:13534196-13543773 FORWARD LENGTH=2254</td><td>AT1G36160.1 (+1)</td><td>251 kDa</td><td>12</td><td>1</td><td>0</td><td>1</td><td>0</td><td>2</td><td>0</td><td>0</td><td>0</td><td>0</td></tr>
<tr><td>| Symbols: | Ribosomal protein S5 domain 2-like superfamily protein | chr5:6090253-6090693 REVERSE LENGTH=146</td><td>AT5G18380.1 (+1)</td><td>17 kDa</td><td>3</td><td>4</td><td>1</td><td>3</td><td>1</td><td>2</td><td>3</td><td>3</td><td>0</td><td>0</td></tr>
<tr><td>| Symbols: | Ribosomal protein S5 domain 2-like superfamily protein | chr2:3781442-3781882 FORWARD LENGTH=146</td><td>AT2G09990.1</td><td>17 kDa</td><td>2</td><td>3</td><td>0</td><td>2</td><td>0</td><td>1</td><td>2</td><td>2</td><td>0</td><td>0</td></tr>
<tr><td>| Symbols: CA1 | carbonic anhydrase 1 | chr3:194853-197873 REVERSE LENGTH=347</td><td>AT3G01500.2 (+1)</td><td>37 kDa</td><td>2</td><td>2</td><td>1</td><td>3</td><td>1</td><td>5</td><td>3</td><td>1</td><td>0</td><td>0</td></tr>
<tr><td>| Symbols: CA2, CA18, BETA CA2 | carbonic anhydrase 2 | chr5:4758257-4762382 FORWARD LENGTH=331</td><td>AT5G14740.1 (+1)</td><td>37 kDa</td><td>1</td><td>1</td><td>1</td><td>3</td><td>1</td><td>6</td><td>1</td><td>0</td><td>0</td><td>0</td></tr>
<tr><td>| Symbols: ATAN11, LWD1 | Transducin/WD40 repeat-like superfamily protein | chr1:4395114-4396154 REVERSE LENGTH=346</td><td>AT1G12910.1</td><td>39 kDa</td><td>5</td><td>7</td><td>5</td><td>8</td><td>4</td><td>5</td><td>5</td><td>5</td><td>3</td><td>3</td></tr>
<tr><td>| Symbols: MLK4 | Protein kinase family protein | chr3:4469434-4473234 FORWARD LENGTH=703</td><td>AT3G13670.1</td><td>79 kDa</td><td>5</td><td>6</td><td>0</td><td>0</td><td>0</td><td>1</td><td>10</td><td>6</td><td>6</td><td>7</td></tr>
<tr><td>| Symbols: RPS6, RPS6A | ribosomal protein S6 | chr4:15346306-15347714 REVERSE LENGTH=250</td><td>AT4G31700.1</td><td>28 kDa</td><td>2</td><td>4</td><td>0</td><td>1</td><td>0</td><td>4</td><td>4</td><td>4</td><td>2</td><td>1</td></tr>
<tr><td>| Symbols: | Ribosomal protein L30/L7 family protein | chr2:132943-134264 REVERSE LENGTH=242</td><td>AT2G01250.1</td><td>28 kDa</td><td>4</td><td>3</td><td>1</td><td>0</td><td>1</td><td>2</td><td>1</td><td>2</td><td>1</td><td>0</td></tr>
<tr><td>| Symbols: | Ribosomal protein L30/L7 family protein | chr2:18249227-18250417 REVERSE LENGTH=247</td><td>AT2G44120.2</td><td>29 kDa</td><td>2</td><td>3</td><td>0</td><td>1</td><td>0</td><td>2</td><td>0</td><td>1</td><td>0</td><td>0</td></tr>
<tr><td>| Symbols: | Ribosomal protein L30/L7 family protein | chr3:4433809-4435109 FORWARD LENGTH=244</td><td>AT3G13580.1 (+2)</td><td>28 kDa</td><td>1</td><td>2</td><td>0</td><td>0</td><td>0</td><td>1</td><td>1</td><td>0</td><td>0</td><td>0</td></tr>
<tr><td>| Symbols: | Coatomer, alpha subunit | chr2:9152428-9156577 FORWARD LENGTH=1218</td><td>AT2G21390.1</td><td>136 kDa</td><td>2</td><td>0</td><td>0</td><td>2</td><td>0</td><td>9</td><td>0</td><td>0</td><td>0</td><td>0</td></tr>
<tr><td>| Symbols: | Coatomer, alpha subunit | chr1:22919814-22923728 FORWARD LENGTH=1216</td><td>AT1G62020.1</td><td>137 kDa</td><td>2</td><td>1</td><td>0</td><td>0</td><td>0</td><td>5</td><td>0</td><td>0</td><td>0</td><td>0</td></tr>
<tr><td>| Symbols: CAB3, AB180, LHCB1.2 | chlorophyll A/B binding protein 3 | chr1:10472443-10473246 REVERSE LENGTH=267</td><td>AT1G29910.1 (+1)</td><td>28 kDa</td><td>2</td><td>2</td><td>2</td><td>2</td><td>2</td><td>2</td><td>1</td><td>1</td><td>1</td><td>0</td></tr>
<tr><td>| Symbols: LHCB2.2, LHCB2 | photosystem II light harvesting complex gene 2.2 | chr2:1799436-1800329 REVERSE LENGTH=265</td><td>AT2G05070.1 (+2)</td><td>29 kDa</td><td>1</td><td>1</td><td>2</td><td>1</td><td>1</td><td>1</td><td>0</td><td>0</td><td>0</td><td>0</td></tr>
<tr><td>| Symbols: CAB1, AB140, CAB140, LHCB1.3 | chlorophyll A/B binding protein 1 | chr1:10478071-10478874 FORWARD LENGTH=267</td><td>AT1G29930.1</td><td>28 kDa</td><td>2</td><td>2</td><td>2</td><td>2</td><td>2</td><td>2</td><td>1</td><td>1</td><td>1</td><td>0</td></tr>
<tr><td>| Symbols: HSP60-2 | heat shock protein 60-2 | chr2:14075093-14078568 REVERSE LENGTH=585</td><td>AT2G33210.1 (+1)</td><td>62 kDa</td><td>1</td><td>3</td><td>1</td><td>3</td><td>1</td><td>2</td><td>1</td><td>0</td><td>0</td><td>0</td></tr>
<tr><td>| Symbols: HSP60, HSP60-3B | heat shock protein 60 | chr3:8669013-8672278 FORWARD LENGTH=577</td><td>AT3G23990.1</td><td>61 kDa</td><td>1</td><td>3</td><td>0</td><td>3</td><td>0</td><td>3</td><td>2</td><td>1</td><td>0</td><td>0</td></tr>
<tr><td>| Symbols: | Ribosomal protein S3Ae | chr3:1329751-1331418 FORWARD LENGTH=262</td><td>AT3G04840.1</td><td>30 kDa</td><td>2</td><td>2</td><td>1</td><td>1</td><td>1</td><td>3</td><td>1</td><td>1</td><td>1</td><td>0</td></tr>
<tr><td>| Symbols: | Ribosomal protein S3Ae | chr4:16548724-16550222 FORWARD LENGTH=262</td><td>AT4G34670.1</td><td>30 kDa</td><td>2</td><td>2</td><td>1</td><td>2</td><td>1</td><td>3</td><td>1</td><td>0</td><td>0</td><td>0</td></tr>
<tr><td>| Symbols: UBP12 | ubiquitin-specific protease 12 | chr5:2019545-2027834 REVERSE LENGTH=1116</td><td>AT5G06600.1 (+1)</td><td>131 kDa</td><td>0</td><td>0</td><td>0</td><td>3</td><td>0</td><td>3</td><td>1</td><td>1</td><td>0</td><td>0</td></tr>
<tr><td>| Symbols: UBP13 | ubiquitin-specific protease 13 | chr3:3761758-3770290 REVERSE LENGTH=1115</td><td>AT3G11910.1 (+1)</td><td>131 kDa</td><td>0</td><td>0</td><td>0</td><td>3</td><td>1</td><td>2</td><td>1</td><td>0</td><td>0</td><td>0</td></tr>
<tr><td>| Symbols: NIT1, ATNIT1, NITI | nitrilase 1 | chr3:15986901-15988841 FORWARD LENGTH=346</td><td>AT3G44310.1 (+1)</td><td>38 kDa</td><td>2</td><td>1</td><td>0</td><td>4</td><td>1</td><td>4</td><td>1</td><td>1</td><td>1</td><td>1</td></tr>
<tr><td>| Symbols: ARP1, emb2207, RPL3A, RP1 | ribosomal protein 1 | chr1:16266992-16268631 FORWARD LENGTH=389</td><td>AT1G43170.1 (+7)</td><td>45 kDa</td><td>2</td><td>1</td><td>0</td><td>2</td><td>0</td><td>1</td><td>1</td><td>1</td><td>0</td><td>0</td></tr>
<tr><td>| Symbols: RD21, RD21A | Granulin repeat cysteine protease family protein | chr1:17283139-17285609 REVERSE LENGTH=462</td><td>AT1G47128.1</td><td>51 kDa</td><td>4</td><td>3</td><td>0</td><td>2</td><td>0</td><td>2</td><td>0</td><td>2</td><td>0</td><td>0</td></tr>
<tr><td>| Symbols: ATPB, PB | ATP synthase subunit beta | chrC:52660-54156 REVERSE LENGTH=498</td><td>ATCG00480.1</td><td>54 kDa</td><td>4</td><td>5</td><td>0</td><td>3</td><td>0</td><td>3</td><td>2</td><td>2</td><td>1</td><td>0</td></tr>
<tr><td>| Symbols: PYK10, PSR3.1, BGLU23, LEB | Glycosyl hydrolase superfamily protein | chr3:2840657-2843730 REVERSE LENGTH=524</td><td>AT3G09260.1</td><td>?</td><td>1</td><td>1</td><td>1</td><td>3</td><td>0</td><td>3</td><td>1</td><td>1</td><td>0</td><td>0</td></tr>
<tr><td>| Symbols: KASI, KAS1 | 3-ketoacyl-acyl carrier protein synthase I | chr5:18774439-18776629 REVERSE LENGTH=473</td><td>AT5G46290.1</td><td>50 kDa</td><td>4</td><td>3</td><td>0</td><td>1</td><td>2</td><td>3</td><td>2</td><td>2</td><td>0</td><td>0</td></tr>
<tr><td>| Symbols: RPL2.1 | ribosomal protein L2 | chrC:84337-85843 REVERSE LENGTH=274</td><td>ATCG00830.1 (+1)</td><td>30 kDa</td><td>3</td><td>2</td><td>0</td><td>2</td><td>0</td><td>3</td><td>3</td><td>4</td><td>0</td><td>0</td></tr>
<tr><td>| Symbols: ASN2 | asparagine synthetase 2 | chr5:25969224-25972278 FORWARD LENGTH=578</td><td>AT5G65010.1 (+1)</td><td>65 kDa</td><td>3</td><td>2</td><td>0</td><td>1</td><td>0</td><td>4</td><td>2</td><td>2</td><td>0</td><td>0</td></tr>
<tr><td>| Symbols: ASN1, DIN6, AT-ASN1 | glutamine-dependent asparagine synthase 1 | chr3:17438445-17441043 REVERSE LENGTH=512</td><td>AT3G47340.2</td><td>58 kDa</td><td>0</td><td>0</td><td>0</td><td>0</td><td>0</td><td>0</td><td>0</td><td>0</td><td>0</td><td>0</td></tr>
<tr><td>| Symbols: ASN3 | asparagine synthetase 3 | chr5:3212934-3216418 REVERSE LENGTH=578</td><td>AT5G10240.1 (+1)</td><td>65 kDa</td><td>0</td><td>0</td><td>0</td><td>0</td><td>0</td><td>0</td><td>0</td><td>0</td><td>0</td><td>0</td></tr>
<tr><td>| Symbols: PIP2A, PIP2, PIP2;1 | plasma membrane intrinsic protein 2A | chr3:19803906-19805454 REVERSE LENGTH=287</td><td>AT3G53420.1 (+1)</td><td>30 kDa</td><td>4</td><td>4</td><td>0</td><td>1</td><td>0</td><td>1</td><td>2</td><td>1</td><td>0</td><td>0</td></tr>
<tr><td>| Symbols: | Ribosomal protein S10p/S20e family protein | chr3:16471606-16472312 REVERSE LENGTH=124</td><td>AT3G45030.1 (+2)</td><td>14 kDa</td><td>2</td><td>1</td><td>1</td><td>1</td><td>1</td><td>2</td><td>1</td><td>1</td><td>1</td><td>1</td></tr>
<tr><td>| Symbols: | Ribosomal protein S10p/S20e family protein | chr3:17453671-17454437 REVERSE LENGTH=122</td><td>AT3G47370.1 (+2)</td><td>14 kDa</td><td>0</td><td>0</td><td>0</td><td>0</td><td>0</td><td>0</td><td>0</td><td>0</td><td>0</td><td>0</td></tr>
<tr><td>| Symbols: | Coatomer, beta subunit | chr4:15269460-15272693 FORWARD LENGTH=948</td><td>AT4G31490.1</td><td>106 kDa</td><td>5</td><td>2</td><td>0</td><td>1</td><td>0</td><td>4</td><td>0</td><td>0</td><td>0</td><td>0</td></tr>
<tr><td>| Symbols: | Coatomer, beta subunit | chr4:15264145-15267384 FORWARD LENGTH=948</td><td>AT4G31480.1 (+1)</td><td>106 kDa</td><td>5</td><td>2</td><td>0</td><td>1</td><td>0</td><td>3</td><td>0</td><td>0</td><td>0</td><td>0</td></tr>
<tr><td>| Symbols: MLK3 | Protein kinase family protein | chr2:10985118-10988652 REVERSE LENGTH=673</td><td>AT2G25760.1</td><td>76 kDa</td><td>2</td><td>3</td><td>0</td><td>1</td><td>0</td><td>1</td><td>5</td><td>4</td><td>4</td><td>4</td></tr>
<tr><td>| Symbols: RPT3 | regulatory particle triple-A ATPase 3 | chr5:23569155-23571116 FORWARD LENGTH=408</td><td>AT5G58290.1</td><td>46 kDa</td><td>3</td><td>3</td><td>1</td><td>1</td><td>1</td><td>2</td><td>1</td><td>1</td><td>0</td><td>0</td></tr>
<tr><td>| Symbols: CPN60B, LEN1 | chaperonin 60 beta | chr1:20715717-20718673 REVERSE LENGTH=600</td><td>AT1G55490.1 (+1)</td><td>64 kDa</td><td>2</td><td>4</td><td>0</td><td>3</td><td>0</td><td>2</td><td>2</td><td>0</td><td>1</td><td>0</td></tr>
<tr><td>| Symbols: | TCP-1/cpn60 chaperonin family protein | chr5:22874058-22876966 FORWARD LENGTH=597</td><td>AT5G56500.1 (+1)</td><td>63 kDa</td><td>0</td><td>2</td><td>0</td><td>0</td><td>0</td><td>0</td><td>0</td><td>0</td><td>0</td><td>0</td></tr>
<tr><td>| Symbols: ATARCA, RACK1A_AT, RACK1A | Transducin/WD40 repeat-like superfamily protein | chr1:6222325-6223901 FORWARD LENGTH=327</td><td>AT1G18080.1</td><td>36 kDa</td><td>1</td><td>1</td><td>0</td><td>2</td><td>0</td><td>3</td><td>1</td><td>0</td><td>0</td><td>0</td></tr>
<tr><td>| Symbols: RACK1B_AT | receptor for activated C kinase 1B | chr1:17981977-17983268 REVERSE LENGTH=326</td><td>AT1G48630.1</td><td>36 kDa</td><td>3</td><td>0</td><td>0</td><td>0</td><td>0</td><td>2</td><td>0</td><td>1</td><td>0</td><td>0</td></tr>
<tr><td>| Symbols: RACK1C_AT | receptor for activated C kinase 1C | chr3:6211109-6212371 REVERSE LENGTH=326</td><td>AT3G18130.1</td><td>36 kDa</td><td>0</td><td>0</td><td>0</td><td>0</td><td>0</td><td>0</td><td>0</td><td>0</td><td>0</td><td>0</td></tr>
<tr><td>| Symbols: AHA2, PMA2, HA2 | H(+)-ATPase 2 | chr4:14770820-14775920 REVERSE LENGTH=948</td><td>AT4G30190.1 (+1)</td><td>104 kDa</td><td>4</td><td>3</td><td>0</td><td>1</td><td>0</td><td>2</td><td>0</td><td>0</td><td>0</td><td>0</td></tr>
<tr><td>| Symbols: AHA1, PMA, OST2, HA1 | H(+)-ATPase 1 | chr2:8221858-8227268 FORWARD LENGTH=949</td><td>AT2G18960.1</td><td>104 kDa</td><td>4</td><td>2</td><td>0</td><td>1</td><td>0</td><td>2</td><td>0</td><td>0</td><td>0</td><td>0</td></tr>
<tr><td>| Symbols: AHA7, HA7 | H(+)-ATPase 7 | chr3:22298763-22303509 FORWARD LENGTH=961</td><td>AT3G60330.1 (+1)</td><td>106 kDa</td><td>1</td><td>0</td><td>0</td><td>0</td><td>0</td><td>0</td><td>0</td><td>0</td><td>0</td><td>0</td></tr>
<tr><td>| Symbols: AHA4, HA4 | H(+)-ATPase 4 | chr3:17693015-17697801 FORWARD LENGTH=960</td><td>AT3G47950.1</td><td>106 kDa</td><td>0</td><td>0</td><td>0</td><td>0</td><td>0</td><td>0</td><td>0</td><td>0</td><td>0</td><td>0</td></tr>
<tr><td>| Symbols: AHA3, ATAHA3, HA3 | H(+)-ATPase 3 | chr5:23231208-23236381 REVERSE LENGTH=949</td><td>AT5G57350.1 (+1)</td><td>104 kDa</td><td>0</td><td>0</td><td>0</td><td>0</td><td>0</td><td>0</td><td>0</td><td>0</td><td>0</td><td>0</td></tr>
<tr><td>| Symbols: AHA10 | autoinhibited H(+)-ATPase isoform 10 | chr1:5904058-5908898 FORWARD LENGTH=947</td><td>AT1G17260.1</td><td>105 kDa</td><td>0</td><td>0</td><td>0</td><td>0</td><td>0</td><td>0</td><td>0</td><td>0</td><td>0</td><td>0</td></tr>
<tr><td>| Symbols: | ATPase, F1 complex, alpha subunit protein | chr2:3361474-3364028 FORWARD LENGTH=777</td><td>AT2G07698.1</td><td>86 kDa</td><td>2</td><td>2</td><td>1</td><td>3</td><td>1</td><td>4</td><td>1</td><td>0</td><td>0</td><td>0</td></tr>
<tr><td>| Symbols: LWD2 | Transducin/WD40 repeat-like superfamily protein | chr3:9793276-9794316 FORWARD LENGTH=346</td><td>AT3G26640.1</td><td>39 kDa</td><td>5</td><td>5</td><td>3</td><td>5</td><td>3</td><td>5</td><td>6</td><td>4</td><td>3</td><td>3</td></tr>
<tr><td>| Symbols: | ATPase, V1 complex, subunit B protein | chr1:7016971-7020290 FORWARD LENGTH=487</td><td>AT1G20260.1 (+2)</td><td>54 kDa</td><td>3</td><td>3</td><td>0</td><td>2</td><td>0</td><td>2</td><td>1</td><td>2</td><td>0</td><td>0</td></tr>
<tr><td>| Symbols: ATGSTF8, ATGSTF5, GST6, GSTF8 | glutathione S-transferase phi 8 | chr2:19558213-19559266 FORWARD LENGTH=263</td><td>AT2G47730.1</td><td>29 kDa</td><td>3</td><td>2</td><td>0</td><td>0</td><td>0</td><td>4</td><td>0</td><td>0</td><td>0</td><td>0</td></tr>
<tr><td>| Symbols: ATPC1 | ATPase, F1 complex, gamma subunit protein | chr4:2350761-2351882 REVERSE LENGTH=373</td><td>AT4G04640.1</td><td>41 kDa</td><td>5</td><td>3</td><td>0</td><td>2</td><td>1</td><td>2</td><td>1</td><td>2</td><td>1</td><td>0</td></tr>
<tr><td>| Symbols: FBA2 | fructose-bisphosphate aldolase 2 | chr4:18163714-18165659 REVERSE LENGTH=398</td><td>AT4G38970.1</td><td>43 kDa</td><td>2</td><td>2</td><td>0</td><td>3</td><td>1</td><td>3</td><td>2</td><td>1</td><td>0</td><td>0</td></tr>
<tr><td>| Symbols: RPL16A | ribosomal protein large subunit 16A | chr2:17791794-17792946 FORWARD LENGTH=182</td><td>AT2G42740.1 (+4)</td><td>21 kDa</td><td>2</td><td>2</td><td>1</td><td>1</td><td>1</td><td>1</td><td>1</td><td>2</td><td>2</td><td>1</td></tr>
<tr><td>| Symbols: | Transketolase | chr3:22454004-22456824 FORWARD LENGTH=741</td><td>AT3G60750.1 (+1)</td><td>80 kDa</td><td>3</td><td>2</td><td>0</td><td>0</td><td>0</td><td>7</td><td>1</td><td>0</td><td>0</td><td>0</td></tr>
<tr><td>| Symbols: | binding | chr5:19317899-19327014 FORWARD LENGTH=1606</td><td>AT5G47690.2 (+1)</td><td>181 kDa</td><td>6</td><td>0</td><td>0</td><td>2</td><td>0</td><td>6</td><td>0</td><td>0</td><td>0</td><td>0</td></tr>
<tr><td>| Symbols: SAC52, RPL10, RPL10A | Ribosomal protein L16p/L10e family protein | chr1:4888270-4889408 FORWARD LENGTH=220</td><td>AT1G14320.1</td><td>25 kDa</td><td>1</td><td>2</td><td>1</td><td>1</td><td>0</td><td>1</td><td>2</td><td>2</td><td>1</td><td>1</td></tr>
<tr><td>| Symbols: CAC3 | acetyl Co-enzyme a carboxylase carboxyltransferase alpha subunit | chr2:15917612-15920749 FORWARD LENGTH=769</td><td>AT2G38040.1 (+1)</td><td>85 kDa</td><td>3</td><td>1</td><td>0</td><td>0</td><td>0</td><td>4</td><td>0</td><td>0</td><td>0</td><td>0</td></tr>
<tr><td>| Symbols: RAN-1, RAN1, ATRAN1 | RAS-related nuclear protein-1 | chr5:6760364-6761747 FORWARD LENGTH=221</td><td>AT5G20010.1 (+2)</td><td>25 kDa</td><td>2</td><td>3</td><td>0</td><td>2</td><td>0</td><td>5</td><td>1</td><td>0</td><td>0</td><td>0</td></tr>
<tr><td>| Symbols: | Ribosomal protein S4 | chr5:4935124-4936334 REVERSE LENGTH=198</td><td>AT5G15200.1</td><td>23 kDa</td><td>2</td><td>2</td><td>1</td><td>2</td><td>1</td><td>2</td><td>1</td><td>0</td><td>0</td><td>0</td></tr>
<tr><td>| Symbols: | Ribosomal protein S26e family protein | chr2:16918506-16919623 FORWARD LENGTH=133</td><td>AT2G40510.1 (+2)</td><td>15 kDa</td><td>2</td><td>2</td><td>0</td><td>2</td><td>0</td><td>2</td><td>2</td><td>2</td><td>1</td><td>0</td></tr>
<tr><td>| Symbols: EMB2296 | Ribosomal protein L2 family | chr2:7837151-7838160 FORWARD LENGTH=258</td><td>AT2G18020.1</td><td>28 kDa</td><td>2</td><td>1</td><td>1</td><td>1</td><td>1</td><td>1</td><td>1</td><td>1</td><td>1</td><td>0</td></tr>
<tr><td>| Symbols: | Ribosomal protein L2 family | chr4:17097613-17098656 FORWARD LENGTH=258</td><td>AT4G36130.1</td><td>28 kDa</td><td>2</td><td>1</td><td>0</td><td>1</td><td>0</td><td>0</td><td>0</td><td>1</td><td>0</td><td>0</td></tr>
</table>

| Symbols: | Histone superfamily protein | chr1:2369212-2369523 FORWARD LENGTH=103	AT1G07660.1 (+8)	11 kDa	1	1	1	3	1	3	1	1	1	1
| Symbols: | oxidoreductases, acting on the aldehyde or oxo group of donors, NAD or NADP as acceptor;copper ion binding | chr2:8613168-8615649 FORWARD LENGTH=401	AT2G19940.1 (+1)	44 kDa	5	1	0	1	0	3	1	0	0	0
| Symbols: ATRPS5B, RPS5B | ribosomal protein 5B | chr2:15647883-15649042 REVERSE LENGTH=207	AT2G37270.1 (+3)	23 kDa	3	1	0	0	0	0	2	2	2	1
| Symbols: | Thioredoxin superfamily protein | chr3:3672189-3673937 FORWARD LENGTH=266	AT3G11630.1	29 kDa	0	1	0	0	0	2	1	2	0	0
| Symbols: PIP1B, TMP-A, ATHH2, PIP1;2 | plasma membrane intrinsic protein 1B | chr2:18910450-18911703 FORWARD LENGTH=286	AT2G45960.1 (+2)	31 kDa	1	1	0	2	1	2	1	1	0	0
| Symbols: RPL27A, RPL27AB | Ribosomal protein L18e/L15 superfamily protein | chr1:8263007-8263447 FORWARD LENGTH=146	AT1G23290.1 (+1)	16 kDa	1	1	0	1	0	1	2	1	1	0
| Symbols: | Ribosomal protein L18ae/LX family protein | chr2:14532916-14534161 REVERSE LENGTH=178	AT2G34480.1	21 kDa	2	1	0	2	0	1	1	0	1	0
| Symbols: | Ribosomal protein L18ae/LX family protein | chr3:4910773-4911933 FORWARD LENGTH=178	AT3G14600.1	21 kDa	0	0	0	1	0	0	0	0	0	0
| Symbols: EMB2719, HAP15 | PAM domain (PCI/PINT associated module) protein | chr1:7001409-7004154 REVERSE LENGTH=488	AT1G20200.1	56 kDa	3	3	0	1	0	3	0	0	0	0
| Symbols: | PAM domain (PCI/PINT associated module) protein | chr1:28524623-28526718 REVERSE LENGTH=487	AT1G75990.1	56 kDa	3	0	0	1	0	3	0	0	0	0
| Symbols: APS1 | ATP sulfurylase 1 | chr3:8112837-8114734 FORWARD LENGTH=463	AT3G22890.1	51 kDa	3	1	0	2	0	3	0	0	0	0
| Symbols: APS2, ASA1 | Pseudouridine synthase/archaeosine transglycosylase-like family protein | chr1:6914835-6916657 REVERSE LENGTH=476	AT1G19920.1	54 kDa	0	0	0	0	0	0	0	0	0	0
| Symbols: APS3 | Pseudouridine synthase/archaeosine transglycosylase-like family protein | chr4:8413443-8415311 REVERSE LENGTH=465	AT4G14680.1	52 kDa	0	0	0	0	0	0	0	0	0	0
| Symbols: | Tetratricopeptide repeat (TPR)-like superfamily protein | chr3:22565440-22566678 REVERSE LENGTH=412	AT3G60980.1	47 kDa	0	0	0	0	0	0	5	5	0	0
| Symbols: SPA2 | SPA1-related 2 | chr4:6772163-6776675 FORWARD LENGTH=1036	AT4G11110.1	115 kDa	5	6	0	0	0	0	0	0	0	0
| Symbols: PORB | protochlorophyllide oxidoreductase B | chr4:13725648-13727107 FORWARD LENGTH=401	AT4G27440.1 (+1)	43 kDa	3	2	0	1	0	1	1	0	0	0
| Symbols: ATPE | ATP synthase epsilon chain | chrC:52265-52663 REVERSE LENGTH=132	ATCG00470.1	14 kDa	2	2	0	1	0	1	1	2	0	0
| Symbols: RPL14 | ribosomal protein L14 | chrC:80696-81064 REVERSE LENGTH=122	ATCG00780.1	14 kDa	2	2	0	1	0	2	1	2	0	0
| Symbols: | C2 calcium/lipid-binding plant phosphoribosyltransferase family protein | chr5:2127200-2129584 REVERSE LENGTH=794	AT5G06850.1	91 kDa	0	0	0	2	0	5	0	0	0	0
| Symbols: FDH | formate dehydrogenase | chr5:4777043-4779190 FORWARD LENGTH=384	AT5G14780.1	42 kDa	2	1	0	1	0	3	0	1	0	0
| Symbols: TOC1, APRR1, PRR1, AtTOC1 | CCT motif -containing response regulator protein | chr5:24675540-24678176 FORWARD LENGTH=618	AT5G61380.1	69 kDa	2	2	2	0	3	0	1	0	1	1
| Symbols: ELF4-L2 | ELF4-like 2 | chr1:27344988-27345347 FORWARD LENGTH=119	AT1G72630.1	13 kDa	0	1	0	1	0	1	2	2	2	1
| Symbols: STV1, RPL24B, RPL24 | Ribosomal protein L24e family protein | chr3:19660749-19661912 REVERSE LENGTH=163	AT3G53020.1	19 kDa	2	2	0	1	0	1	2	2	0	0
| Symbols: | glycine-rich protein | chr4:11944050-11945565 REVERSE LENGTH=356	AT4G22740.1 (+1)	39 kDa	2	2	0	0	0	2	1	0	0	0
| Symbols: | Ribosomal L28e protein family | chr2:8511752-8512995 FORWARD LENGTH=143	AT2G19730.1 (+2)	16 kDa	1	1	0	0	0	2	0	0	2	0
| Symbols: | Ribosomal protein S11 family protein | chr2:15169925-15171159 FORWARD LENGTH=150	AT2G36160.1	16 kDa	2	2	1	3	1	3	0	0	0	0
| Symbols: | Ribosomal protein S11 family protein | chr3:3623757-3624866 REVERSE LENGTH=150	AT3G11510.1	16 kDa	1	2	1	2	1	2	0	0	0	0
| Symbols: | Coatomer, beta' subunit | chr3:5411699-5418313 REVERSE LENGTH=909	AT3G15980.1 (+3)	102 kDa	3	1	0	0	0	4	0	0	0	0
| Symbols: | structural molecules | chr1:30084522-30091949 FORWARD LENGTH=1135	AT1G79990.1 (+2)	129 kDa	2	0	0	0	0	2	0	0	0	0
| Symbols: XIK, ATXIK, XI-17 | Myosin family protein with Dil domain | chr5:6927064-6936825 REVERSE LENGTH=1545	AT5G20490.1 (+1)	175 kDa	1	2	0	0	0	0	0	0	0	0
| Symbols: MYA1, ATMYA1, XI-1 | myosin 1 | chr1:6039453-6049309 FORWARD LENGTH=1520	AT1G17580.1	173 kDa	1	1	0	0	0	0	0	0	0	0
| Symbols: XIC, ATXIC | Myosin family protein with Dil domain | chr1:2779963-2788325 FORWARD LENGTH=1538	AT1G08730.1	175 kDa	0	1	0	0	0	0	0	0	0	0
| Symbols: HSP81-2, ERD8, HSP90.2, AtHsp90.2 | heat shock protein 81-2 | chr5:22686923-22689433 FORWARD LENGTH=699	AT5G56030.1 (+1)	80 kDa	3	2	0	1	0	1	1	0	0	0
| Symbols: PIP3, PIP3A, PIP2;7, SIMIP | plasma membrane intrinsic protein 3 | chr4:16708672-16709958 FORWARD LENGTH=280	AT4G35100.1 (+1)	30 kDa	3	3	1	2	1	0	2	0	0	0
| Symbols: RPS4 | chloroplast ribosomal protein S4 | chrC:45223-45828 REVERSE LENGTH=201	ATCG00380.1	23 kDa	1	1	0	0	0	0	2	2	0	0
| Symbols: LOS2, ENO2 | Enolase | chr2:15321081-15323786 REVERSE LENGTH=444	AT2G36530.1	48 kDa	1	1	0	1	0	2	1	0	1	0
| Symbols: | ATPase, V0/A0 complex, subunit C/D | chr3:10778025-10780350 FORWARD LENGTH=351	AT3G28715.1	41 kDa	2	1	0	1	0	1	1	0	0	0
| Symbols: | ATPase, V0/A0 complex, subunit C/D | chr3:10773144-10775594 REVERSE LENGTH=351	AT3G28710.1	41 kDa	1	1	0	2	0	2	0	0	0	0
| Symbols: cpHsc70-1 | chloroplast heat shock protein 70-1 | chr4:12590094-12593437 FORWARD LENGTH=718	AT4G24280.1	77 kDa	0	0	0	2	0	5	1	0	0	0
| Symbols: CPHSC70-2EAT SHOCK PROTEIN 70-2, HSC70-7, cpHsc70-2 | chloroplast heat shock protein 70-2 | chr5:20303470-20306295 FORWARD LENGTH=718	AT5G49910.1	77 kDa	0	0	0	3	0	4	1	0	0	0
| Symbols: LUH | LEUNIG_homolog | chr2:13867235-13871844 FORWARD LENGTH=787	AT2G32700.1 (+6)	86 kDa	0	0	0	1	0	3	0	0	0	0
| Symbols: emb1138 | DEAD box RNA helicase (RH3) | chr5:9285540-9288871 REVERSE LENGTH=747	AT5G26742.1 (+1)	81 kDa	3	2	0	1	0	0	0	0	0	0
| Symbols: RPS11 | ribosomal protein S11 | chrC:78960-79376 REVERSE LENGTH=138	ATCG00750.1	15 kDa	2	2	0	1	0	1	2	2	0	0
| Symbols: | Ribosomal protein S4 (RPS4A) family protein | chr2:7546598-7548138 FORWARD LENGTH=261	AT2G17360.1 (+3)	30 kDa	2	0	1	1	0	1	1	0	0	0
| Symbols: PFL, RPS18A, PFL1 | Ribosomal protein S13/S18 family | chr1:8067990-8069163 FORWARD LENGTH=152	AT1G22780.1 (+2)	18 kDa	0	1	1	2	0	1	0	1	1	0
| Symbols: P40, AP40, RP40, RPSAA | 40s ribosomal protein SA | chr1:27243148-27244842 REVERSE LENGTH=298	AT1G72370.1 (+1)	32 kDa	1	3	0	2	0	1	0	0	0	0
| Symbols: RPSAb | 40s ribosomal protein SA B | chr3:1309544-1310846 REVERSE LENGTH=280	AT3G04770.2	31 kDa	0	0	0	1	0	0	0	0	0	0
| Symbols: CLPC, ATHSP93-V, HSP93-V, DCA1, CLPC1 | CLPC homologue 1 | chr5:20715710-20719800 REVERSE LENGTH=929	AT5G50920.1	?	4	2	0	0	0	1	0	0	0	0
| Symbols: ATCLPC, ATHSP93-III, HSP93-III | Clp ATPase | chr3:18122363-18126008 REVERSE LENGTH=952	AT3G48870.1 (+1)	106 kDa	3	2	0	0	0	1	0	0	0	0
| Symbols: SPA4 | SPA1-related 4 | chr1:19783748-19786690 FORWARD LENGTH=794	AT1G53090.1 (+1)	89 kDa	3	6	0	0	0	0	0	0	0	0
| Symbols: | AAA-type ATPase family protein | chr2:8692736-8694837 FORWARD LENGTH=443	AT2G20140.1 (+1)	49 kDa	5	3	0	0	0	0	1	0	0	0
| Symbols: RPT6A, ATSUG1 | regulatory particle triple-A ATPase 6A | chr5:6752144-6754918 FORWARD LENGTH=419	AT5G19990.1 (+1)	47 kDa	1	1	0	1	0	2	1	1	0	0
| Symbols: | Lactate/malate dehydrogenase family protein | chr1:1189418-1191267 REVERSE LENGTH=332	AT1G04410.1	36 kDa	0	2	0	1	0	1	0	0	0	0
| Symbols: | Aldolase superfamily protein | chr3:19627383-19628874 REVERSE LENGTH=358	AT3G52930.1	39 kDa	0	1	0	1	0	3	1	0	1	0
| Symbols: | Aldolase superfamily protein | chr5:963389-964982 REVERSE LENGTH=359	AT5G03690.2	39 kDa	0	0	0	1	0	0	0	0	0	0
| Symbols: | Aldolase superfamily protein | chr4:13391566-13392937 FORWARD LENGTH=358	AT4G26530.1 (+1)	38 kDa	0	0	0	0	0	1	0	0	0	0
| Symbols: CHLI1, CH42, CH-42, CHL11, CHLI-1 | P-loop containing nucleoside triphosphate hydrolases superfamily protein | chr4:10201897-10203361 REVERSE LENGTH=424	AT4G18480.1	46 kDa	2	1	0	0	0	2	0	0	0	0
| Symbols: CHLI2, CHL I2, CHLI-2 | magnesium chelatase i2 | chr5:18628095-18629565 FORWARD LENGTH=418	AT5G45930.1	46 kDa	0	0	0	0	0	0	0	0	0	0
| Symbols: | transducin family protein / WD-40 repeat family protein | chr3:23431009-23437241 REVERSE LENGTH=1104	AT3G63460.1 (+2)	120 kDa	1	1	0	1	0	2	0	0	0	0
| Symbols: | vacuolar ATP synthase subunit H family protein | chr3:14228846-14232228 REVERSE LENGTH=441	AT3G42050.1	50 kDa	2	1	0	1	0	1	0	0	0	0
| Symbols: RPL18 | ribosomal protein L18 | chr3:1621511-1622775 FORWARD LENGTH=187	AT3G05590.1 (+1)	21 kDa	2	1	1	1	0	2	1	1	0	0
| Symbols: RPT5A, ATS6A.2 | regulatory particle triple-A ATPase 5A | chr3:1603540-1605993 FORWARD LENGTH=424	AT3G05530.1	47 kDa	3	2	0	0	0	1	2	2	0	0
| Symbols: AGT, AGT1, SGAT | alanine:glyoxylate aminotransferase | chr2:5539417-5540902 REVERSE LENGTH=401	AT2G13360.1 (+1)	44 kDa	1	1	1	0	1	2	0	0	0	0
| Symbols: | Ribosomal protein L22p/L17e family protein | chr1:9515230-9516725 FORWARD LENGTH=176	AT1G27400.1 (+1)	20 kDa	0	1	2	0	1	2	0	0	0	0
| Symbols: VHA-A | vacuolar ATP synthase subunit A | chr1:29660463-29664575 FORWARD LENGTH=623	AT1G78900.1 (+1)	69 kDa	1	0	0	0	0	2	0	0	0	0
| Symbols: RPT1A | regulatory particle triple-A 1A | chr1:20065921-20068324 REVERSE LENGTH=426	AT1G53750.1	48 kDa	2	1	0	1	0	1	0	0	0	0
| Symbols: ATJ2, J2 | DNAJ homologue 2 | chr5:7303798-7305668 REVERSE LENGTH=419	AT5G22060.1	46 kDa	2	2	0	0	0	1	0	0	0	0
| Symbols: ATJ3, ATJ | DNAJ homologue 3 | chr3:15869115-15871059 REVERSE LENGTH=420	AT3G44110.1 (+1)	46 kDa	2	2	0	0	0	2	0	0	0	0
| Symbols: RPS1, ARRPS1 | ribosomal protein S1 | chr5:11619262-11621223 REVERSE LENGTH=416	AT5G30510.1	45 kDa	1	0	0	3	0	4	0	0	0	0
| Symbols: ACCD | acetyl-CoA carboxylase carboxyl transferase subunit beta | chrC:57075-58541 FORWARD LENGTH=488	ATCG00500.1	56 kDa	2	1	0	0	0	1	0	0	0	0
| Symbols: RPS8 | ribosomal protein S8 | chrC:80068-80472 REVERSE LENGTH=134	ATCG00770.1	15 kDa	1	2	0	1	0	1	1	1	0	0
| Symbols: SSR16 | small subunit ribosomal protein 16 | chr4:16535084-16536092 REVERSE LENGTH=113	AT4G34620.1	13 kDa	1	1	0	1	0	1	1	1	2	0
| Symbols: | Sucrose-6F-phosphate phosphohydrolase family protein | chr2:15053952-15055776 FORWARD LENGTH=422	AT2G35840.1 (+2)	48 kDa	1	0	0	0	0	2	0	0	0	0
| Symbols: | GTP binding Elongation factor Tu family protein | chr4:1295751-1298354 REVERSE LENGTH=454	AT4G02930.1	49 kDa	3	0	0	1	0	3	0	0	0	0
| Symbols: ECT2 | evolutionarily conserved C-terminal region 2 | chr3:4385274-4388220 REVERSE LENGTH=667	AT3G13460.1 (+3)	72 kDa	3	2	0	0	0	0	0	0	0	0
| Symbols: | Glycine cleavage T-protein family | chr1:4001801-4003245 FORWARD LENGTH=408	AT1G11860.1 (+2)	44 kDa	1	1	0	1	0	1	2	0	0	0
| Symbols: NOP56 | homolog of nucleolar protein NOP56 | chr1:20984544-20986893 REVERSE LENGTH=522	AT1G56110.1	59 kDa	1	0	0	0	0	2	0	0	0	0
| Symbols: RPS15A | ribosomal protein S15A | chr1:2408413-2409065 REVERSE LENGTH=130	AT1G07770.1 (+2)	15 kDa	1	1	1	0	1	3	0	0	0	0
| Symbols: | Ribosomal protein S8 family protein | chr2:16517588-16518265 REVERSE LENGTH=136	AT2G39590.1	15 kDa	0	0	0	0	0	0	0	0	0	0
| Symbols: RPN1A, ATRPN1A | 26S proteasome regulatory subunit S2 1A | chr2:8859211-8864699 FORWARD LENGTH=891	AT2G20580.1	?	2	1	0	0	0	0	0	0	0	0
| Symbols: PAC1 | 20S proteasome alpha subunit C1 | chr3:7792819-7793571 REVERSE LENGTH=250	AT3G22110.1	27 kDa	0	2	0	0	0	1	1	1	0	0
| Symbols: ATBBC1, BBC1, RSU2 | breast basic conserved 1 | chr3:18166971-18168047 REVERSE LENGTH=206	AT3G49010.1 (+3)	24 kDa	0	0	0	1	0	2	1	0	0	0
| Symbols: | Tetratricopeptide repeat (TPR)-like superfamily protein | chr3:22535729-22536940 FORWARD LENGTH=403	AT3G60960.1	46 kDa	0	0	0	0	0	0	1	2	0	0
| Symbols: CPN60A, CH-CPN60A, SLP | chaperonin-60alpha | chr2:11926603-11929184 REVERSE LENGTH=586	AT2G28000.1	62 kDa	2	1	0	0	0	0	0	0	0	0
| Symbols: ATTIP49A, RIN1 | P-loop containing nucleoside triphosphate hydrolases superfamily protein | chr5:7391026-7394071 REVERSE LENGTH=458	AT5G22330.1	50 kDa	0	2	0	1	0	1	0	0	0	0

| Symbols: RPL16 | ribosomal protein L16 | chrC:81189-82652 REVERSE LENGTH=135	ATCG00790.1	15 kDa	1	0	0	1	0	2	0	0	0	0
| Symbols: PUB12, AtPUB12 | PLANT U-BOX 12 | chr2:12367001-12370608 REVERSE LENGTH=962	AT2G28830.1	107 kDa	1	0	0	1	0	1	0	0	0	0
| Symbols: | NAD(P)-binding Rossmann-fold superfamily protein | chr3:11193767-11194948 REVERSE LENGTH=298	AT3G29250.1	32 kDa	0	0	0	1	0	3	0	0	0	0
| Symbols: ILA | ILITYHIA | chr1:24065232-24081908 REVERSE LENGTH=2610	AT1G64790.1 (+1)	285 kDa	3	0	0	0	0	0	0	0	0	0
| Symbols: APUM5, PUM5 | pumilio 5 | chr3:7059098-7062660 REVERSE LENGTH=961	AT3G20250.1	107 kDa	3	1	0	1	0	0	0	0	0	0
| Symbols: ATTPS7, TPS7, ATTPSA | trehalose-phosphatase/synthase 7 | chr1:1955413-1958153 FORWARD LENGTH=851	AT1G06410.1	97 kDa	1	0	0	0	0	3	0	0	0	0
| Symbols: | transcription regulators | chr1:373335-386682 FORWARD LENGTH=2431	AT1G02080.1 (+1)	270 kDa	2	1	0	0	0	0	0	0	0	0
| Symbols: ATGSTF9, GLUTTR, ATGSTF7, GSTF9 | glutathione S-transferase PHI 9 | chr2:13139132-13140057 FORWARD LENGTH=215	AT2G30860.1 (+1)	24 kDa	0	2	0	1	0	0	0	0	0	0
| Symbols: | Ribosomal protein L35Ae family protein | chr1:27928415-27929466 REVERSE LENGTH=112	AT1G74270.1	13 kDa	1	0	0	2	0	1	0	0	0	0
| Symbols: ATU2AF35B, U2AF35B | Zinc finger C-x8-C-x5-C-x3-H type family protein | chr5:17170445-17171296 REVERSE LENGTH=283	AT5G42820.1 (+1)	33 kDa	2	0	0	1	0	1	0	0	0	0
| Symbols: ATU2AF35A | U2 snRNP auxiliary factor small subunit, putative | chr1:9615302-9616042 FORWARD LENGTH=246	AT1G27650.2	29 kDa	0	0	0	1	0	1	0	0	0	0
| Symbols: | Zinc finger C-x8-C-x5-C-x3-H type family protein | chr3:16328221-16328448 REVERSE LENGTH=75	AT3G44785.1	9 kDa	0	0	0	0	0	0	0	0	0	0
| Symbols: PIL6, PIF5 | phytochrome interacting factor 3-like 6 | chr3:21828189-21829895 REVERSE LENGTH=442	AT3G59060.1 (+3)	49 kDa	0	0	1	0	0	0	2	0	0	0
| Symbols: PIF4, SRL2, AtPIF4 | phytochrome interacting factor 4 | chr2:17887003-17888823 FORWARD LENGTH=430	AT2G43010.1 (+1)	48 kDa	0	0	1	0	0	0	1	0	0	0
| Symbols: | Ribosomal protein L31e family protein | chr2:8513577-8514346 FORWARD LENGTH=119	AT2G19740.1 (+2)	14 kDa	0	0	0	2	0	2	0	0	0	0
| Symbols: | Ribosomal protein L31e family protein | chr5:22944003-22944767 REVERSE LENGTH=85	AT5G56710.2	10 kDa	0	0	0	1	0	1	0	0	0	0
| Symbols: | Coatomer epsilon subunit | chr1:10858546-10860173 REVERSE LENGTH=292	AT1G30630.1	33 kDa	0	0	0	2	0	2	0	0	0	0
| Symbols: SHM4 | serine hydroxymethyltransferase 4 | chr4:8048013-8050021 REVERSE LENGTH=471	AT4G13930.1	52 kDa	2	1	0	0	0	0	0	0	0	0
| Symbols: RPL22 | ribosomal protein L22 | chrC:83467-83949 REVERSE LENGTH=160	ATCG00810.1	19 kDa	0	0	0	1	0	2	0	0	0	0
| Symbols: PBE1 | 20S proteasome beta subunit E1 | chr1:4452641-4454663 FORWARD LENGTH=274	AT1G13060.1 (+2)	30 kDa	0	2	0	0	0	0	0	0	0	0
| Symbols: PSBB | photosystem II reaction center protein B | chrC:72371-73897 FORWARD LENGTH=508	ATCG00680.1	56 kDa	1	1	0	0	0	0	2	0	0	0
| Symbols: NRPB1, RPB1, RNA_POL_II_LSRNA_POL_II_LS, RNA_POL_II_LS | RNA polymerase II large subunit | chr4:16961115-16967892 REVERSE LENGTH=1839	AT4G35800.1	205 kDa	2	0	0	0	0	0	0	0	0	0
| Symbols: EMB2761 | threonyl-tRNA synthetase, putative / threonine--tRNA ligase, putative | chr2:1698466-1701271 REVERSE LENGTH=650	AT2G04842.1	75 kDa	0	0	0	0	0	2	0	0	0	0
| Symbols: ELF4-L4 | ELF4-like 4 | chr1:5997932-5998276 FORWARD LENGTH=114	AT1G17455.1 (+1)	13 kDa	0	0	0	0	0	0	2	2	1	0
| Symbols: | AAA-type ATPase family protein | chr1:17009220-17011607 FORWARD LENGTH=399	AT1G45000.1 (+2)	45 kDa	2	2	0	0	0	0	2	0	0	0
| Symbols: PHOT1, NPH1, JK224, RPT1 | phototropin 1 | chr3:16818557-16823960 FORWARD LENGTH=996	AT3G45780.1 (+1)	112 kDa	2	0	0	0	0	1	0	0	0	0
| Symbols: PHOT2, NPL1 | phototropin 2 | chr5:23524771-23529993 FORWARD LENGTH=915	AT5G58140.1 (+3)	102 kDa	0	0	0	0	0	0	0	0	0	0
| Symbols: ZFN1 | zinc finger protein 1 | chr3:614075-615916 FORWARD LENGTH=397	AT3G02830.1	44 kDa	2	0	0	0	0	0	0	0	0	0
| Symbols: | Ribosomal protein L35Ae family protein | chr1:15651585-15652427 REVERSE LENGTH=111	AT1G41880.1	13 kDa	0	0	0	2	0	1	0	0	0	0
| Symbols: SPA3 | SPA1-related 3 | chr3:5169327-5172480 REVERSE LENGTH=837	AT3G15354.1	93 kDa	1	2	0	0	0	0	0	0	0	0
| Symbols: PRP39 | Tetratricopeptide repeat (TPR)-like superfamily protein | chr1:1051803-1056550 FORWARD LENGTH=768	AT1G04080.1 (+1)	85 kDa	2	0	0	0	0	0	0	0	0	0
| Symbols: ABA1, LOS6, NPQ2, ATABA1, ZEP, IBS3, ATZEP | zeaxanthin epoxidase (ZEP) (ABA1) | chr5:26753745-26757090 REVERSE LENGTH=667	AT5G67030.1 (+1)	74 kDa	2	0	0	0	0	0	0	0	0	0

Percent Coverage

Identified Proteins (283)	Accession Number	Molecular Weight	AtELF3-1	AtELF3-2	SvELF3#2 rep1	SvELF3#2 rep2	SvELF3#3 rep1	SvELF3#3 rep2	BdELF3#2 rep1	BdELF3#2 rep2	BdELF3#3 rep1	BdELF3#3 rep2
| Symbols: PHYB, HY3, OOP1 | phytochrome B | chr2:8140079-8144151 FORWARD LENGTH=1172	AT2G18790.1	129 kDa	36.80%	37.40%	54.20%	42.20%	49.60%	45.00%	33.70%	32.90%	39.80%	37.30%
| Symbols: PHYD | phytochrome D | chr4:9195602-9199486 REVERSE LENGTH=1164	AT4G16250.1	129 kDa	10.10%	11.00%	11.90%	11.70%	14.70%	11.90%	11.10%	10.10%	13.60%	11.80%
| Symbols: HSC70-1, HSP70-1, AT-HSC70-1, HSC70 | heat shock cognate protein 70-1 | chr5:554055-556334 REVERSE LENGTH=651	AT5G02500.1	71 kDa	36.10%	28.40%	55.60%	51.00%	53.80%	58.70%	28.40%	28.10%	16.70%	22.60%
| Symbols: | Heat shock protein 70 (Hsp 70) family protein | chr3:2903434-2905632 REVERSE LENGTH=649	AT3G09440.1 (+1)	71 kDa	37.30%	33.60%	49.90%	44.40%	51.00%	47.80%	33.60%	31.70%	12.80%	24.50%
| Symbols: HSP70, ATHSP70 | heat shock protein 70 | chr3:3991487-3993689 REVERSE LENGTH=650	AT3G12580.1	71 kDa	27.80%	20.80%	31.20%	36.90%	37.50%	41.70%	22.60%	22.30%	8.77%	15.10%
| Symbols: | Heat shock protein 70 (Hsp 70) family protein | chr5:550296-552565 REVERSE LENGTH=653	AT5G02490.1	71 kDa	31.70%	24.50%	29.60%	28.20%	31.40%	39.20%	22.70%	20.80%	8.73%	15.00%
| Symbols: Hsp70b | heat shock protein 70B | chr1:5502386-5504326 REVERSE LENGTH=646	AT1G16030.1	71 kDa	0	0	0	0	0	0	0	0	0	0
| Symbols: BdELF3_HFC	ATMGcustom02	86 kDa	2.38%	0	1.00%	0.00%	1.00%	0.00%	41.70%	42.70%	35.30%	33.10%
| Symbols: PHYC | phytochrome C | chr5:14008049-14011619 FORWARD LENGTH=1111	AT5G35840.1	124 kDa	29.40%	28.90%	37.40%	38.70%	37.10%	42.10%	24.10%	22.00%	22.70%	26.80%
| Symbols: SvELF3_HFC	ATMGcustom01	87 kDa	2.38%	0	40.20%	48.00%	42.40%	54.40%	1.00%	1.00%	1.00%	2.38%
| Symbols: AtELF3_HFC	ATMGcustom03	84 kDa	35.80%	31.40%	0	0	0	0	0.00%	0	0	2.51%
| Symbols: TZP | zinc knuckle (CCHC-type) family protein | chr5:17527261-17530207 FORWARD LENGTH=831	AT5G43630.1	91 kDa	21.40%	17.00%	22.50%	32.50%	22.00%	36.10%	17.80%	19.30%	20.20%	21.70%
| Symbols: RBCL | ribulose-bisphosphate carboxylases | chrC:54958-56397 FORWARD LENGTH=479	ATCG00490.1	53 kDa	32.20%	30.10%	39.20%	34.20%	29.00%	46.10%	32.20%	32.20%	21.50%	22.80%
| Symbols: PHYE | phytochrome E | chr4:10042312-10045948 REVERSE LENGTH=1112	AT4G18130.1	123 kDa	16.00%	26.60%	18.30%	23.20%	18.50%	23.50%	7.46%	14.10%	19.20%	18.40%
| Symbols: TUB2 | tubulin beta chain 2 | chr5:25181560-25183501 FORWARD LENGTH=450	AT5G62690.1 (+1)	51 kDa	31.10%	27.10%	9.78%	23.10%	12.90%	28.90%	20.70%	16.00%	13.30%	11.10%
| Symbols: TUB5 | tubulin beta-5 chain | chr1:6938033-6940481 REVERSE LENGTH=449	AT1G20010.1	50 kDa	25.60%	20.90%	0	21.60%	0	27.60%	20.90%	10.20%	7.57%	5.35%
| Symbols: TUB6 | beta-6 tubulin | chr5:3961317-3962971 REVERSE LENGTH=449	AT5G12250.1	51 kDa	20.70%	18.30%	0	14.30%	0	20.30%	18.30%	10.20%	7.57%	5.35%
| Symbols: TUB9 | tubulin beta-9 chain | chr4:11182218-11183840 FORWARD LENGTH=444	AT4G20890.1	50 kDa	28.40%	24.30%	0	20.90%	7.21%	23.20%	15.80%	10.40%	7.66%	5.41%
| Symbols: TUB7 | tubulin beta-7 chain | chr2:12644258-12645932 REVERSE LENGTH=449	AT2G29550.1	51 kDa	27.40%	23.40%	0	16.70%	12.90%	26.30%	18.30%	16.00%	7.57%	5.35%
| Symbols: TUB4 | tubulin beta chain 4 | chr5:17859442-17860994 REVERSE LENGTH=444	AT5G44340.1	50 kDa	28.40%	24.30%	0	18.20%	7.21%	26.60%	18.50%	10.40%	7.66%	5.41%
| Symbols: TUB8 | tubulin beta 8 | chr5:8042962-8044528 FORWARD LENGTH=449	AT5G23860.1 (+1)	51 kDa	28.70%	24.70%	0	20.50%	12.90%	20.30%	18.30%	16.00%	7.57%	0
| Symbols: PCH1 | F-box family protein | chr2:7074940-7079181 FORWARD LENGTH=778	AT2G16365.1 (+2)	89 kDa	14.90%	14.90%	17.50%	25.10%	21.00%	28.70%	15.40%	16.60%	18.60%	18.60%
| Symbols: GAPC-2, GAPC2 | glyceraldehyde-3-phosphate dehydrogenase C2 | chr1:4608465-4610494 REVERSE LENGTH=338	AT1G13440.1	37 kDa	37.30%	31.70%	22.50%	58.60%	34.90%	52.70%	20.70%	12.70%	27.20%	10.40%
| Symbols: GAPC, GAPC-1, GAPC1 | glyceraldehyde-3-phosphate dehydrogenase C subunit 1 | chr3:1081077-1083131 FORWARD LENGTH=338	AT3G04120.1	37 kDa	37.30%	31.70%	22.50%	58.60%	34.90%	52.70%	20.70%	12.70%	27.20%	10.40%
| Symbols: ATPA | ATP synthase subunit alpha | chrC:9938-11461 REVERSE LENGTH=507	ATCG00120.1	55 kDa	31.60%	20.90%	2.37%	26.60%	0	35.70%	22.70%	20.90%	18.30%	9.86%
| Symbols: TUA4, TOR2 | tubulin alpha-4 chain | chr1:1356421-1358266 REVERSE LENGTH=450	AT1G04820.1 (+1)	50 kDa	20.20%	21.30%	18.70%	32.90%	21.30%	35.60%	8.44%	16.90%	6.44%	4.44%
| Symbols: TUA3 | tubulin alpha-3 | chr5:6682761-6684474 REVERSE LENGTH=450	AT5G19770.1 (+1)	50 kDa	11.10%	10.90%	11.10%	20.70%	9.11%	26.40%	4.00%	12.70%	2.00%	0
| Symbols: TUA6 | Tubulin/FtsZ family protein | chr4:8548753-8550319 REVERSE LENGTH=427	AT4G14960.1	47 kDa	21.30%	19.20%	16.40%	31.40%	19.20%	32.30%	8.90%	21.10%	6.79%	4.68%
| Symbols: ACT8 | actin 8 | chr1:18216539-18217947 FORWARD LENGTH=377	AT1G49240.1 (+1)	42 kDa	12.50%	12.50%	9.02%	18.30%	11.90%	15.10%	12.50%	12.50%	12.50%	12.50%
| Symbols: ACT7 | actin 7 | chr5:3052809-3054220 FORWARD LENGTH=377	AT5G09810.1	42 kDa	16.70%	16.70%	4.24%	22.50%	16.20%	22.30%	16.70%	12.50%	9.02%	4.24%
| Symbols: ACT1, AAc1 | actin 1 | chr2:15779761-15781241 FORWARD LENGTH=377	AT2G37620.1 (+2)	42 kDa	4.24%	0.00%	0	12.70%	2.92%	5.57%	0.00%	0	0	0
| Symbols: ACT4 | actin 4 | chr5:23950109-23951586 FORWARD LENGTH=377	AT5G59370.1 (+1)	42 kDa	0	0	0	13.30%	0	10.30%	4.77%	4.77%	4.77%	0
| Symbols: | Actin family protein | chr2:17578683-17580222 FORWARD LENGTH=329	AT2G42170.1	37 kDa	5.47%	5.47%	6.99%	5.47%	5.47%	5.47%	5.47%	5.47%	5.47%	0
| Symbols: | Actin-like ATPase superfamily protein | chr2:17560211-17561945 FORWARD LENGTH=378	AT2G42100.1	42 kDa	0	0	0	0	0	0	0	0	0	0
| Symbols: ATRAB8D, ATRABE1B, RABE1b | RAB GTPase homolog E1B | chr4:10990036-10991466 FORWARD LENGTH=476	AT4G20360.1	52 kDa	17.60%	14.10%	32.10%	34.70%	30.50%	40.50%	14.10%	11.60%	9.03%	9.03%
| Symbols: | Ribosomal protein S5 family protein | chr2:17460016-17461398 REVERSE LENGTH=285	AT2G41840.1	31 kDa	23.90%	28.40%	9.12%	14.00%	15.10%	20.00%	23.90%	23.90%	10.50%	5.26%
| Symbols: XW6 | Ribosomal protein S5 family protein | chr1:21689115-21690085 FORWARD LENGTH=284	AT1G58380.1 (+3)	31 kDa	27.80%	23.60%	13.40%	9.51%	23.90%	24.30%	14.10%	14.10%	5.28%	0.00%
| Symbols: | Ribosomal protein S5 family protein | chr3:21279824-21280887 REVERSE LENGTH=276	AT3G57490.1	30 kDa	12.70%	0	0	0	0	0	0	0	0	0
| Symbols: TIC | time for coffee | chr3:7913181-7918967 FORWARD LENGTH=1555	AT3G22380.2	165 kDa	5.47%	5.47%	4.18%	12.20%	2.96%	15.30%	4.05%	3.02%	1.29%	1.29%
| Symbols: ELF4 | Protein of unknown function (DUF1313) | chr2:16734545-16734880 REVERSE LENGTH=111	AT2G40080.1	12 kDa	49.50%	49.50%	42.30%	67.60%	60.40%	60.40%	49.50%	49.50%	67.60%	67.60%
| Symbols: GAPA, GAPA-1 | glyceraldehyde 3-phosphate dehydrogenase A subunit | chr3:9795226-9796848 FORWARD LENGTH=396	AT3G26650.1	42 kDa	36.60%	30.60%	11.40%	46.20%	14.90%	48.20%	20.20%	16.70%	14.10%	10.10%
| Symbols: GAPA-2 | glyceraldehyde 3-phosphate dehydrogenase A subunit 2 | chr1:4392634-4393850 REVERSE LENGTH=350	AT1G12900.3 (+1)	38 kDa	39.40%	34.60%	12.90%	39.40%	16.90%	39.40%	22.90%	14.30%	11.40%	4.29%
| Symbols: | Ribosomal protein L6 family | chr1:12010986-12012223 FORWARD LENGTH=194	AT1G33120.1 (+1)	22 kDa	24.70%	21.10%	34.00%	56.20%	34.00%	47.40%	25.80%	29.90%	29.90%	29.90%
| Symbols: | Ribosomal protein L6 family | chr4:6463201-6464458 REVERSE LENGTH=194	AT4G10450.1	22 kDa	4.12%	9.28%	0	14.40%	0	14.40%	0	9.28%	9.28%	9.28%
| Symbols: COP1, ATCOP1, DET340, FUS1, EMB168 | Transducin/WD40 repeat-like superfamily protein | chr2:13978000-13983282 FORWARD LENGTH=675	AT2G32950.1	76 kDa	15.70%	16.90%	17.20%	8.44%	6.22%	11.90%	1.78%	4.30%	11.30%	7.85%
| Symbols: IMPA-2 | importin alpha isoform 2 | chr4:9134450-9137134 REVERSE LENGTH=535	AT4G16143.1 (+1)	59 kDa	6.36%	6.36%	2.24%	23.40%	0.00%	26.40%	28.80%	26.70%	6.36%	10.70%
| Symbols: AT-IMP, ATKAP ALPHA, AIMP ALPHA, IMPA-1, IMPA1 | importin alpha isoform 1 | chr3:2120559-2123555 FORWARD LENGTH=532	AT3G06720.1 (+1)	59 kDa	0	0	0	5.08%	0	5.26%	5.08%	3.01%	0	0.00%
| Symbols: IMPA-6 | importin alpha isoform 6 | chr1:584397-587036 FORWARD LENGTH=538	AT1G02690.1 (+1)	59 kDa	0	0	0	0	0	0	0	0	0	0
| Symbols: IMPA-4 | importin alpha isoform 4 | chr1:2994506-2997833 FORWARD LENGTH=538	AT1G09270.1 (+2)	59 kDa	0	0	0	0	0	0	0	0	0	0
| Symbols: MTO3, SAMS3, MAT4 | S-adenosylmethionine synthetase family protein | chr3:5952484-5953665 REVERSE LENGTH=393	AT3G17390.1	43 kDa	39.20%	29.80%	9.92%	15.00%	0	24.40%	4.33%	0	0	0

| Symbols: MAT3 | methionine adenosyltransferase 3 | chr2:15479721-15480893 REVERSE LENGTH=390	AT2G36880.1 (+1)	42 kDa	20.50%	11.50%	10.00%	3.85%	0	10.00%	0	0	0	0
| Symbols: SAM-2, MAT2, SAM2, AtSAM2 | S-adenosylmethionine synthetase 2 | chr4:796298-797479 REVERSE LENGTH=393	AT4G01850.1 (+1)	43 kDa	23.70%	9.16%	0	3.82%	0	9.41%	0	0	0	0
| Symbols: SAM1, SAM-1, MAT1, AtSAM1 | S-adenosylmethionine synthetase 1 | chr1:519037-520218 FORWARD LENGTH=393	AT1G02500.1 (+1)	43 kDa	19.80%	5.34%	0	6.62%	0	12.20%	0	0	0	0
| Symbols: RCA | rubisco activase | chr2:16570951-16573345 REVERSE LENGTH=474	AT2G39730.1	52 kDa	23.80%	21.50%	10.80%	15.60%	13.30%	23.20%	21.50%	19.60%	12.00%	8.02%
| Symbols: PCL1, LUX | Homeodomain-like superfamily protein | chr3:17183248-17184219 FORWARD LENGTH=323	AT3G46640.1 (+1)	35 kDa	3.10%	3.10%	10.20%	18.90%	10.20%	22.90%	3.10%	3.10%	3.10%	10.50%
| Symbols: GS2, GLN2, ATGSL1 | glutamine synthetase 2 | chr5:13831220-13833239 FORWARD LENGTH=430	AT5G35630.1 (+2)	47 kDa	22.80%	17.70%	16.50%	33.00%	16.50%	22.60%	17.70%	14.00%	3.49%	3.49%
| Symbols: CAT3, SEN2, ATCAT3 | catalase 3 | chr1:7143142-7146193 FORWARD LENGTH=485	AT1G20620.5	56 kDa	37.30%	32.00%	4.74%	24.70%	2.89%	28.00%	23.70%	11.50%	2.27%	2.27%
| Symbols: | Clathrin, heavy chain | chr3:3482575-3491667 REVERSE LENGTH=1705	AT3G11130.1	193 kDa	13.30%	8.68%	0	1.94%	0	5.04%	2.17%	1.29%	0	0
| Symbols: | Clathrin, heavy chain | chr3:2587171-2595411 REVERSE LENGTH=1703	AT3G08530.1	193 kDa	12.40%	7.99%	0	1.94%	0	5.05%	2.17%	1.29%	0	0
| Symbols: | Ribosomal protein S3 family protein | chr3:19951547-19952782 FORWARD LENGTH=249	AT3G53870.1	27 kDa	16.90%	16.90%	16.90%	22.50%	16.90%	25.70%	10.40%	10.40%	10.40%	5.22%
| Symbols: | Ribosomal protein S3 family protein | chr2:13450384-13451669 FORWARD LENGTH=250	AT2G31610.1	28 kDa	20.00%	20.00%	16.80%	22.40%	16.80%	30.80%	13.60%	10.40%	5.20%	5.20%
| Symbols: | Ribosomal protein S3 family protein | chr5:13710355-13712192 REVERSE LENGTH=248	AT5G35530.1	27 kDa	20.60%	20.60%	16.90%	22.60%	16.90%	25.80%	14.10%	5.24%	5.24%	5.24%
| Symbols: EIF4A1, RH4, TIF4A1 | eukaryotic translation initiation factor 4A1 | chr3:4592635-4594128 REVERSE LENGTH=412	AT3G13920.1 (+1)	47 kDa	14.60%	12.60%	10.90%	12.10%	22.60%	16.70%	8.25%	5.83%	5.83%	5.83%
| Symbols: EIF4A-2 | eif4a-2 | chr1:20260495-20262018 FORWARD LENGTH=412	AT1G54270.1	47 kDa	10.20%	8.25%	10.90%	12.10%	22.60%	17.50%	8.25%	5.83%	5.83%	5.83%
| Symbols: EIF4A-III | eukaryotic initiation factor 4A-III | chr3:6863790-6866242 FORWARD LENGTH=408	AT3G19760.1	?	0	0	0	0	0	0	0	0	0	0
| Symbols: BIP, BIP2 | Heat shock protein 70 (Hsp 70) family protein | chr5:16807697-16810480 REVERSE LENGTH=668	AT5G42020.1	74 kDa	5.99%	10.30%	7.78%	19.50%	15.60%	23.20%	2.40%	4.64%	6.59%	4.49%
| Symbols: DAYSLEEPER | BED zinc finger ;hAT family dimerisation domain | chr3:14321838-14323928 FORWARD LENGTH=696	AT3G42170.1	79 kDa	19.00%	11.40%	0	15.90%	0	27.20%	7.18%	8.19%	4.02%	0
MLK2 | Symbols: | Protein kinase family protein | chr3:1014412-1018244 REVERSE LENGTH=701	AT3G03940.1	78 kDa	8.27%	7.99%	0	6.56%	0	1.57%	19.80%	13.00%	13.40%	9.42%
MLK1 | Symbols: | Protein kinase family protein | chr5:6010215-6013724 REVERSE LENGTH=691	AT5G18190.1	77 kDa	12.40%	10.70%	0	4.92%	0	1.59%	14.00%	14.00%	10.70%	10.70%
| Symbols: GAPB | glyceraldehyde-3-phosphate dehydrogenase B subunit | chr1:16127552-16129584 FORWARD LENGTH=447	AT1G42970.1	48 kDa	26.00%	21.70%	3.36%	34.70%	3.36%	40.50%	16.30%	12.30%	9.62%	3.36%
| Symbols: SPA1 | SPA (suppressor of phyA-105) protein family | chr2:19022572-19026821 REVERSE LENGTH=1029	AT2G46340.1	115 kDa	13.50%	8.84%	2.43%	8.07%	2.82%	8.55%	0	0	8.16%	5.83%
| Symbols: | Ribulose bisphosphate carboxylase (small chain) family protein | chr5:15377501-15378306 REVERSE LENGTH=181	AT5G38410.1	20 kDa	26.50%	22.10%	36.50%	39.20%	37.00%	39.20%	26.50%	17.70%	21.50%	17.10%
| Symbols: | Ribulose bisphosphate carboxylase (small chain) family protein | chr5:15384350-15385155 REVERSE LENGTH=181	AT5G38430.1	20 kDa	26.00%	21.50%	16.60%	18.80%	16.60%	18.80%	16.60%	7.73%	12.20%	7.73%
| Symbols: PHYA, FHY2, FRE1, HY8 | phytochrome A | chr1:3095498-3099216 REVERSE LENGTH=1122	AT1G09570.1	125 kDa	1.16%	1.34%	11.30%	5.17%	11.20%	2.41%	0.00%	2.23%	7.40%	4.37%
| Symbols: | Ribosomal protein L4/L1 family | chr3:2953813-2955444 FORWARD LENGTH=406	AT3G09630.1 (+1)	45 kDa	3.69%	3.69%	3.45%	6.65%	3.45%	21.70%	7.14%	3.69%	3.69%	0
| Symbols: | Ribosomal protein L4/L1 family | chr5:657830-659526 FORWARD LENGTH=407	AT5G02870.1 (+1)	45 kDa	6.88%	6.88%	3.44%	7.13%	3.44%	18.20%	6.63%	3.69%	0	0
| Symbols: P5CS1, ATP5CS | delta1-pyrroline-5-carboxylate synthase 1 | chr2:16598516-16602939 REVERSE LENGTH=717	AT2G39800.1 (+1)	78 kDa	12.10%	13.20%	3.35%	5.72%	3.35%	18.50%	7.81%	6.00%	3.21%	0
| Symbols: P5CS2 | delta 1-pyrroline-5-carboxylate synthase 2 | chr3:20624278-20628989 REVERSE LENGTH=726	AT3G55610.1	79 kDa	0	0	0	0	0	0	0	0	0	0
| Symbols: RBCS1A | ribulose bisphosphate carboxylase small chain 1A | chr1:25048465-25049249 REVERSE LENGTH=180	AT1G67090.1	20 kDa	26.70%	26.70%	26.70%	38.90%	27.20%	38.90%	22.20%	17.80%	7.78%	17.80%
| Symbols: ATCIMS, ATMETS, ATMS1 | Cobalamin-independent synthase family protein | chr5:5935771-5939195 FORWARD LENGTH=765	AT5G17920.1 (+1)	84 kDa	7.06%	9.93%	7.06%	8.76%	5.75%	12.70%	3.92%	1.83%	0	0
| Symbols: ATMS3, MS3 | methionine synthase 3 | chr5:7124397-7128353 REVERSE LENGTH=812	AT5G20980.1 (+1)	91 kDa	2.34%	3.20%	0	0	0	3.20%	1.97%	0	0	0
| Symbols: PGK1 | phosphoglycerate kinase 1 | chr3:4061127-4063140 REVERSE LENGTH=481	AT3G12780.1	50 kDa	16.20%	12.50%	0	17.30%	0	25.20%	10.40%	7.28%	3.53%	0
| Symbols: PGK | phosphoglycerate kinase | chr1:29924347-29926295 REVERSE LENGTH=401	AT1G79550.1 (+1)	42 kDa	8.73%	0	0	0	0	0	4.24%	4.24%	4.24%	0
| Symbols: | Phosphoglycerate kinase family protein | chr1:21028622-21030454 FORWARD LENGTH=405	AT1G56190.2	43 kDa	11.10%	0	0	11.40%	0	13.60%	0	0	0	0
| Symbols: LOS1 | Ribosomal protein S5/Elongation factor G/III/V family protein | chr1:20968245-20971077 REVERSE LENGTH=843	AT1G56070.1	94 kDa	5.93%	4.98%	2.73%	6.52%	4.15%	18.70%	2.14%	2.14%	0	0
| Symbols: | structural constituent of ribosome | chrC:82826-83482 REVERSE LENGTH=218	ATCG00800.1	25 kDa	30.70%	22.50%	9.17%	22.50%	9.17%	31.20%	4.13%	0	0	0
| Symbols: CAT2 | catalase 2 | chr4:16700937-16703215 REVERSE LENGTH=492	AT4G35090.1	57 kDa	27.40%	17.90%	6.91%	24.60%	2.85%	19.50%	12.40%	12.20%	2.24%	2.24%
| Symbols: | Ribosomal protein S27a / Ubiquitin family protein | chr1:8314940-8315410 FORWARD LENGTH=156	AT1G23410.1 (+3)	18 kDa	21.20%	21.20%	10.30%	15.40%	10.30%	15.40%	21.20%	16.00%	5.77%	16.00%
| Symbols: UBQ9 | ubiquitin 9 | chr5:14952782-14953750 REVERSE LENGTH=322	AT5G37640.1	36 kDa	0	7.76%	0	0	0	0	0	0	0	0
| Symbols: UBQ12 | ubiquitin 12 | chr1:20549533-20550225 FORWARD LENGTH=230	AT1G55060.1	26 kDa	0	0	0	0	0	0	0	0	0	0
| Symbols: UBQ8 | ubiquitin 8 | chr3:3004111-3006006 REVERSE LENGTH=631	AT3G09790.1	72 kDa	0	0	0	0	0	0	0	0	0	0
| Symbols: | GTP binding Elongation factor Tu family protein | chr1:2455559-2457001 FORWARD LENGTH=449	AT1G07920.1 (+5)	50 kDa	8.91%	6.90%	2.45%	8.91%	2.45%	14.30%	7.13%	2.45%	4.23%	2.45%
| Symbols: | Ribosomal protein L13 family protein | chr3:9064613-9065871 FORWARD LENGTH=206	AT3G24830.1	23 kDa	5.83%	5.83%	6.80%	6.80%	12.60%	6.80%	5.83%	11.70%	0.00%	0
| Symbols: | Ribosomal protein L13 family protein | chr3:2252092-2253332 FORWARD LENGTH=206	AT3G07110.1 (+1)	23 kDa	11.70%	12.10%	6.80%	6.80%	0	6.80%	11.70%	17.50%	0	0
| Symbols: | Ribosomal protein L13 family protein | chr5:19771315-19772686 REVERSE LENGTH=206	AT5G48760.1 (+1)	24 kDa	0	0	0	6.80%	0	6.80%	5.83%	5.83%	0	0
| Symbols: | Ribosomal protein L13 family protein | chr4:7655133-7656542 REVERSE LENGTH=206	AT4G13170.1	24 kDa	0	6.31%	0	6.80%	0	0	0	11.70%	0	0
| Symbols: HOG1, EMB1395, SAHH1, MEE58, ATSAHH1 | S-adenosyl-L-homocysteine hydrolase | chr4:8054931-8056676 FORWARD LENGTH=485	AT4G13940.1	53 kDa	6.39%	10.50%	5.98%	8.66%	5.98%	17.10%	2.06%	4.54%	0	0
| Symbols: SAHH2, ATSAHH2 | S-adenosyl-l-homocysteine (SAH) hydrolase 2 | chr3:8588013-8589671 REVERSE LENGTH=485	AT3G23810.1	53 kDa	4.33%	4.33%	0	1.86%	0	10.30%	0	2.47%	0	0
| Symbols: HOG1, SAHH1 | S-adenosyl-L-homocysteine hydrolase | chr4:8054931-8056763 FORWARD LENGTH=325	AT4G13940.4	36 kDa	0	0	0	0	0	0	0	0	0	0
| Symbols: CRB, CSP41B, HIP1.3 | chloroplast RNA binding | chr1:3015473-3018035 FORWARD LENGTH=378	AT1G09340.1	43 kDa	16.10%	11.60%	3.70%	4.23%	3.70%	18.00%	3.70%	3.70%	0	0
| Symbols: ACC1, AT-ACC1, EMB22, GK, PAS3 | acetyl-CoA carboxylase 1 | chr1:13534196-13543773 FORWARD LENGTH=2254	AT1G36160.1 (+1)	251 kDa	8.07%	0.80%	0	1.06%	0	2.09%	0	0	0	0
| Symbols: | Ribosomal protein S5 domain 2-like superfamily protein | chr5:6090253-6090693 REVERSE LENGTH=146	AT5G18380.1 (+1)	17 kDa	22.60%	28.10%	6.85%	22.60%	6.85%	13.00%	22.60%	22.60%	0.00%	0
| Symbols: | Ribosomal protein S5 domain 2-like superfamily protein | chr2:3781442-3781882 FORWARD LENGTH=146	AT2G09990.1	17 kDa	15.80%	21.20%	0	15.80%	0	6.16%	15.80%	15.80%	0.00%	0
| Symbols: CA1 | carbonic anhydrase 1 | chr3:194853-197873 REVERSE LENGTH=347	AT3G01500.2 (+1)	37 kDa	8.36%	8.36%	4.90%	13.80%	4.90%	18.40%	11.00%	4.61%	0.00%	0
| Symbols: CA2, CA18, BETA CA2 | carbonic anhydrase 2 | chr5:4758257-4762382 FORWARD LENGTH=331	AT5G14740.1 (+1)	37 kDa	3.93%	3.93%	5.14%	14.50%	5.14%	23.60%	3.93%	0.00%	0	0
| Symbols: ATAN11, LWD1 | Transducin/WD40 repeat-like superfamily protein | chr1:4395114-4396154 REVERSE LENGTH=346	AT1G12910.1	39 kDa	21.40%	29.50%	21.10%	30.90%	16.80%	21.10%	21.70%	19.40%	11.00%	11.00%
| Symbols: MLK4 | Protein kinase family protein | chr3:4469434-4473234 FORWARD LENGTH=703	AT3G13670.1	79 kDa	10.20%	12.70%	0	0	0	1.71%	21.20%	9.82%	10.80%	13.40%
| Symbols: RPS6, RPS6A | ribosomal protein S6 | chr4:15346306-15347714 REVERSE LENGTH=250	AT4G31700.1	28 kDa	10.80%	24.00%	0.00%	3.60%	0	20.80%	24.00%	20.80%	10.80%	4.80%
| Symbols: | Ribosomal protein L30/L7 family protein | chr2:132943-134264 REVERSE LENGTH=242	AT2G01250.1	28 kDa	19.00%	15.30%	4.96%	0	4.96%	10.30%	6.20%	9.92%	6.20%	0
| Symbols: | Ribosomal protein L30/L7 family protein | chr2:18249227-18250417 REVERSE LENGTH=247	AT2G44120.2	29 kDa	9.72%	15.00%	0	6.07%	0	10.10%	0.00%	3.64%	0	0
| Symbols: | Ribosomal protein L30/L7 family protein | chr3:4433809-4435109 FORWARD LENGTH=244	AT3G13580.1 (+2)	28 kDa	6.15%	11.50%	0	0	0	4.10%	6.15%	0.00%	0	0
| Symbols: | Coatomer, alpha subunit | chr2:9152428-9156577 FORWARD LENGTH=1218	AT2G21390.1	136 kDa	2.13%	0.00%	0	2.46%	0	10.00%	0	0	0	0
| Symbols: | Coatomer, alpha subunit | chr1:22919814-22923728 FORWARD LENGTH=1216	AT1G62020.1	137 kDa	2.55%	1.23%	0	0.00%	0	5.26%	0	0	0	0
| Symbols: CAB3, AB180, LHCB1.2 | chlorophyll A/B binding protein 3 | chr1:10472443-10473246 REVERSE LENGTH=267	AT1G29910.1 (+1)	28 kDa	7.49%	7.49%	13.90%	7.49%	13.90%	7.49%	4.49%	4.49%	4.49%	0
| Symbols: LHCB2.2, LHCB2 | photosystem II light harvesting complex gene 2.2 | chr2:1799436-1800329 REVERSE LENGTH=265	AT2G05070.1 (+2)	29 kDa	3.02%	3.02%	16.20%	3.02%	3.02%	3.02%	0	0	0.00%	0
| Symbols: CAB1, AB140, CAB140, LHCB1.3 | chlorophyll A/B binding protein 1 | chr1:10478071-10478874 FORWARD LENGTH=267	AT1G29930.1	28 kDa	7.49%	7.49%	13.90%	7.49%	13.90%	7.49%	4.49%	4.49%	4.49%	0
| Symbols: HSP60-2 | heat shock protein 60-2 | chr2:14075093-14078568 REVERSE LENGTH=585	AT2G33210.1 (+1)	62 kDa	2.22%	6.50%	2.22%	7.69%	2.22%	4.44%	2.22%	0	0	0
| Symbols: HSP60, HSP60-3B | heat shock protein 60 | chr3:8669013-8672278 FORWARD LENGTH=577	AT3G23990.1	61 kDa	2.08%	7.80%	0	7.80%	0	6.59%	5.55%	2.08%	0	0
| Symbols: | Ribosomal protein S3Ae | chr3:1329751-1331418 FORWARD LENGTH=262	AT3G04840.1	30 kDa	12.20%	12.20%	5.34%	5.34%	5.34%	16.40%	6.87%	6.87%	6.87%	0
| Symbols: | Ribosomal protein S3Ae | chr4:16548724-16550222 FORWARD LENGTH=262	AT4G34670.1	30 kDa	12.20%	12.20%	5.34%	12.60%	5.34%	16.40%	6.87%	0.00%	0	0
| Symbols: UBP12 | ubiquitin-specific protease 12 | chr5:2019545-2027834 REVERSE LENGTH=1116	AT5G06600.1 (+1)	131 kDa	0	0	0	4.48%	0	4.30%	1.08%	2.33%	0	0
| Symbols: UBP13 | ubiquitin-specific protease 13 | chr3:3761758-3770290 REVERSE LENGTH=1115	AT3G11910.1 (+1)	131 kDa	0	0	0	4.39%	1.26%	1.97%	1.08%	0.00%	0	0
| Symbols: NIT1, ATNIT1, NITI | nitrilase 1 | chr3:15986901-15988841 FORWARD LENGTH=346	AT3G44310.1 (+1)	38 kDa	11.30%	5.49%	0.00%	17.10%	5.49%	16.20%	5.49%	5.49%	5.49%	5.49%
| Symbols: ARP1, emb2207, RPL3A, RP1 | ribosomal protein 1 | chr1:16266992-16268631 FORWARD LENGTH=389	AT1G43170.1 (+7)	45 kDa	6.43%	2.57%	0.00%	4.88%	0	2.57%	2.57%	2.57%	0.00%	0
| Symbols: RD21, RD21A | Granulin repeat cysteine protease family protein | chr1:17283139-17285609 REVERSE LENGTH=462	AT1G47128.1	51 kDa	10.60%	7.79%	0	6.28%	0	7.58%	0.00%	7.58%	0.00%	0
| Symbols: ATPB, PB | ATP synthase subunit beta | chrC:52660-54156 REVERSE LENGTH=498	ATCG00480.1	54 kDa	10.40%	12.20%	0	8.43%	0	8.43%	4.82%	4.82%	2.21%	0
| Symbols: PYK10, PSR3.1, BGLU23, LEB | Glycosyl hydrolase superfamily protein | chr3:2840657-2843730 REVERSE LENGTH=524	AT3G09260.1	?	0.00%	0.00%	0.00%	0.00%	0	0.00%	0.00%	0.00%	0	0
| Symbols: KASI, KAS1 | 3-ketoacyl-acyl carrier protein synthase I | chr5:18774439-18776629 REVERSE LENGTH=473	AT5G46290.1	50 kDa	18.20%	11.40%	0	6.13%	10.80%	11.00%	8.67%	8.67%	0	0
| Symbols: RPL2.1 | ribosomal protein L2 | chrC:84337-85843 REVERSE LENGTH=274	ATCG00830.1 (+1)	30 kDa	16.10%	9.85%	0	9.85%	0	16.10%	12.80%	19.00%	0	0
| Symbols: ASN2 | asparagine synthetase 2 | chr5:25969224-25972278 FORWARD LENGTH=578	AT5G65010.1 (+1)	65 kDa	7.79%	5.88%	0	3.11%	0	10.00%	5.88%	5.88%	0	0
| Symbols: ASN1, DIN6, AT-ASN1 | glutamine-dependent asparagine synthase 1 | chr3:17438445-17441043 REVERSE LENGTH=512	AT3G47340.2	58 kDa	0	0	0	0	0	0	0	0	0	0
| Symbols: ASN3 | asparagine synthetase 3 | chr5:3212934-3216418 REVERSE LENGTH=578	AT5G10240.1 (+1)	65 kDa	0	0	0	0	0	0	0	0	0	0
| Symbols: PIP2A, PIP2, PIP2;1 | plasma membrane intrinsic protein 2A | chr3:19803906-19805454 REVERSE LENGTH=287	AT3G53420.1 (+1)	30 kDa	17.10%	17.10%	0.00%	4.53%	0	4.53%	10.50%	5.92%	0.00%	0
| Symbols: | Ribosomal protein S10p/S20e family protein | chr3:16471606-16472312 REVERSE LENGTH=124	AT3G45030.1 (+2)	14 kDa	19.40%	9.68%	9.68%	9.68%	9.68%	19.40%	9.68%	9.68%	9.68%	9.68%
| Symbols: | Ribosomal protein S10p/S20e family protein | chr3:17453671-17454437 REVERSE LENGTH=122	AT3G47370.1 (+2)	14 kDa	0	0	0	0	0	0	0	0	0	0

| Symbols: | Coatomer, beta subunit | chr4:15269460-15272693 FORWARD LENGTH=948	AT4G31490.1	106 kDa	10.10%	5.91%	0	3.90%	0	7.81%	0.00%	0	0	0
| Symbols: | Coatomer, beta subunit | chr4:15264145-15267384 FORWARD LENGTH=948	AT4G31480.1 (+1)	106 kDa	10.10%	5.91%	0	3.90%	0	6.22%	0	0	0	0
| Symbols: MLK3 | Protein kinase family protein | chr2:10985118-10988652 REVERSE LENGTH=673	AT2G25760.1	76 kDa	4.90%	6.39%	0	3.71%	0	3.71%	12.50%	8.77%	9.06%	9.06%
| Symbols: RPT3 | regulatory particle triple-A ATPase 3 | chr5:23569155-23571116 FORWARD LENGTH=408	AT5G58290.1	46 kDa	9.80%	10.00%	4.66%	4.66%	4.66%	8.09%	2.94%	2.94%	0	0.00%
| Symbols: CPN60B, LEN1 | chaperonin 60 beta | chr1:20715717-20718673 REVERSE LENGTH=600	AT1G55490.1 (+1)	64 kDa	4.17%	10.70%	0	6.33%	0	4.00%	4.33%	0	2.17%	0
| Symbols: | TCP-1/cpn60 chaperonin family protein | chr5:22874058-22876966 FORWARD LENGTH=597	AT5G56500.1 (+1)	63 kDa	0	4.52%	0	0	0	0	0	0	0	0
| Symbols: ATARCA, RACK1A_AT, RACK1A | Transducin/WD40 repeat-like superfamily protein | chr1:6222325-6223901 FORWARD LENGTH=327	AT1G18080.1	36 kDa	2.75%	8.26%	0	8.56%	0	11.30%	3.98%	0	0	0
| Symbols: RACK1B_AT | receptor for activated C kinase 1B | chr1:17981977-17983268 REVERSE LENGTH=326	AT1G48630.1	36 kDa	15.00%	0.00%	0	0	0	6.13%	0	8.28%	0	0
| Symbols: RACK1C_AT | receptor for activated C kinase 1C | chr3:6211109-6212371 REVERSE LENGTH=326	AT3G18130.1	36 kDa	0	0	0	0	0	0	0	0	0	0
| Symbols: AHA2, PMA2, HA2 | H(+)-ATPase 2 | chr4:14770820-14775920 REVERSE LENGTH=948	AT4G30190.1 (+1)	104 kDa	4.75%	3.90%	0	1.58%	0	3.27%	0	0	0	0
| Symbols: AHA1, PMA, OST2, HA1 | H(+)-ATPase 1 | chr2:8221858-8227268 FORWARD LENGTH=949	AT2G18960.1	104 kDa	4.74%	2.74%	0	1.58%	0	3.27%	0	0	0	0
| Symbols: AHA7, HA7 | H(+)-ATPase 7 | chr3:22298763-22303509 FORWARD LENGTH=961	AT3G60330.1 (+1)	106 kDa	1.04%	0	0	0	0	0	0.00%	0	0	0
| Symbols: AHA4, HA4 | H(+)-ATPase 4 | chr3:17693015-17697801 FORWARD LENGTH=960	AT3G47950.1	106 kDa	0	0	0	0	0	0	0	0	0	0
| Symbols: AHA3, ATAHA3, HA3 | H(+)-ATPase 3 | chr5:23231208-23236381 REVERSE LENGTH=949	AT5G57350.1 (+1)	104 kDa	0	0	0	0	0	0	0	0	0	0
| Symbols: AHA10 | autoinhibited H(+)-ATPase isoform 10 | chr1:5904058-5908898 FORWARD LENGTH=947	AT1G17260.1	105 kDa	0	0	0	0	0	0	0	0	0	0
| Symbols: | ATPase, F1 complex, alpha subunit protein | chr2:3361474-3364028 FORWARD LENGTH=777	AT2G07698.1	86 kDa	2.70%	2.70%	2.83%	5.66%	2.83%	7.08%	1.42%	0.00%	0	0
| Symbols: LWD2 | Transducin/WD40 repeat-like superfamily protein | chr3:9793276-9794316 FORWARD LENGTH=346	AT3G26640.1	39 kDa	21.40%	21.10%	11.00%	15.90%	11.00%	21.70%	27.20%	15.90%	11.00%	11.00%
| Symbols: | ATPase, V1 complex, subunit B protein | chr1:7016971-7020290 FORWARD LENGTH=487	AT1G20260.1 (+2)	54 kDa	10.10%	10.10%	0	6.16%	0	7.19%	3.08%	7.19%	0	0
| Symbols: ATGSTF8, ATGSTF5, GST6, GSTF8 | glutathione S-transferase phi 8 | chr2:19558213-19559266 FORWARD LENGTH=263	AT2G47730.1	29 kDa	15.60%	10.60%	0	0.00%	0	27.00%	0	0	0	0
| Symbols: ATPC1 | ATPase, F1 complex, gamma subunit protein | chr4:2350761-2351882 REVERSE LENGTH=373	AT4G04640.1	41 kDa	18.00%	9.12%	0	6.17%	7.24%	6.17%	3.49%	6.17%	2.68%	0
| Symbols: FBA2 | fructose-bisphosphate aldolase 2 | chr4:18163714-18165659 REVERSE LENGTH=398	AT4G38970.1	43 kDa	10.10%	10.80%	0	13.80%	7.04%	14.60%	10.80%	7.04%	0	0
| Symbols: RPL16A | ribosomal protein large subunit 16A | chr2:17791794-17792946 FORWARD LENGTH=182	AT2G42740.1 (+4)	21 kDa	12.10%	12.10%	7.69%	7.69%	7.69%	7.69%	7.69%	12.10%	12.10%	7.69%
| Symbols: | Transketolase | chr3:22454004-22456824 FORWARD LENGTH=741	AT3G60750.1 (+1)	80 kDa	6.21%	4.18%	0	0.00%	0	18.60%	2.43%	0	0	0
| Symbols: | binding | chr5:19317899-19327014 FORWARD LENGTH=1606	AT5G47690.2 (+1)	181 kDa	5.35%	0	0	2.37%	0	5.98%	0	0	0	0
| Symbols: SAC52, RPL10, RPL10A | Ribosomal protein L16p/L10e family protein | chr1:4888270-4889408 FORWARD LENGTH=220	AT1G14320.1	25 kDa	5.45%	10.50%	5.45%	5.00%	0	5.00%	10.50%	10.50%	5.45%	5.45%
| Symbols: CAC3 | acetyl Co-enzyme a carboxylase carboxyltransferase alpha subunit | chr2:15917612-15920749 FORWARD LENGTH=769	AT2G38040.1 (+1)	85 kDa	6.63%	2.47%	0	0	0	7.41%	0	0	0	0
| Symbols: RAN-1, RAN1, ATRAN1 | RAS-related nuclear protein-1 | chr5:6760364-6761747 FORWARD LENGTH=221	AT5G20010.1 (+2)	25 kDa	10.40%	15.40%	0	11.30%	0	31.20%	5.88%	0	0	0
| Symbols: | Ribosomal protein S4 | chr5:4935124-4936334 REVERSE LENGTH=198	AT5G15200.1	23 kDa	9.60%	9.60%	7.58%	13.10%	7.58%	13.10%	4.55%	0	0	0
| Symbols: | Ribosomal protein S26e family protein | chr2:16918506-16919623 FORWARD LENGTH=133	AT2G40510.1 (+2)	15 kDa	18.00%	18.00%	0	18.00%	0	18.00%	18.00%	18.00%	6.77%	0
| Symbols: EMB2296 | Ribosomal protein L2 family | chr2:7837151-7838160 FORWARD LENGTH=258	AT2G18020.1	28 kDa	12.00%	7.36%	7.36%	4.65%	7.36%	7.36%	7.36%	7.36%	7.36%	0.00%
| Symbols: | Ribosomal protein L2 family | chr4:17097613-17098656 FORWARD LENGTH=258	AT4G36130.1	28 kDa	12.00%	7.36%	0	4.65%	0	0	0	7.36%	0	0
| Symbols: | Histone superfamily protein | chr1:2369212-2369523 FORWARD LENGTH=103	AT1G07660.1 (+8)	11 kDa	9.71%	9.71%	7.77%	29.10%	7.77%	29.10%	9.71%	9.71%	9.71%	9.71%
| Symbols: | oxidoreductases, acting on the aldehyde or oxo group of donors, NAD or NADP as acceptor;copper ion binding | chr2:8613168-8615649 FORWARD LENGTH=401	AT2G19940.1 (+1)	44 kDa	19.70%	3.99%	0	3.99%	0	8.98%	3.99%	0	0	0
| Symbols: ATRPS5B, RPS5B | ribosomal protein 5B | chr2:15647883-15649042 REVERSE LENGTH=207	AT2G37270.1 (+3)	23 kDa	17.90%	6.28%	0	0	0	0	13.50%	13.50%	13.50%	7.25%
| Symbols: | Thioredoxin superfamily protein | chr3:3672189-3673937 FORWARD LENGTH=266	AT3G11630.1	29 kDa	0	6.39%	0	0.00%	0	12.40%	6.39%	10.90%	0	0
| Symbols: PIP1B, TMP-A, ATHH2, PIP1;2 | plasma membrane intrinsic protein 1B | chr2:18910450-18911703 FORWARD LENGTH=286	AT2G45960.1 (+2)	31 kDa	3.50%	3.50%	0.00%	9.79%	6.29%	9.79%	3.50%	3.50%	0	0
| Symbols: RPL27A, RPL27AB | Ribosomal protein L18e/L15 superfamily protein | chr1:8263007-8263447 FORWARD LENGTH=146	AT1G23290.1 (+1)	16 kDa	10.30%	10.30%	0.00%	6.85%	0	8.22%	17.10%	10.30%	10.30%	0
| Symbols: | Ribosomal protein L18ae/LX family protein | chr2:14532916-14534161 REVERSE LENGTH=178	AT2G34480.1	21 kDa	8.99%	4.49%	0	9.55%	0	4.49%	4.49%	0.00%	4.49%	0
| Symbols: | Ribosomal protein L18ae/LX family protein | chr3:4910773-4911933 FORWARD LENGTH=178	AT3G14600.1	21 kDa	0	0	0	5.06%	0	0	0	0	0	0
| Symbols: EMB2719, HAP15 | PAM domain (PCI/PINT associated module) protein | chr1:7001409-7004154 REVERSE LENGTH=488	AT1G20200.1	56 kDa	8.20%	8.81%	0	2.66%	0	9.02%	0	0	0	0
| Symbols: | PAM domain (PCI/PINT associated module) protein | chr1:28524623-28526718 REVERSE LENGTH=487	AT1G75990.1	56 kDa	8.21%	0	0	2.67%	0	9.03%	0	0	0	0
| Symbols: APS1 | ATP sulfurylase 1 | chr3:8112837-8114734 FORWARD LENGTH=463	AT3G22890.1	51 kDa	9.07%	2.59%	0	6.70%	0	9.29%	0	0	0	0
| Symbols: APS2, ASA1 | Pseudouridine synthase/archaeosine transglycosylase-like family protein | chr1:6914835-6916657 REVERSE LENGTH=476	AT1G19920.1	54 kDa	0	0	0	0	0	0	0	0	0	0
| Symbols: APS3 | Pseudouridine synthase/archaeosine transglycosylase-like family protein | chr4:8413443-8415311 REVERSE LENGTH=465	AT4G14680.1	52 kDa	0	0	0	0	0	0	0	0	0	0
| Symbols: | Tetratricopeptide repeat (TPR)-like superfamily protein | chr3:22565440-22566678 REVERSE LENGTH=412	AT3G60980.1	47 kDa	0	0	0	0	0	0	13.80%	14.10%	0	0
| Symbols: SPA2 | SPA1-related 2 | chr4:6772163-6776675 FORWARD LENGTH=1036	AT4G11110.1	115 kDa	10.60%	11.60%	0	0	0	0	0	0	0	0.00%
| Symbols: PORB | protochlorophyllide oxidoreductase B | chr4:13725648-13727107 FORWARD LENGTH=401	AT4G27440.1 (+1)	43 kDa	12.50%	7.48%	0	2.99%	0	2.99%	2.99%	0	0	0
| Symbols: ATPE | ATP synthase epsilon chain | chrC:52265-52663 REVERSE LENGTH=132	ATCG00470.1	14 kDa	27.30%	27.30%	0	11.40%	0	11.40%	15.90%	27.30%	0	0
| Symbols: RPL14 | ribosomal protein L14 | chrC:80696-81064 REVERSE LENGTH=122	ATCG00780.1	14 kDa	21.30%	21.30%	0	13.10%	0	23.80%	13.10%	21.30%	0.00%	0.00%
| Symbols: | C2 calcium/lipid-binding plant phosphoribosyltransferase family protein | chr5:2127200-2129584 REVERSE LENGTH=794	AT5G06850.1	91 kDa	0	0	0	4.66%	0	8.44%	0	0	0	0
| Symbols: FDH | formate dehydrogenase | chr5:4777043-4779190 FORWARD LENGTH=384	AT5G14780.1	42 kDa	9.38%	3.12%	0	7.03%	0	16.40%	0	7.03%	0	0
| Symbols: TOC1, APRR1, PRR1, AtTOC1 | CCT motif -containing response regulator protein | chr5:24675540-24678176 FORWARD LENGTH=618	AT5G61380.1	69 kDa	4.05%	4.05%	5.34%	0	7.28%	0	1.94%	0	2.10%	2.10%
| Symbols: ELF4-L2 | ELF4-like 2 | chr1:27344988-27345347 FORWARD LENGTH=119	AT1G72630.1	13 kDa	0	10.90%	0	12.60%	0	10.90%	21.80%	21.80%	21.80%	10.90%
| Symbols: STV1, RPL24B, RPL24 | Ribosomal protein L24e family protein | chr3:19660749-19661912 REVERSE LENGTH=163	AT3G53020.1	19 kDa	14.70%	14.70%	0	7.36%	0	7.36%	14.70%	14.70%	0	0
| Symbols: | glycine-rich protein | chr4:11944050-11945565 REVERSE LENGTH=356	AT4G22740.1 (+1)	39 kDa	12.10%	12.10%	0	0	0	12.10%	7.58%	0.00%	0	0
| Symbols: | Ribosomal L28e protein family | chr2:8511752-8512995 FORWARD LENGTH=143	AT2G19730.1 (+2)	16 kDa	13.30%	13.30%	0	0	0	24.50%	0.00%	0.00%	24.50%	0
| Symbols: | Ribosomal protein S11 family protein | chr2:15169925-15171159 FORWARD LENGTH=150	AT2G36160.1	16 kDa	16.00%	16.00%	8.67%	23.30%	8.67%	23.30%	0	0	0	0
| Symbols: | Ribosomal protein S11 family protein | chr3:3623757-3624866 REVERSE LENGTH=150	AT3G11510.1	16 kDa	8.67%	16.00%	8.67%	16.00%	8.67%	16.00%	0	0	0	0
| Symbols: | Coatomer, beta' subunit | chr3:5411699-5418313 REVERSE LENGTH=909	AT3G15980.1 (+3)	102 kDa	3.74%	1.10%	0	0.00%	0	5.28%	0	0	0	0
| Symbols: | structural molecules | chr1:30084522-30091949 FORWARD LENGTH=1135	AT1G79990.1 (+2)	129 kDa	2.11%	0	0	0	0	1.76%	0	0	0	0
| Symbols: XIK, ATXIK, XI-17 | Myosin family protein with Dil domain | chr5:6927064-6936825 REVERSE LENGTH=1545	AT5G20490.1 (+1)	175 kDa	0.91%	2.20%	0	0	0	0	0	0	0	0
| Symbols: MYA1, ATMYA1, XI-1 | myosin 1 | chr1:6039453-6049309 FORWARD LENGTH=1520	AT1G17580.1	173 kDa	1.38%	1.32%	0	0	0	0	0	0	0	0
| Symbols: XIC, ATXIC | Myosin family protein with Dil domain | chr1:2779963-2788325 FORWARD LENGTH=1538	AT1G08730.1	175 kDa	0	1.30%	0	0.00%	0	0	0	0	0	0
| Symbols: HSP81-2, ERD8, HSP90.2, AtHsp90.2 | heat shock protein 81-2 | chr5:22686923-22689433 FORWARD LENGTH=699	AT5G56030.1 (+1)	80 kDa	5.72%	4.01%	0	2.00%	0	2.00%	2.00%	0	0	0
| Symbols: PIP3, PIP3A, PIP2;7, SIMIP | plasma membrane intrinsic protein 3 | chr4:16708672-16709958 FORWARD LENGTH=280	AT4G35100.1 (+1)	30 kDa	17.10%	17.10%	6.79%	11.10%	6.79%	0	11.10%	0	0	0
| Symbols: RPS4 | chloroplast ribosomal protein S4 | chrC:45223-45828 REVERSE LENGTH=201	ATCG00380.1	23 kDa	3.98%	3.98%	0	0	0	0.00%	8.96%	8.96%	0	0
| Symbols: LOS2, ENO2 | Enolase | chr2:15321081-15323786 REVERSE LENGTH=444	AT2G36530.1	48 kDa	3.38%	3.38%	0	3.38%	0	7.88%	3.38%	0	3.60%	0
| Symbols: | ATPase, V0/A0 complex, subunit C/D | chr3:10778025-10780350 FORWARD LENGTH=351	AT3G28715.1	41 kDa	9.12%	5.70%	0	4.27%	0	4.27%	5.70%	0	0	0
| Symbols: | ATPase, V0/A0 complex, subunit C/D | chr3:10773144-10775594 REVERSE LENGTH=351	AT3G28710.1	41 kDa	5.70%	5.70%	0	9.97%	0	9.97%	0	0	0	0
| Symbols: cpHsc70-1 | chloroplast heat shock protein 70-1 | chr4:12590094-12593437 FORWARD LENGTH=718	AT4G24280.1	77 kDa	0	0	0	2.92%	0	8.77%	1.95%	0	0	0
| Symbols: CPHSC70-2EAT SHOCK PROTEIN 70-2, HSC70-7, cpHsc70-2 | chloroplast heat shock protein 70-2 | chr5:20303470-20306295 FORWARD LENGTH=718	AT5G49910.1	77 kDa	0	0	0	5.15%	0	6.55%	1.95%	0	0	0
| Symbols: LUH | LEUNIG_homolog | chr2:13867235-13871844 FORWARD LENGTH=787	AT2G32700.1 (+6)	86 kDa	0	0	0	1.52%	0	6.48%	0	0	0	0
| Symbols: emb1138 | DEAD box RNA helicase (RH3) | chr5:9285540-9288871 REVERSE LENGTH=747	AT5G26742.1 (+1)	81 kDa	5.62%	6.02%	0	1.87%	0	0.00%	0	0	0	0
| Symbols: RPS11 | ribosomal protein S11 | chrC:78960-79376 REVERSE LENGTH=138	ATCG00750.1	15 kDa	15.90%	15.90%	0	6.52%	0	6.52%	15.90%	15.90%	0.00%	0
| Symbols: | Ribosomal protein S4 (RPS4A) family protein | chr2:7546598-7548138 FORWARD LENGTH=261	AT2G17360.1 (+3)	30 kDa	10.30%	0.00%	7.28%	7.28%	0	7.28%	3.45%	0	0	0
| Symbols: PFL, RPS18A, PFL1 | Ribosomal protein S13/S18 family | chr1:8067990-8069163 FORWARD LENGTH=152	AT1G22780.1 (+2)	18 kDa	0	5.26%	5.26%	11.20%	0.00%	5.92%	0.00%	5.26%	5.26%	0
| Symbols: P40, AP40, RP40, RPSAA | 40s ribosomal protein SA | chr1:27243148-27244842 REVERSE LENGTH=298	AT1G72370.1 (+1)	32 kDa	3.69%	10.70%	0	7.05%	0	4.36%	0	0	0	0
| Symbols: RPSAb | 40s ribosomal protein SA B | chr3:1309544-1310846 REVERSE LENGTH=280	AT3G04770.2	31 kDa	0.00%	0	0	2.86%	0	0	0	0	0	0
| Symbols: CLPC, ATHSP93-V, HSP93-V, DCA1, CLPC1 | CLPC homologue 1 | chr5:20715710-20719800 REVERSE LENGTH=929	AT5G50920.1	?	0.00%	0.00%	0	0	0	0.00%	0	0	0	0
| Symbols: ATCLPC, ATHSP93-III, HSP93-III | Clp ATPase | chr3:18122363-18126008 REVERSE LENGTH=952	AT3G48870.1 (+1)	106 kDa	5.15%	3.47%	0	0	0	2.31%	0	0	0	0
| Symbols: SPA4 | SPA1-related 4 | chr1:19783748-19786690 FORWARD LENGTH=794	AT1G53090.1 (+1)	89 kDa	6.30%	13.60%	0	0	0	0	0	0	0	0
| Symbols: | AAA-type ATPase family protein | chr2:8692736-8694837 FORWARD LENGTH=443	AT2G20140.1 (+1)	49 kDa	14.90%	12.00%	0	0	0	0	2.71%	0	0	0
| Symbols: RPT6A, ATSUG1 | regulatory particle triple-A ATPase 6A | chr5:6752144-6754918 FORWARD LENGTH=419	AT5G19990.1 (+1)	47 kDa	2.86%	2.86%	0	5.25%	0	9.55%	2.86%	2.86%	0	0.00%
| Symbols: | Lactate/malate dehydrogenase family protein | chr1:1189418-1191267 REVERSE LENGTH=332	AT1G04410.1	36 kDa	0.00%	9.94%	0	6.02%	0	6.02%	0.00%	0	0	0
| Symbols: | Aldolase superfamily protein | chr3:19627383-19628874 REVERSE LENGTH=358	AT3G52930.1	39 kDa	0	3.63%	0	3.91%	0	15.90%	7.82%	0	6.42%	0

| Symbols: | Aldolase superfamily protein | chr5:963389-964982 REVERSE LENGTH=359	AT5G03690.2	39 kDa	0	0	0	3.90%	0	0	0	0	0	0
| Symbols: | Aldolase superfamily protein | chr4:13391566-13392937 FORWARD LENGTH=358	AT4G26530.1 (+1)	38 kDa	0	0	0	0	0	3.91%	0	0	0	0
| Symbols: CHLI1, CH42, CH-42, CHL11, CHLI-1 | P-loop containing nucleoside triphosphate hydrolases superfamily protein | chr4:10201897-10203361 REVERSE LENGTH=424	AT4G18480.1	46 kDa	8.49%	4.95%	0	0.00%	0	7.31%	0	0	0	0
| Symbols: CHLI2, CHL I2, CHLI-2 | magnesium chelatase i2 | chr5:18628095-18629565 FORWARD LENGTH=418	AT5G45930.1	46 kDa	0	0	0	0	0	0	0	0	0	0
| Symbols: | transducin family protein / WD-40 repeat family protein | chr3:23431009-23437241 REVERSE LENGTH=1104	AT3G63460.1 (+2)	120 kDa	1.18%	1.18%	0	1.36%	0	2.54%	0	0	0	0
| Symbols: | vacuolar ATP synthase subunit H family protein | chr3:14228846-14232228 REVERSE LENGTH=441	AT3G42050.1	50 kDa	6.35%	2.95%	0	4.08%	0	2.95%	0	0	0	0
| Symbols: RPL18 | ribosomal protein L18 | chr3:1621511-1622775 FORWARD LENGTH=187	AT3G05590.1 (+1)	21 kDa	12.80%	5.88%	6.95%	5.88%	0	13.40%	5.88%	5.88%	0	0
| Symbols: RPT5A, ATS6A.2 | regulatory particle triple-A ATPase 5A | chr3:1603540-1605993 FORWARD LENGTH=424	AT3G05530.1	47 kDa	13.70%	7.55%	0	0	0	4.72%	7.55%	7.55%	0	0.00%
| Symbols: AGT, AGT1, SGAT | alanine:glyoxylate aminotransferase | chr2:5539417-5540902 REVERSE LENGTH=401	AT2G13360.1 (+1)	44 kDa	3.99%	3.99%	4.24%	0	4.24%	6.98%	0	0	0	0
| Symbols: | Ribosomal protein L22p/L17e family protein | chr1:9515230-9516725 FORWARD LENGTH=176	AT1G27400.1 (+1)	20 kDa	0	5.11%	10.80%	0.00%	5.68%	15.90%	0	0	0	0
| Symbols: VHA-A | vacuolar ATP synthase subunit A | chr1:29660463-29664575 FORWARD LENGTH=623	AT1G78900.1 (+1)	69 kDa	1.77%	0.00%	0	0	0	4.33%	0	0	0	0
| Symbols: RPT1A | regulatory particle triple-A 1A | chr1:20065921-20068324 REVERSE LENGTH=426	AT1G53750.1	48 kDa	5.63%	2.11%	0	3.05%	0	3.05%	0.00%	0	0	0
| Symbols: ATJ2, J2 | DNAJ homologue 2 | chr5:7303798-7305668 REVERSE LENGTH=419	AT5G22060.1	46 kDa	6.21%	6.21%	0	0	0	2.86%	0	0	0	0
| Symbols: ATJ3, ATJ | DNAJ homologue 3 | chr3:15869115-15871059 REVERSE LENGTH=420	AT3G44110.1 (+1)	46 kDa	6.19%	6.19%	0	0	0	5.95%	0	0	0	0
| Symbols: RPS1, ARRPS1 | ribosomal protein S1 | chr5:11619262-11621223 REVERSE LENGTH=416	AT5G30510.1	45 kDa	3.61%	0	0	8.17%	0	10.60%	0	0	0	0
| Symbols: ACCD | acetyl-CoA carboxylase carboxyl transferase subunit beta | chrC:57075-58541 FORWARD LENGTH=488	ATCG00500.1	56 kDa	6.56%	2.87%	0	0	0	2.66%	0	0	0	0
| Symbols: RPS8 | ribosomal protein S8 | chrC:80068-80472 REVERSE LENGTH=134	ATCG00770.1	15 kDa	8.96%	17.20%	0	8.96%	0	8.96%	8.21%	8.21%	0	0
| Symbols: SSR16 | small subunit ribosomal protein 16 | chr4:16535084-16536092 REVERSE LENGTH=113	AT4G34620.1	13 kDa	13.30%	13.30%	0	13.30%	0	13.30%	13.30%	13.30%	25.70%	0
| Symbols: | Sucrose-6F-phosphate phosphohydrolase family protein | chr2:15053952-15055776 FORWARD LENGTH=422	AT2G35840.1 (+2)	48 kDa	4.50%	0	0	0	0	6.16%	0	0	0	0
| Symbols: | GTP binding Elongation factor Tu family protein | chr4:1295751-1298354 REVERSE LENGTH=454	AT4G02930.1	49 kDa	8.15%	0	0	3.52%	0	8.37%	0	0	0	0
| Symbols: ECT2 | evolutionarily conserved C-terminal region 2 | chr3:4385274-4388220 REVERSE LENGTH=667	AT3G13460.1 (+3)	72 kDa	7.50%	5.55%	0	0.00%	0	0	0	0	0	0
| Symbols: | Glycine cleavage T-protein family | chr1:4001801-4003245 FORWARD LENGTH=408	AT1G11860.1 (+2)	44 kDa	5.64%	5.64%	0	5.64%	0	5.64%	9.56%	0	0	0
| Symbols: NOP56 | homolog of nucleolar protein NOP56 | chr1:20984544-20986893 REVERSE LENGTH=522	AT1G56110.1	59 kDa	2.87%	0.00%	0	0	0	5.94%	0	0	0	0
| Symbols: RPS15A | ribosomal protein S15A | chr1:2408413-2409065 REVERSE LENGTH=130	AT1G07770.1 (+2)	15 kDa	6.92%	6.92%	10.80%	0	10.80%	23.10%	0	0	0	0
| Symbols: | Ribosomal protein S8 family protein | chr2:16517588-16518265 REVERSE LENGTH=136	AT2G39590.1	15 kDa	0	0	0	0	0	0	0	0	0	0
| Symbols: RPN1A, ATRPN1A | 26S proteasome regulatory subunit S2 1A | chr2:8859211-8864699 FORWARD LENGTH=891	AT2G20580.1	?	0.00%	0.00%	0	0	0	0.00%	0	0	0	0
| Symbols: PAC1 | 20S proteasome alpha subunit C1 | chr3:7792819-7793571 REVERSE LENGTH=250	AT3G22110.1	27 kDa	0	11.20%	0	0	0	4.80%	6.40%	6.40%	0	0
| Symbols: ATBBC1, BBC1, RSU2 | breast basic conserved 1 | chr3:18166971-18168047 REVERSE LENGTH=206	AT3G49010.1 (+3)	24 kDa	0	0.00%	0	11.70%	0	17.00%	11.70%	0.00%	0	0
| Symbols: | Tetratricopeptide repeat (TPR)-like superfamily protein | chr3:22535729-22536940 FORWARD LENGTH=403	AT3G60960.1	46 kDa	0	0	0	0	0	0	2.23%	4.71%	0	0
| Symbols: CPN60A, CH-CPN60A, SLP | chaperonin-60alpha | chr2:11926603-11929184 FORWARD LENGTH=586	AT2G28000.1	62 kDa	4.44%	2.22%	0	0.00%	0	0.00%	0	0	0	0
| Symbols: ATTIP49A, RIN1 | P-loop containing nucleoside triphosphate hydrolases superfamily protein | chr5:7391026-7394071 REVERSE LENGTH=458	AT5G22330.1	50 kDa	0.00%	5.02%	0	5.24%	0	2.40%	0	0	0	0
| Symbols: RPL16 | ribosomal protein L16 | chrC:81189-82652 REVERSE LENGTH=135	ATCG00790.1	15 kDa	10.40%	0.00%	0	10.40%	0	20.70%	0	0	0	0
| Symbols: PUB12, AtPUB12 | PLANT U-BOX 12 | chr2:12367001-12370608 REVERSE LENGTH=962	AT2G28830.1	107 kDa	1.46%	0.00%	0	1.46%	0	1.46%	0	0	0	0
| Symbols: | NAD(P)-binding Rossmann-fold superfamily protein | chr3:11193767-11194948 REVERSE LENGTH=298	AT3G29250.1	32 kDa	0	0	0	4.03%	0	18.10%	0	0	0	0
| Symbols: ILA | ILITYHIA | chr1:24065232-24081908 REVERSE LENGTH=2610	AT1G64790.1 (+1)	285 kDa	2.07%	0	0	0.00%	0	0	0	0	0	0
| Symbols: APUM5, PUM5 | pumilio 5 | chr3:7059098-7062660 REVERSE LENGTH=961	AT3G20250.1	107 kDa	3.75%	1.04%	0	1.04%	0	0	0	0	0	0
| Symbols: ATTPS7, TPS7, ATTPSA | trehalose-phosphatase/synthase 7 | chr1:1955413-1958153 FORWARD LENGTH=851	AT1G06410.1	97 kDa	1.29%	0	0	0	0	3.41%	0	0	0	0
| Symbols: | transcription regulators | chr1:373335-386682 FORWARD LENGTH=2431	AT1G02080.1 (+1)	270 kDa	1.93%	0.74%	0	0	0	0	0	0	0	0
| Symbols: ATGSTF9, GLUTTR, ATGSTF7, GSTF9 | glutathione S-transferase PHI 9 | chr2:13139132-13140057 FORWARD LENGTH=215	AT2G30860.1 (+1)	24 kDa	0	16.70%	0	3.72%	0	0	0	0	0	0
| Symbols: | Ribosomal protein L35Ae family protein | chr1:27928415-27929466 REVERSE LENGTH=112	AT1G74270.1	13 kDa	22.30%	0	0	33.00%	0	22.30%	0	0	0	0
| Symbols: ATU2AF35B, U2AF35B | Zinc finger C-x8-C-x5-C-x3-H type family protein | chr5:17170445-17171296 REVERSE LENGTH=283	AT5G42820.1 (+1)	33 kDa	8.13%	0	0	6.36%	0	6.36%	0	0	0	0
| Symbols: ATU2AF35A | U2 snRNP auxiliary factor small subunit, putative | chr1:9615302-9616042 FORWARD LENGTH=246	AT1G27650.2	29 kDa	0	0	0	7.32%	0	7.32%	0	0	0	0
| Symbols: | Zinc finger C-x8-C-x5-C-x3-H type family protein | chr3:16328221-16328448 REVERSE LENGTH=75	AT3G44785.1	9 kDa	0	0	0	0	0	0	0	0	0	0
| Symbols: PIL6, PIF5 | phytochrome interacting factor 3-like 6 | chr3:21828189-21829895 REVERSE LENGTH=442	AT3G59060.1 (+3)	49 kDa	0	0.00%	2.71%	0	0	0	9.95%	0	0	0
| Symbols: PIF4, SRL2, AtPIF4 | phytochrome interacting factor 4 | chr2:17887003-17888823 FORWARD LENGTH=430	AT2G43010.1 (+1)	48 kDa	0	0	2.79%	0	0	0.00%	2.79%	0	0	0
| Symbols: | Ribosomal protein L31e family protein | chr2:8513577-8514346 FORWARD LENGTH=119	AT2G19740.1 (+2)	14 kDa	0	0	0	31.90%	0	31.90%	0	0	0	0
| Symbols: | Ribosomal protein L31e family protein | chr5:22944003-22944767 REVERSE LENGTH=85	AT5G56710.2	10 kDa	0	0	0	9.41%	0	9.41%	0	0	0	0
| Symbols: | Coatomer epsilon subunit | chr1:10858546-10860173 REVERSE LENGTH=292	AT1G30630.1	33 kDa	0	0	0	14.70%	0	14.70%	0	0	0	0
| Symbols: SHM4 | serine hydroxymethyltransferase 4 | chr4:8048013-8050021 REVERSE LENGTH=471	AT4G13930.1	52 kDa	6.37%	3.40%	0	0	0	0	0	0	0	0
| Symbols: RPL22 | ribosomal protein L22 | chrC:83467-83949 REVERSE LENGTH=160	ATCG00810.1	19 kDa	0	0	0	8.12%	0	14.40%	0	0	0	0
| Symbols: PBE1 | 20S proteasome beta subunit E1 | chr1:4452641-4454663 FORWARD LENGTH=274	AT1G13060.1 (+2)	30 kDa	0	14.20%	0	0	0	0	0	0.00%	0	0
| Symbols: PSBB | photosystem II reaction center protein B | chrC:72371-73897 FORWARD LENGTH=508	ATCG00680.1	56 kDa	3.35%	2.17%	0	0	0	0	5.51%	0	0	0
| Symbols: NRPB1, RPB1, RNA_POL_II_LSRNA_POL_II_LS, RNA_POL_II_LS | RNA polymerase II large subunit | chr4:16961115-16967892 REVERSE LENGTH=1839	AT4G35800.1	205 kDa	1.96%	0	0	0	0	0.00%	0	0	0	0
| Symbols: EMB2761 | threonyl-tRNA synthetase, putative / threonine--tRNA ligase, putative | chr2:1698466-1701271 REVERSE LENGTH=650	AT2G04842.1	75 kDa	0	0	0	0.00%	0	4.00%	0	0	0	0
| Symbols: ELF4-L4 | ELF4-like 4 | chr1:5997932-5998276 FORWARD LENGTH=114	AT1G17455.1 (+1)	13 kDa	0	0	0	0	0	0	22.80%	22.80%	11.40%	0.00%
| Symbols: | AAA-type ATPase family protein | chr1:17009220-17011607 FORWARD LENGTH=399	AT1G45000.1 (+2)	45 kDa	6.02%	6.02%	0	0	0	0	6.02%	0	0	0
| Symbols: PHOT1, NPH1, JK224, RPT1 | phototropin 1 | chr3:16818557-16823960 FORWARD LENGTH=996	AT3G45780.1 (+1)	112 kDa	2.51%	0	0	0	0	2.11%	0	0	0	0
| Symbols: PHOT2, NPL1 | phototropin 2 | chr5:23524771-23529993 FORWARD LENGTH=915	AT5G58140.1 (+3)	102 kDa	0	0	0	0	0	0	0	0	0	0
| Symbols: ZFN1 | zinc finger protein 1 | chr3:614075-615916 FORWARD LENGTH=397	AT3G02830.1	44 kDa	8.06%	0	0	0	0	0	0	0	0	0
| Symbols: | Ribosomal protein L35Ae family protein | chr1:15651585-15652427 REVERSE LENGTH=111	AT1G41880.1	13 kDa	0	0	0	33.30%	0	22.50%	0	0	0	0
| Symbols: SPA3 | SPA1-related 3 | chr3:5169327-5172480 REVERSE LENGTH=837	AT3G15354.1	93 kDa	2.03%	3.70%	0	0	0	0	0	0	0	0
| Symbols: PRP39 | Tetratricopeptide repeat (TPR)-like superfamily protein | chr1:1051803-1056550 FORWARD LENGTH=768	AT1G04080.1 (+1)	85 kDa	4.95%	0	0	0	0	0	0	0	0	0
| Symbols: ABA1, LOS6, NPQ2, ATABA1, ZEP, IBS3, ATZEP | zeaxanthin epoxidase (ZEP) (ABA1) | chr5:26753745-26757090 REVERSE LENGTH=667	AT5G67030.1 (+1)	74 kDa	4.65%	0	0	0	0	0	0	0	0	0

Exclusive Unique Peptide Count

Identified Proteins (283)	Accession Number	Molecular Weight	AtELF3-1	AtELF3-2	SvELF3#2 rep1	SvELF3#2 rep2	SvELF3#3 rep1	SvELF3#3 rep2	BdELF3#2 rep1	BdELF3#2 rep2	BdELF3#3 rep1	BdELF3#3 rep2
| Symbols: PHYB, HY3, OOP1 | phytochrome B | chr2:8140079-8144151 FORWARD LENGTH=1172	AT2G18790.1	129 kDa	23	24	31	28	32	30	22	22	24	24
| Symbols: PHYD | phytochrome D | chr4:9195602-9199486 REVERSE LENGTH=1164	AT4G16250.1	129 kDa	1	1	2	1	3	2	2	1	2	2
| Symbols: HSC70-1, HSP70-1, AT-HSC70-1, HSC70 | heat shock cognate protein 70-1 | chr5:554055-556334 REVERSE LENGTH=651	AT5G02500.1	71 kDa	3	2	9	7	9	9	2	2	2	2
| Symbols: | Heat shock protein 70 (Hsp 70) family protein | chr3:2903434-2905632 REVERSE LENGTH=649	AT3G09440.1 (+1)	71 kDa	6	6	8	8	9	8	6	6	2	3
| Symbols: HSP70, ATHSP70 | heat shock protein 70 | chr3:3991487-3993689 REVERSE LENGTH=650	AT3G12580.1	71 kDa	2	1	1	4	2	4	2	1	0	0
| Symbols: | Heat shock protein 70 (Hsp 70) family protein | chr5:550296-552565 REVERSE LENGTH=653	AT5G02490.1	71 kDa	4	3	1	2	2	4	2	1	0	0
| Symbols: Hsp70b | heat shock protein 70B | chr1:5502386-5504326 REVERSE LENGTH=646	AT1G16030.1	71 kDa	0	0	0	0	0	0	0	0	0	0
| Symbols: BdELF3_HFC	ATMGcustom02	86 kDa	0	0	0	0	0	0	25	26	21	19
| Symbols: PHYC | phytochrome C | chr5:14008049-14011619 FORWARD LENGTH=1111	AT5G35840.1	124 kDa	22	23	27	32	27	33	20	18	19	22
| Symbols: SvELF3_HFC	ATMGcustom01	87 kDa	0	0	19	29	22	33	0	0	0	0
| Symbols: AtELF3_HFC	ATMGcustom03	84 kDa	20	19	0	0	0	0	0	0	0	0
| Symbols: TZP | zinc knuckle (CCHC-type) family protein | chr5:17527261-17530207 FORWARD LENGTH=831	AT5G43630.1	91 kDa	14	12	13	20	13	24	12	13	14	14
| Symbols: RBCL | ribulose-bisphosphate carboxylases | chrC:54958-56397 FORWARD LENGTH=479	ATCG00490.1	53 kDa	14	13	13	18	11	19	14	14	9	10
| Symbols: PHYE | phytochrome E | chr4:10042312-10045948 REVERSE LENGTH=1112	AT4G18130.1	123 kDa	11	19	12	18	11	17	6	10	14	13
| Symbols: TUB2 | tubulin beta chain 2 | chr5:25181560-25183501 FORWARD LENGTH=450	AT5G62690.1 (+1)	51 kDa	3	3	1	3	1	4	3	2	1	1
| Symbols: TUB5 | tubulin beta-5 chain | chr1:6938033-6940481 REVERSE LENGTH=449	AT1G20010.1	50 kDa	4	2	0	3	0	3	2	1	0	0
| Symbols: TUB6 | beta-6 tubulin | chr5:3961317-3962971 REVERSE LENGTH=449	AT5G12250.1	51 kDa	2	1	0	1	0	1	1	0	0	0
| Symbols: TUB9 | tubulin beta-9 chain | chr4:11182218-11183840 FORWARD LENGTH=444	AT4G20890.1	50 kDa	1	1	0	1	0	1	0	1	0	0

<table>
<tr><th></th><th></th><th></th><th></th><th></th><th></th><th></th><th></th><th></th><th></th><th></th><th></th><th></th></tr>
<tr><td>| Symbols: TUB7 | tubulin beta-7 chain | chr2:12644258-12645932 REVERSE LENGTH=449</td><td>AT2G29550.1</td><td>51 kDa</td><td>1</td><td>1</td><td>0</td><td>1</td><td>0</td><td>2</td><td>0</td><td>0</td><td>0</td><td>0</td></tr>
<tr><td>| Symbols: TUB4 | tubulin beta chain 4 | chr5:17859442-17860994 REVERSE LENGTH=444</td><td>AT5G44340.1</td><td>50 kDa</td><td>1</td><td>1</td><td>0</td><td>0</td><td>0</td><td>1</td><td>1</td><td>1</td><td>0</td><td>0</td></tr>
<tr><td>| Symbols: TUB8 | tubulin beta 8 | chr5:8042962-8044528 FORWARD LENGTH=449</td><td>AT5G23860.1 (+1)</td><td>51 kDa</td><td>0</td><td>0</td><td>0</td><td>1</td><td>0</td><td>0</td><td>0</td><td>0</td><td>0</td><td>0</td></tr>
<tr><td>| Symbols: PCH1 | F-box family protein | chr2:7074940-7079181 FORWARD LENGTH=778</td><td>AT2G16365.1 (+2)</td><td>89 kDa</td><td>9</td><td>9</td><td>9</td><td>14</td><td>11</td><td>16</td><td>9</td><td>9</td><td>11</td><td>11</td></tr>
<tr><td>| Symbols: GAPC-2, GAPC2 | glyceraldehyde-3-phosphate dehydrogenase C2 | chr1:4608465-4610494 REVERSE LENGTH=338</td><td>AT1G13440.1</td><td>37 kDa</td><td>2</td><td>0</td><td>1</td><td>1</td><td>1</td><td>1</td><td>0</td><td>0</td><td>0</td><td>0</td></tr>
<tr><td>| Symbols: GAPC, GAPC-1, GAPC1 | glyceraldehyde-3-phosphate dehydrogenase C subunit 1 | chr3:1081077-1083131 FORWARD LENGTH=338</td><td>AT3G04120.1</td><td>37 kDa</td><td>1</td><td>0</td><td>1</td><td>1</td><td>1</td><td>1</td><td>0</td><td>0</td><td>0</td><td>0</td></tr>
<tr><td>| Symbols: ATPA | ATP synthase subunit alpha | chrC:9938-11461 REVERSE LENGTH=507</td><td>ATCG00120.1</td><td>55 kDa</td><td>12</td><td>9</td><td>1</td><td>10</td><td>0</td><td>13</td><td>9</td><td>8</td><td>7</td><td>4</td></tr>
<tr><td>| Symbols: TUA4, TOR2 | tubulin alpha-4 chain | chr1:1356421-1358266 REVERSE LENGTH=450</td><td>AT1G04820.1 (+1)</td><td>50 kDa</td><td>0</td><td>1</td><td>1</td><td>1</td><td>1</td><td>1</td><td>0</td><td>0</td><td>0</td><td>0</td></tr>
<tr><td>| Symbols: TUA3 | tubulin alpha-3 | chr5:6682761-6684474 REVERSE LENGTH=450</td><td>AT5G19770.1 (+1)</td><td>50 kDa</td><td>1</td><td>0</td><td>2</td><td>2</td><td>1</td><td>2</td><td>0</td><td>0</td><td>0</td><td>0</td></tr>
<tr><td>| Symbols: TUA6 | Tubulin/FtsZ family protein | chr4:8548753-8550319 REVERSE LENGTH=427</td><td>AT4G14960.1</td><td>47 kDa</td><td>0</td><td>0</td><td>0</td><td>0</td><td>0</td><td>0</td><td>0</td><td>1</td><td>0</td><td>0</td></tr>
<tr><td>| Symbols: ACT8 | actin 8 | chr1:18216539-18217947 FORWARD LENGTH=377</td><td>AT1G49240.1 (+1)</td><td>42 kDa</td><td>2</td><td>2</td><td>1</td><td>2</td><td>1</td><td>2</td><td>2</td><td>2</td><td>2</td><td>2</td></tr>
<tr><td>| Symbols: ACT7 | actin 7 | chr5:3052809-3054220 FORWARD LENGTH=377</td><td>AT5G09810.1</td><td>42 kDa</td><td>2</td><td>2</td><td>0</td><td>2</td><td>1</td><td>2</td><td>2</td><td>1</td><td>0</td><td>0</td></tr>
<tr><td>| Symbols: ACT1, AAc1 | actin 1 | chr2:15779761-15781241 FORWARD LENGTH=377</td><td>AT2G37620.1 (+2)</td><td>42 kDa</td><td>1</td><td>0</td><td>0</td><td>1</td><td>0</td><td>0</td><td>0</td><td>0</td><td>0</td><td>0</td></tr>
<tr><td>| Symbols: ACT4 | actin 4 | chr5:23950109-23951586 FORWARD LENGTH=377</td><td>AT5G59370.1 (+1)</td><td>42 kDa</td><td>0</td><td>0</td><td>0</td><td>0</td><td>0</td><td>0</td><td>0</td><td>0</td><td>0</td><td>0</td></tr>
<tr><td>| Symbols: | Actin family protein | chr2:17578683-17580222 FORWARD LENGTH=329</td><td>AT2G42170.1</td><td>37 kDa</td><td>0</td><td>0</td><td>1</td><td>0</td><td>0</td><td>0</td><td>0</td><td>0</td><td>0</td><td>0</td></tr>
<tr><td>| Symbols: | Actin-like ATPase superfamily protein | chr2:17560211-17561945 FORWARD LENGTH=378</td><td>AT2G42100.1</td><td>42 kDa</td><td>0</td><td>0</td><td>0</td><td>0</td><td>0</td><td>0</td><td>0</td><td>0</td><td>0</td><td>0</td></tr>
<tr><td>| Symbols: ATRAB8D, ATRABE1B, RABE1b | RAB GTPase homolog E1B | chr4:10990036-10991466 FORWARD LENGTH=476</td><td>AT4G20360.1</td><td>52 kDa</td><td>6</td><td>5</td><td>9</td><td>10</td><td>8</td><td>12</td><td>5</td><td>4</td><td>3</td><td>3</td></tr>
<tr><td>| Symbols: | Ribosomal protein S5 family protein | chr2:17460016-17461398 REVERSE LENGTH=285</td><td>AT2G41840.1</td><td>31 kDa</td><td>2</td><td>3</td><td>0</td><td>1</td><td>0</td><td>1</td><td>2</td><td>2</td><td>1</td><td>1</td></tr>
<tr><td>| Symbols: XW6 | Ribosomal protein S5 family protein | chr1:21689115-21690085 FORWARD LENGTH=284</td><td>AT1G58380.1 (+3)</td><td>31 kDa</td><td>2</td><td>2</td><td>0</td><td>0</td><td>1</td><td>1</td><td>0</td><td>0</td><td>0</td><td>0</td></tr>
<tr><td>| Symbols: | Ribosomal protein S5 family protein | chr3:21279824-21280887 REVERSE LENGTH=276</td><td>AT3G57490.1</td><td>30 kDa</td><td>0</td><td>0</td><td>0</td><td>0</td><td>0</td><td>0</td><td>0</td><td>0</td><td>0</td><td>0</td></tr>
<tr><td>| Symbols: TIC | time for coffee | chr3:7913181-7918967 FORWARD LENGTH=1555</td><td>AT3G22380.2</td><td>165 kDa</td><td>5</td><td>5</td><td>4</td><td>12</td><td>3</td><td>15</td><td>4</td><td>3</td><td>1</td><td>1</td></tr>
<tr><td>| Symbols: ELF4 | Protein of unknown function (DUF1313) | chr2:16734545-16734880 REVERSE LENGTH=111</td><td>AT2G40080.1</td><td>12 kDa</td><td>4</td><td>4</td><td>3</td><td>5</td><td>4</td><td>4</td><td>4</td><td>4</td><td>5</td><td>5</td></tr>
<tr><td>| Symbols: GAPA, GAPA-1 | glyceraldehyde 3-phosphate dehydrogenase A subunit | chr3:9795226-9796848 FORWARD LENGTH=396</td><td>AT3G26650.1</td><td>42 kDa</td><td>3</td><td>2</td><td>0</td><td>4</td><td>0</td><td>5</td><td>2</td><td>2</td><td>2</td><td>1</td></tr>
<tr><td>| Symbols: GAPA-2 | glyceraldehyde 3-phosphate dehydrogenase A subunit 2 | chr1:4392634-4393850 REVERSE LENGTH=350</td><td>AT1G12900.3 (+1)</td><td>38 kDa</td><td>3</td><td>2</td><td>0</td><td>2</td><td>0</td><td>2</td><td>2</td><td>1</td><td>1</td><td>0</td></tr>
<tr><td>| Symbols: | Ribosomal protein L6 family | chr1:12010986-12012223 FORWARD LENGTH=194</td><td>AT1G33120.1 (+1)</td><td>22 kDa</td><td>3</td><td>2</td><td>5</td><td>7</td><td>5</td><td>6</td><td>3</td><td>3</td><td>3</td><td>3</td></tr>
<tr><td>| Symbols: | Ribosomal protein L6 family | chr4:6463201-6464458 REVERSE LENGTH=194</td><td>AT4G10450.1</td><td>22 kDa</td><td>0</td><td>0</td><td>0</td><td>0</td><td>0</td><td>0</td><td>0</td><td>0</td><td>0</td><td>0</td></tr>
<tr><td>| Symbols: COP1, ATCOP1, DET340, FUS1, EMB168 | Transducin/WD40 repeat-like superfamily protein | chr2:13978000-13983282 FORWARD LENGTH=675</td><td>AT2G32950.1</td><td>76 kDa</td><td>8</td><td>9</td><td>6</td><td>4</td><td>3</td><td>5</td><td>1</td><td>2</td><td>6</td><td>4</td></tr>
<tr><td>| Symbols: IMPA-2 | importin alpha isoform 2 | chr4:9134450-9137134 REVERSE LENGTH=535</td><td>AT4G16143.1 (+1)</td><td>59 kDa</td><td>2</td><td>2</td><td>1</td><td>6</td><td>0</td><td>7</td><td>8</td><td>8</td><td>2</td><td>4</td></tr>
<tr><td>| Symbols: AT-IMP, ATKAP ALPHA, AIMP ALPHA, IMPA-1, IMPA1 | importin alpha isoform 1 | chr3:2120559-2123555 FORWARD LENGTH=532</td><td>AT3G06720.1 (+1)</td><td>59 kDa</td><td>0</td><td>0</td><td>0</td><td>0</td><td>0</td><td>1</td><td>0</td><td>0</td><td>0</td><td>0</td></tr>
<tr><td>| Symbols: IMPA-6 | importin alpha isoform 6 | chr1:584397-587036 FORWARD LENGTH=538</td><td>AT1G02690.1 (+1)</td><td>59 kDa</td><td>0</td><td>0</td><td>0</td><td>0</td><td>0</td><td>0</td><td>0</td><td>0</td><td>0</td><td>0</td></tr>
<tr><td>| Symbols: IMPA-4 | importin alpha isoform 4 | chr1:2994506-2997833 FORWARD LENGTH=538</td><td>AT1G09270.1 (+2)</td><td>59 kDa</td><td>0</td><td>0</td><td>0</td><td>0</td><td>0</td><td>0</td><td>0</td><td>0</td><td>0</td><td>0</td></tr>
<tr><td>| Symbols: MTO3, SAMS3, MAT4 | S-adenosylmethionine synthetase family protein | chr3:5952484-5953665 REVERSE LENGTH=393</td><td>AT3G17390.1</td><td>43 kDa</td><td>5</td><td>5</td><td>0</td><td>3</td><td>0</td><td>3</td><td>1</td><td>0</td><td>0</td><td>0</td></tr>
<tr><td>| Symbols: MAT3 | methionine adenosyltransferase 3 | chr2:15479721-15480893 REVERSE LENGTH=390</td><td>AT2G36880.1 (+1)</td><td>42 kDa</td><td>0</td><td>0</td><td>0</td><td>0</td><td>0</td><td>0</td><td>0</td><td>0</td><td>0</td><td>0</td></tr>
<tr><td>| Symbols: SAM-2, MAT2, SAM2, AtSAM2 | S-adenosylmethionine synthetase 2 | chr4:796298-797479 REVERSE LENGTH=393</td><td>AT4G01850.1 (+1)</td><td>43 kDa</td><td>1</td><td>1</td><td>0</td><td>0</td><td>0</td><td>0</td><td>0</td><td>0</td><td>0</td><td>0</td></tr>
<tr><td>| Symbols: SAM1, SAM-1, MAT1, AtSAM1 | S-adenosylmethionine synthetase 1 | chr1:519037-520218 FORWARD LENGTH=393</td><td>AT1G02500.1 (+1)</td><td>43 kDa</td><td>0</td><td>0</td><td>0</td><td>0</td><td>0</td><td>0</td><td>0</td><td>0</td><td>0</td><td>0</td></tr>
<tr><td>| Symbols: RCA | rubisco activase | chr2:16570951-16573345 REVERSE LENGTH=474</td><td>AT2G39730.1</td><td>52 kDa</td><td>7</td><td>7</td><td>3</td><td>4</td><td>3</td><td>6</td><td>7</td><td>6</td><td>3</td><td>2</td></tr>
<tr><td>| Symbols: PCL1, LUX | Homeodomain-like superfamily protein | chr3:17183248-17184219 FORWARD LENGTH=323</td><td>AT3G46640.1 (+1)</td><td>35 kDa</td><td>1</td><td>1</td><td>0</td><td>3</td><td>0</td><td>3</td><td>1</td><td>1</td><td>1</td><td>2</td></tr>
<tr><td>| Symbols: GS2, GLN2, ATGSL1 | glutamine synthetase 2 | chr5:13831220-13833239 FORWARD LENGTH=430</td><td>AT5G35630.1 (+2)</td><td>47 kDa</td><td>6</td><td>5</td><td>3</td><td>8</td><td>3</td><td>6</td><td>5</td><td>4</td><td>1</td><td>1</td></tr>
<tr><td>| Symbols: CAT3, SEN2, ATCAT3 | catalase 3 | chr1:7143142-7146193 FORWARD LENGTH=485</td><td>AT1G20620.5</td><td>56 kDa</td><td>11</td><td>9</td><td>1</td><td>5</td><td>0</td><td>6</td><td>5</td><td>2</td><td>0</td><td>0</td></tr>
<tr><td>| Symbols: | Clathrin, heavy chain | chr3:3482575-3491667 REVERSE LENGTH=1705</td><td>AT3G11130.1</td><td>193 kDa</td><td>4</td><td>2</td><td>0</td><td>0</td><td>0</td><td>0</td><td>0</td><td>0</td><td>0</td><td>0</td></tr>
<tr><td>| Symbols: | Clathrin, heavy chain | chr3:2587171-2595411 REVERSE LENGTH=1703</td><td>AT3G08530.1</td><td>193 kDa</td><td>3</td><td>1</td><td>0</td><td>0</td><td>0</td><td>0</td><td>0</td><td>0</td><td>0</td><td>0</td></tr>
<tr><td>| Symbols: | Ribosomal protein S3 family protein | chr3:19951547-19952782 FORWARD LENGTH=249</td><td>AT3G53870.1</td><td>27 kDa</td><td>1</td><td>1</td><td>0</td><td>0</td><td>0</td><td>0</td><td>1</td><td>1</td><td>1</td><td>0</td></tr>
<tr><td>| Symbols: | Ribosomal protein S3 family protein | chr2:13450384-13451669 FORWARD LENGTH=250</td><td>AT2G31610.1</td><td>28 kDa</td><td>1</td><td>1</td><td>0</td><td>0</td><td>0</td><td>1</td><td>1</td><td>1</td><td>0</td><td>0</td></tr>
<tr><td>| Symbols: | Ribosomal protein S3 family protein | chr5:13710355-13712192 REVERSE LENGTH=248</td><td>AT5G35530.1</td><td>27 kDa</td><td>1</td><td>1</td><td>0</td><td>1</td><td>0</td><td>1</td><td>1</td><td>0</td><td>0</td><td>0</td></tr>
<tr><td>| Symbols: EIF4A1, RH4, TIF4A1 | eukaryotic translation initiation factor 4A1 | chr3:4592635-4594128 REVERSE LENGTH=412</td><td>AT3G13920.1 (+1)</td><td>47 kDa</td><td>2</td><td>2</td><td>0</td><td>0</td><td>0</td><td>2</td><td>0</td><td>0</td><td>0</td><td>0</td></tr>
<tr><td>| Symbols: EIF4A-2 | eif4a-2 | chr1:20260495-20262018 FORWARD LENGTH=412</td><td>AT1G54270.1</td><td>47 kDa</td><td>0</td><td>0</td><td>0</td><td>0</td><td>0</td><td>2</td><td>0</td><td>0</td><td>0</td><td>0</td></tr>
<tr><td>| Symbols: EIF4A-III | eukaryotic initiation factor 4A-III | chr3:6863790-6866242 FORWARD LENGTH=408</td><td>AT3G19760.1</td><td>?</td><td>0</td><td>0</td><td>0</td><td>0</td><td>0</td><td>0</td><td>0</td><td>0</td><td>0</td><td>0</td></tr>
<tr><td>| Symbols: BIP, BIP2 | Heat shock protein 70 (Hsp 70) family protein | chr5:16807697-16810480 REVERSE LENGTH=668</td><td>AT5G42020.1</td><td>74 kDa</td><td>2</td><td>4</td><td>3</td><td>7</td><td>5</td><td>9</td><td>0</td><td>1</td><td>2</td><td>1</td></tr>
<tr><td>| Symbols: DAYSLEEPER | BED zinc finger ;hAT family dimerisation domain | chr3:14321838-14323928 FORWARD LENGTH=696</td><td>AT3G42170.1</td><td>79 kDa</td><td>8</td><td>5</td><td>0</td><td>7</td><td>0</td><td>12</td><td>3</td><td>4</td><td>2</td><td>0</td></tr>
<tr><td>MLK2 | Symbols: | Protein kinase family protein | chr3:1014412-1018244 REVERSE LENGTH=701</td><td>AT3G03940.1</td><td>78 kDa</td><td>1</td><td>1</td><td>0</td><td>1</td><td>0</td><td>0</td><td>3</td><td>2</td><td>3</td><td>1</td></tr>
<tr><td>MLK1 | Symbols: | Protein kinase family protein | chr5:6010215-6013724 REVERSE LENGTH=691</td><td>AT5G18190.1</td><td>77 kDa</td><td>2</td><td>2</td><td>0</td><td>0</td><td>0</td><td>0</td><td>2</td><td>2</td><td>2</td><td>2</td></tr>
<tr><td>| Symbols: GAPB | glyceraldehyde-3-phosphate dehydrogenase B subunit | chr1:16127552-16129584 FORWARD LENGTH=447</td><td>AT1G42970.1</td><td>48 kDa</td><td>7</td><td>6</td><td>0</td><td>9</td><td>0</td><td>13</td><td>4</td><td>3</td><td>2</td><td>0</td></tr>
<tr><td>| Symbols: SPA1 | SPA (suppressor of phyA-105) protein family | chr2:19022572-19026821 REVERSE LENGTH=1029</td><td>AT2G46340.1</td><td>115 kDa</td><td>8</td><td>7</td><td>2</td><td>4</td><td>2</td><td>4</td><td>0</td><td>0</td><td>6</td><td>5</td></tr>
<tr><td>| Symbols: | Ribulose bisphosphate carboxylase (small chain) family protein | chr5:15377501-15378306 REVERSE LENGTH=181</td><td>AT5G38410.1</td><td>20 kDa</td><td>2</td><td>2</td><td>2</td><td>4</td><td>3</td><td>4</td><td>2</td><td>2</td><td>1</td><td>1</td></tr>
<tr><td>| Symbols: | Ribulose bisphosphate carboxylase (small chain) family protein | chr5:15384350-15385155 REVERSE LENGTH=181</td><td>AT5G38430.1</td><td>20 kDa</td><td>1</td><td>1</td><td>0</td><td>0</td><td>0</td><td>0</td><td>0</td><td>0</td><td>0</td><td>0</td></tr>
<tr><td>| Symbols: PHYA, FHY2, FRE1, HY8 | phytochrome A | chr1:3095498-3099216 REVERSE LENGTH=1122</td><td>AT1G09570.1</td><td>125 kDa</td><td>1</td><td>1</td><td>7</td><td>5</td><td>7</td><td>2</td><td>0</td><td>2</td><td>6</td><td>3</td></tr>
<tr><td>| Symbols: | Ribosomal protein L4/L1 family | chr3:2953813-2955444 FORWARD LENGTH=406</td><td>AT3G09630.1 (+1)</td><td>45 kDa</td><td>1</td><td>1</td><td>0</td><td>1</td><td>0</td><td>2</td><td>1</td><td>1</td><td>1</td><td>0</td></tr>
<tr><td>| Symbols: | Ribosomal protein L4/L1 family | chr5:657830-659526 FORWARD LENGTH=407</td><td>AT5G02870.1 (+1)</td><td>45 kDa</td><td>2</td><td>2</td><td>0</td><td>1</td><td>0</td><td>1</td><td>1</td><td>1</td><td>0</td><td>0</td></tr>
<tr><td>| Symbols: P5CS1, ATP5CS | delta1-pyrroline-5-carboxylate synthase 1 | chr2:16598516-16602939 REVERSE LENGTH=717</td><td>AT2G39800.1 (+1)</td><td>78 kDa</td><td>7</td><td>6</td><td>1</td><td>4</td><td>1</td><td>9</td><td>4</td><td>3</td><td>2</td><td>0</td></tr>
<tr><td>| Symbols: P5CS2 | delta 1-pyrroline-5-carboxylate synthase 2 | chr3:20624278-20628989 REVERSE LENGTH=726</td><td>AT3G55610.1</td><td>79 kDa</td><td>0</td><td>0</td><td>0</td><td>0</td><td>0</td><td>0</td><td>0</td><td>0</td><td>0</td><td>0</td></tr>
<tr><td>| Symbols: RBCS1A | ribulose bisphosphate carboxylase small chain 1A | chr1:25048465-25049249 REVERSE LENGTH=180</td><td>AT1G67090.1</td><td>20 kDa</td><td>3</td><td>3</td><td>2</td><td>5</td><td>3</td><td>5</td><td>2</td><td>2</td><td>0</td><td>2</td></tr>
<tr><td>| Symbols: ATCIMS, ATMETS, ATMS1 | Cobalamin-independent synthase family protein | chr5:5935771-5939195 FORWARD LENGTH=765</td><td>AT5G17920.1 (+1)</td><td>84 kDa</td><td>2</td><td>3</td><td>2</td><td>4</td><td>2</td><td>5</td><td>1</td><td>1</td><td>0</td><td>0</td></tr>
<tr><td>| Symbols: ATMS3, MS3 | methionine synthase 3 | chr5:7124397-7128353 REVERSE LENGTH=812</td><td>AT5G20980.1 (+1)</td><td>91 kDa</td><td>0</td><td>0</td><td>0</td><td>0</td><td>0</td><td>0</td><td>0</td><td>0</td><td>0</td><td>0</td></tr>
<tr><td>| Symbols: PGK1 | phosphoglycerate kinase 1 | chr3:4061127-4063140 REVERSE LENGTH=481</td><td>AT3G12780.1</td><td>50 kDa</td><td>2</td><td>2</td><td>0</td><td>3</td><td>0</td><td>5</td><td>2</td><td>1</td><td>0</td><td>0</td></tr>
<tr><td>| Symbols: PGK | phosphoglycerate kinase | chr1:29924347-29926295 REVERSE LENGTH=401</td><td>AT1G79550.1 (+1)</td><td>42 kDa</td><td>1</td><td>0</td><td>0</td><td>0</td><td>0</td><td>0</td><td>0</td><td>0</td><td>0</td><td>0</td></tr>
<tr><td>| Symbols: | Phosphoglycerate kinase family protein | chr1:21028622-21030454 FORWARD LENGTH=405</td><td>AT1G56190.2</td><td>43 kDa</td><td>0</td><td>0</td><td>0</td><td>0</td><td>0</td><td>0</td><td>0</td><td>0</td><td>0</td><td>0</td></tr>
<tr><td>| Symbols: LOS1 | Ribosomal protein S5/Elongation factor G/III/V family protein | chr1:20968245-20971077 REVERSE LENGTH=843</td><td>AT1G56070.1</td><td>94 kDa</td><td>4</td><td>3</td><td>1</td><td>3</td><td>2</td><td>9</td><td>1</td><td>1</td><td>0</td><td>0</td></tr>
<tr><td>| Symbols: | structural constituent of ribosome | chrC:82826-83482 REVERSE LENGTH=218</td><td>ATCG00800.1</td><td>25 kDa</td><td>6</td><td>4</td><td>1</td><td>4</td><td>1</td><td>6</td><td>1</td><td>0</td><td>0</td><td>0</td></tr>
<tr><td>| Symbols: CAT2 | catalase 2 | chr4:16700937-16703215 REVERSE LENGTH=492</td><td>AT4G35090.1</td><td>57 kDa</td><td>8</td><td>4</td><td>1</td><td>5</td><td>0</td><td>4</td><td>2</td><td>2</td><td>0</td><td>0</td></tr>
<tr><td>| Symbols: | Ribosomal protein S27a / Ubiquitin family protein | chr1:8314940-8315410 FORWARD LENGTH=156</td><td>AT1G23410.1 (+3)</td><td>18 kDa</td><td>1</td><td>1</td><td>0</td><td>1</td><td>0</td><td>1</td><td>1</td><td>0</td><td>0</td><td>0</td></tr>
<tr><td>| Symbols: UBQ9 | ubiquitin 9 | chr5:14952782-14953750 REVERSE LENGTH=322</td><td>AT5G37640.1</td><td>36 kDa</td><td>0</td><td>0</td><td>0</td><td>0</td><td>0</td><td>0</td><td>0</td><td>0</td><td>0</td><td>0</td></tr>
<tr><td>| Symbols: UBQ12 | ubiquitin 12 | chr1:20549533-20550225 FORWARD LENGTH=230</td><td>AT1G55060.1</td><td>26 kDa</td><td>0</td><td>0</td><td>0</td><td>0</td><td>0</td><td>0</td><td>0</td><td>0</td><td>0</td><td>0</td></tr>
<tr><td>| Symbols: UBQ8 | ubiquitin 8 | chr3:3004111-3006006 REVERSE LENGTH=631</td><td>AT3G09790.1</td><td>72 kDa</td><td>0</td><td>0</td><td>0</td><td>0</td><td>0</td><td>0</td><td>0</td><td>0</td><td>0</td><td>0</td></tr>
<tr><td>| Symbols: | GTP binding Elongation factor Tu family protein | chr1:2455559-2457001 FORWARD LENGTH=449</td><td>AT1G07920.1 (+5)</td><td>50 kDa</td><td>4</td><td>3</td><td>1</td><td>4</td><td>1</td><td>6</td><td>3</td><td>1</td><td>2</td><td>1</td></tr>
<tr><td>| Symbols: | Ribosomal protein L13 family protein | chr3:9064613-9065871 FORWARD LENGTH=206</td><td>AT3G24830.1</td><td>23 kDa</td><td>1</td><td>1</td><td>0</td><td>0</td><td>1</td><td>0</td><td>1</td><td>1</td><td>0</td><td>0</td></tr>
<tr><td>| Symbols: | Ribosomal protein L13 family protein | chr3:2252092-2253332 FORWARD LENGTH=206</td><td>AT3G07110.1 (+1)</td><td>23 kDa</td><td>1</td><td>1</td><td>0</td><td>0</td><td>0</td><td>0</td><td>1</td><td>1</td><td>0</td><td>0</td></tr>
<tr><td>| Symbols: | Ribosomal protein L13 family protein | chr5:19771315-19772686 REVERSE LENGTH=206</td><td>AT5G48760.1 (+1)</td><td>24 kDa</td><td>0</td><td>0</td><td>0</td><td>0</td><td>0</td><td>0</td><td>1</td><td>0</td><td>0</td><td>0</td></tr>
<tr><td>| Symbols: | Ribosomal protein L13 family protein | chr4:7655133-7656542 REVERSE LENGTH=206</td><td>AT4G13170.1</td><td>24 kDa</td><td>0</td><td>0</td><td>0</td><td>0</td><td>0</td><td>0</td><td>0</td><td>0</td><td>0</td><td>0</td></tr>
<tr><td>| Symbols: HOG1, EMB1395, SAHH1, MEE58, ATSAHH1 | S-adenosyl-L-homocysteine hydrolase | chr4:8054931-8056676 FORWARD LENGTH=485</td><td>AT4G13940.1</td><td>53 kDa</td><td>1</td><td>2</td><td>0</td><td>2</td><td>0</td><td>2</td><td>1</td><td>1</td><td>0</td><td>0</td></tr>
<tr><td>| Symbols: SAHH2, ATSAHH2 | S-adenosyl-l-homocysteine (SAH) hydrolase 2 | chr3:8588013-8589671 REVERSE LENGTH=485</td><td>AT3G23810.1</td><td>53 kDa</td><td>0</td><td>0</td><td>0</td><td>0</td><td>0</td><td>0</td><td>0</td><td>0</td><td>0</td><td>0</td></tr>
<tr><td>| Symbols: HOG1, SAHH1 | S-adenosyl-L-homocysteine hydrolase | chr4:8054931-8056763 FORWARD LENGTH=325</td><td>AT4G13940.4</td><td>36 kDa</td><td>0</td><td>0</td><td>0</td><td>0</td><td>0</td><td>0</td><td>0</td><td>0</td><td>0</td><td>0</td></tr>
<tr><td>| Symbols: CRB, CSP41B, HIP1.3 | chloroplast RNA binding | chr1:3015473-3018035 FORWARD LENGTH=378</td><td>AT1G09340.1</td><td>43 kDa</td><td>4</td><td>3</td><td>1</td><td>1</td><td>1</td><td>4</td><td>1</td><td>1</td><td>0</td><td>0</td></tr>
<tr><td>| Symbols: ACC1, AT-ACC1, EMB22, GK, PAS3 | acetyl-CoA carboxylase 1 | chr1:13534196-13543773 FORWARD LENGTH=2254</td><td>AT1G36160.1 (+1)</td><td>251 kDa</td><td>12</td><td>1</td><td>0</td><td>1</td><td>0</td><td>2</td><td>0</td><td>0</td><td>0</td><td>0</td></tr>
<tr><td>| Symbols: | Ribosomal protein S5 domain 2-like superfamily protein | chr5:6090253-6090693 REVERSE LENGTH=146</td><td>AT5G18380.1 (+1)</td><td>17 kDa</td><td>1</td><td>1</td><td>1</td><td>1</td><td>1</td><td>1</td><td>1</td><td>1</td><td>0</td><td>0</td></tr>
<tr><td>| Symbols: | Ribosomal protein S5 domain 2-like superfamily protein | chr2:3781442-3781882 FORWARD LENGTH=146</td><td>AT2G09990.1</td><td>17 kDa</td><td>0</td><td>0</td><td>0</td><td>0</td><td>0</td><td>0</td><td>0</td><td>0</td><td>0</td><td>0</td></tr>
</table>

<table>
<tr><td>| Symbols: CA1 | carbonic anhydrase 1 | chr3:194853-197873 REVERSE LENGTH=347</td><td>AT3G01500.2 (+1)</td><td>37 kDa</td><td>1</td><td>1</td><td>0</td><td>1</td><td>0</td><td>1</td><td>2</td><td>1</td><td>0</td><td>0</td></tr>
<tr><td>| Symbols: CA2, CA18, BETA CA2 | carbonic anhydrase 2 | chr5:4758257-4762382 FORWARD LENGTH=331</td><td>AT5G14740.1 (+1)</td><td>37 kDa</td><td>0</td><td>0</td><td>0</td><td>1</td><td>0</td><td>2</td><td>0</td><td>0</td><td>0</td><td>0</td></tr>
<tr><td>| Symbols: ATAN11, LWD1 | Transducin/WD40 repeat-like superfamily protein | chr1:4395114-4396154 REVERSE LENGTH=346</td><td>AT1G12910.1</td><td>39 kDa</td><td>2</td><td>3</td><td>2</td><td>4</td><td>1</td><td>2</td><td>2</td><td>2</td><td>0</td><td>0</td></tr>
<tr><td>| Symbols: MLK4 | Protein kinase family protein | chr3:4469434-4473234 FORWARD LENGTH=703</td><td>AT3G13670.1</td><td>79 kDa</td><td>3</td><td>3</td><td>0</td><td>0</td><td>0</td><td>1</td><td>6</td><td>3</td><td>3</td><td>5</td></tr>
<tr><td>| Symbols: RPS6, RPS6A | ribosomal protein S6 | chr4:15346306-15347714 REVERSE LENGTH=250</td><td>AT4G31700.1</td><td>28 kDa</td><td>2</td><td>4</td><td>0</td><td>1</td><td>0</td><td>4</td><td>4</td><td>4</td><td>2</td><td>1</td></tr>
<tr><td>| Symbols: | Ribosomal protein L30/L7 family protein | chr2:132943-134264 REVERSE LENGTH=242</td><td>AT2G01250.1</td><td>28 kDa</td><td>3</td><td>2</td><td>0</td><td>0</td><td>0</td><td>1</td><td>1</td><td>1</td><td>1</td><td>0</td></tr>
<tr><td>| Symbols: | Ribosomal protein L30/L7 family protein | chr2:18249227-18250417 REVERSE LENGTH=247</td><td>AT2G44120.2</td><td>29 kDa</td><td>1</td><td>1</td><td>0</td><td>1</td><td>0</td><td>1</td><td>0</td><td>0</td><td>0</td><td>0</td></tr>
<tr><td>| Symbols: | Ribosomal protein L30/L7 family protein | chr3:4433809-4435109 FORWARD LENGTH=244</td><td>AT3G13580.1 (+2)</td><td>28 kDa</td><td>1</td><td>1</td><td>0</td><td>0</td><td>0</td><td>0</td><td>1</td><td>0</td><td>0</td><td>0</td></tr>
<tr><td>| Symbols: | Coatomer, alpha subunit | chr2:9152428-9156577 FORWARD LENGTH=1218</td><td>AT2G21390.1</td><td>136 kDa</td><td>2</td><td>0</td><td>0</td><td>2</td><td>0</td><td>5</td><td>0</td><td>0</td><td>0</td><td>0</td></tr>
<tr><td>| Symbols: | Coatomer, alpha subunit | chr1:22919814-22923728 FORWARD LENGTH=1216</td><td>AT1G62020.1</td><td>137 kDa</td><td>2</td><td>1</td><td>0</td><td>0</td><td>0</td><td>1</td><td>0</td><td>0</td><td>0</td><td>0</td></tr>
<tr><td>| Symbols: CAB3, AB180, LHCB1.2 | chlorophyll A/B binding protein 3 | chr1:10472443-10473246 REVERSE LENGTH=267</td><td>AT1G29910.1 (+1)</td><td>28 kDa</td><td>0</td><td>0</td><td>0</td><td>0</td><td>0</td><td>0</td><td>0</td><td>0</td><td>0</td><td>0</td></tr>
<tr><td>| Symbols: LHCB2.2, LHCB2 | photosystem II light harvesting complex gene 2.2 | chr2:1799436-1800329 REVERSE LENGTH=265</td><td>AT2G05070.1 (+2)</td><td>29 kDa</td><td>0</td><td>0</td><td>1</td><td>0</td><td>0</td><td>0</td><td>0</td><td>0</td><td>0</td><td>0</td></tr>
<tr><td>| Symbols: CAB1, AB140, CAB140, LHCB1.3 | chlorophyll A/B binding protein 1 | chr1:10478071-10478874 FORWARD LENGTH=267</td><td>AT1G29930.1</td><td>28 kDa</td><td>0</td><td>0</td><td>0</td><td>0</td><td>0</td><td>0</td><td>0</td><td>0</td><td>0</td><td>0</td></tr>
<tr><td>| Symbols: HSP60-2 | heat shock protein 60-2 | chr2:14075093-14078568 REVERSE LENGTH=585</td><td>AT2G33210.1 (+1)</td><td>62 kDa</td><td>1</td><td>1</td><td>1</td><td>1</td><td>1</td><td>0</td><td>1</td><td>0</td><td>0</td><td>0</td></tr>
<tr><td>| Symbols: HSP60, HSP60-3B | heat shock protein 60 | chr3:8669013-8672278 FORWARD LENGTH=577</td><td>AT3G23990.1</td><td>61 kDa</td><td>1</td><td>1</td><td>0</td><td>1</td><td>0</td><td>1</td><td>2</td><td>1</td><td>0</td><td>0</td></tr>
<tr><td>| Symbols: | Ribosomal protein S3Ae | chr3:1329751-1331418 FORWARD LENGTH=262</td><td>AT3G04840.1</td><td>30 kDa</td><td>1</td><td>1</td><td>0</td><td>0</td><td>0</td><td>1</td><td>1</td><td>1</td><td>1</td><td>0</td></tr>
<tr><td>| Symbols: | Ribosomal protein S3Ae | chr4:16548724-16550222 FORWARD LENGTH=262</td><td>AT4G34670.1</td><td>30 kDa</td><td>1</td><td>1</td><td>0</td><td>1</td><td>0</td><td>1</td><td>1</td><td>0</td><td>0</td><td>0</td></tr>
<tr><td>| Symbols: UBP12 | ubiquitin-specific protease 12 | chr5:2019545-2027834 REVERSE LENGTH=1116</td><td>AT5G06600.1 (+1)</td><td>131 kDa</td><td>0</td><td>0</td><td>0</td><td>1</td><td>0</td><td>1</td><td>0</td><td>1</td><td>0</td><td>0</td></tr>
<tr><td>| Symbols: UBP13 | ubiquitin-specific protease 13 | chr3:3761758-3770290 REVERSE LENGTH=1115</td><td>AT3G11910.1 (+1)</td><td>131 kDa</td><td>0</td><td>0</td><td>0</td><td>1</td><td>1</td><td>0</td><td>0</td><td>0</td><td>0</td><td>0</td></tr>
<tr><td>| Symbols: NIT1, ATNIT1, NITI | nitrilase 1 | chr3:15986901-15988841 FORWARD LENGTH=346</td><td>AT3G44310.1 (+1)</td><td>38 kDa</td><td>2</td><td>1</td><td>0</td><td>4</td><td>1</td><td>4</td><td>1</td><td>1</td><td>1</td><td>1</td></tr>
<tr><td>| Symbols: ARP1, emb2207, RPL3A, RP1 | ribosomal protein 1 | chr1:16266992-16268631 FORWARD LENGTH=389</td><td>AT1G43170.1 (+7)</td><td>45 kDa</td><td>2</td><td>1</td><td>0</td><td>2</td><td>0</td><td>1</td><td>1</td><td>1</td><td>0</td><td>0</td></tr>
<tr><td>| Symbols: RD21, RD21A | Granulin repeat cysteine protease family protein | chr1:17283139-17285609 REVERSE LENGTH=462</td><td>AT1G47128.1</td><td>51 kDa</td><td>4</td><td>3</td><td>0</td><td>2</td><td>0</td><td>2</td><td>0</td><td>2</td><td>0</td><td>0</td></tr>
<tr><td>| Symbols: ATPB, PB | ATP synthase subunit beta | chrC:52660-54156 REVERSE LENGTH=498</td><td>ATCG00480.1</td><td>54 kDa</td><td>4</td><td>5</td><td>0</td><td>2</td><td>0</td><td>2</td><td>2</td><td>2</td><td>1</td><td>0</td></tr>
<tr><td>| Symbols: PYK10, PSR3.1, BGLU23, LEB | Glycosyl hydrolase superfamily protein | chr3:2840657-2843730 REVERSE LENGTH=524</td><td>AT3G09260.1</td><td>?</td><td>1</td><td>1</td><td>1</td><td>3</td><td>0</td><td>3</td><td>1</td><td>1</td><td>0</td><td>0</td></tr>
<tr><td>| Symbols: KASI, KAS1 | 3-ketoacyl-acyl carrier protein synthase I | chr5:18774439-18776629 REVERSE LENGTH=473</td><td>AT5G46290.1</td><td>50 kDa</td><td>4</td><td>3</td><td>0</td><td>1</td><td>2</td><td>3</td><td>2</td><td>2</td><td>0</td><td>0</td></tr>
<tr><td>| Symbols: RPL2.1 | ribosomal protein L2 | chrC:84337-85843 REVERSE LENGTH=274</td><td>ATCG00830.1 (+1)</td><td>30 kDa</td><td>3</td><td>2</td><td>0</td><td>2</td><td>0</td><td>3</td><td>3</td><td>4</td><td>0</td><td>0</td></tr>
<tr><td>| Symbols: ASN2 | asparagine synthetase 2 | chr5:25969224-25972278 FORWARD LENGTH=578</td><td>AT5G65010.1 (+1)</td><td>65 kDa</td><td>2</td><td>1</td><td>0</td><td>1</td><td>0</td><td>3</td><td>1</td><td>1</td><td>0</td><td>0</td></tr>
<tr><td>| Symbols: ASN1, DIN6, AT-ASN1 | glutamine-dependent asparagine synthase 1 | chr3:17438445-17441043 REVERSE LENGTH=512</td><td>AT3G47340.2</td><td>58 kDa</td><td>0</td><td>0</td><td>0</td><td>0</td><td>0</td><td>0</td><td>0</td><td>0</td><td>0</td><td>0</td></tr>
<tr><td>| Symbols: ASN3 | asparagine synthetase 3 | chr5:3212934-3216418 REVERSE LENGTH=578</td><td>AT5G10240.1 (+1)</td><td>65 kDa</td><td>0</td><td>0</td><td>0</td><td>0</td><td>0</td><td>0</td><td>0</td><td>0</td><td>0</td><td>0</td></tr>
<tr><td>| Symbols: PIP2A, PIP2, PIP2;1 | plasma membrane intrinsic protein 2A | chr3:19803906-19805454 REVERSE LENGTH=287</td><td>AT3G53420.1 (+1)</td><td>30 kDa</td><td>4</td><td>4</td><td>0</td><td>1</td><td>0</td><td>1</td><td>2</td><td>1</td><td>0</td><td>0</td></tr>
<tr><td>| Symbols: | Ribosomal protein S10p/S20e family protein | chr3:16471606-16472312 REVERSE LENGTH=124</td><td>AT3G45030.1 (+2)</td><td>14 kDa</td><td>1</td><td>0</td><td>0</td><td>0</td><td>0</td><td>1</td><td>0</td><td>0</td><td>0</td><td>0</td></tr>
<tr><td>| Symbols: | Ribosomal protein S10p/S20e family protein | chr3:17453671-17454437 REVERSE LENGTH=122</td><td>AT3G47370.1 (+2)</td><td>14 kDa</td><td>0</td><td>0</td><td>0</td><td>0</td><td>0</td><td>0</td><td>0</td><td>0</td><td>0</td><td>0</td></tr>
<tr><td>| Symbols: | Coatomer, beta subunit | chr4:15269460-15272693 FORWARD LENGTH=948</td><td>AT4G31490.1</td><td>106 kDa</td><td>0</td><td>0</td><td>0</td><td>0</td><td>0</td><td>1</td><td>0</td><td>0</td><td>0</td><td>0</td></tr>
<tr><td>| Symbols: | Coatomer, beta subunit | chr4:15264145-15267384 FORWARD LENGTH=948</td><td>AT4G31480.1 (+1)</td><td>106 kDa</td><td>0</td><td>0</td><td>0</td><td>0</td><td>0</td><td>0</td><td>0</td><td>0</td><td>0</td><td>0</td></tr>
<tr><td>| Symbols: MLK3 | Protein kinase family protein | chr2:10985118-10988652 REVERSE LENGTH=673</td><td>AT2G25760.1</td><td>76 kDa</td><td>1</td><td>1</td><td>0</td><td>1</td><td>0</td><td>1</td><td>3</td><td>2</td><td>2</td><td>2</td></tr>
<tr><td>| Symbols: RPT3 | regulatory particle triple-A ATPase 3 | chr5:23569155-23571116 FORWARD LENGTH=408</td><td>AT5G58290.1</td><td>46 kDa</td><td>2</td><td>2</td><td>1</td><td>1</td><td>1</td><td>2</td><td>0</td><td>0</td><td>0</td><td>0</td></tr>
<tr><td>| Symbols: CPN60B, LEN1 | chaperonin 60 beta | chr1:20715717-20718673 REVERSE LENGTH=600</td><td>AT1G55490.1 (+1)</td><td>64 kDa</td><td>1</td><td>2</td><td>0</td><td>1</td><td>0</td><td>1</td><td>1</td><td>0</td><td>0</td><td>0</td></tr>
<tr><td>| Symbols: | TCP-1/cpn60 chaperonin family protein | chr5:22874058-22876966 FORWARD LENGTH=597</td><td>AT5G56500.1 (+1)</td><td>63 kDa</td><td>0</td><td>0</td><td>0</td><td>0</td><td>0</td><td>0</td><td>0</td><td>0</td><td>0</td><td>0</td></tr>
<tr><td>| Symbols: ATARCA, RACK1A_AT, RACK1A | Transducin/WD40 repeat-like superfamily protein | chr1:6222325-6223901 FORWARD LENGTH=327</td><td>AT1G18080.1</td><td>36 kDa</td><td>0</td><td>1</td><td>0</td><td>2</td><td>0</td><td>2</td><td>1</td><td>0</td><td>0</td><td>0</td></tr>
<tr><td>| Symbols: RACK1B_AT | receptor for activated C kinase 1B | chr1:17981977-17983268 REVERSE LENGTH=326</td><td>AT1G48630.1</td><td>36 kDa</td><td>1</td><td>0</td><td>0</td><td>0</td><td>0</td><td>0</td><td>0</td><td>1</td><td>0</td><td>0</td></tr>
<tr><td>| Symbols: RACK1C_AT | receptor for activated C kinase 1C | chr3:6211109-6212371 REVERSE LENGTH=326</td><td>AT3G18130.1</td><td>36 kDa</td><td>0</td><td>0</td><td>0</td><td>0</td><td>0</td><td>0</td><td>0</td><td>0</td><td>0</td><td>0</td></tr>
<tr><td>| Symbols: AHA2, PMA2, HA2 | H(+)-ATPase 2 | chr4:14770820-14775920 REVERSE LENGTH=948</td><td>AT4G30190.1 (+1)</td><td>104 kDa</td><td>0</td><td>1</td><td>0</td><td>0</td><td>0</td><td>0</td><td>0</td><td>0</td><td>0</td><td>0</td></tr>
<tr><td>| Symbols: AHA1, PMA, OST2, HA1 | H(+)-ATPase 1 | chr2:8221858-8227268 FORWARD LENGTH=949</td><td>AT2G18960.1</td><td>104 kDa</td><td>0</td><td>0</td><td>0</td><td>0</td><td>0</td><td>0</td><td>0</td><td>0</td><td>0</td><td>0</td></tr>
<tr><td>| Symbols: AHA7, HA7 | H(+)-ATPase 7 | chr3:22298763-22303509 FORWARD LENGTH=961</td><td>AT3G60330.1 (+1)</td><td>106 kDa</td><td>0</td><td>0</td><td>0</td><td>0</td><td>0</td><td>0</td><td>0</td><td>0</td><td>0</td><td>0</td></tr>
<tr><td>| Symbols: AHA4, HA4 | H(+)-ATPase 4 | chr3:17693015-17697801 FORWARD LENGTH=960</td><td>AT3G47950.1</td><td>106 kDa</td><td>0</td><td>0</td><td>0</td><td>0</td><td>0</td><td>0</td><td>0</td><td>0</td><td>0</td><td>0</td></tr>
<tr><td>| Symbols: AHA3, ATAHA3, HA3 | H(+)-ATPase 3 | chr5:23231208-23236381 REVERSE LENGTH=949</td><td>AT5G57350.1 (+1)</td><td>104 kDa</td><td>0</td><td>0</td><td>0</td><td>0</td><td>0</td><td>0</td><td>0</td><td>0</td><td>0</td><td>0</td></tr>
<tr><td>| Symbols: AHA10 | autoinhibited H(+)-ATPase isoform 10 | chr1:5904058-5908898 FORWARD LENGTH=947</td><td>AT1G17260.1</td><td>105 kDa</td><td>0</td><td>0</td><td>0</td><td>0</td><td>0</td><td>0</td><td>0</td><td>0</td><td>0</td><td>0</td></tr>
<tr><td>| Symbols: | ATPase, F1 complex, alpha subunit protein | chr2:3361474-3364028 FORWARD LENGTH=777</td><td>AT2G07698.1</td><td>86 kDa</td><td>2</td><td>2</td><td>1</td><td>3</td><td>1</td><td>4</td><td>1</td><td>0</td><td>0</td><td>0</td></tr>
<tr><td>| Symbols: LWD2 | Transducin/WD40 repeat-like superfamily protein | chr3:9793276-9794316 FORWARD LENGTH=346</td><td>AT3G26640.1</td><td>39 kDa</td><td>2</td><td>1</td><td>0</td><td>1</td><td>0</td><td>2</td><td>3</td><td>1</td><td>0</td><td>0</td></tr>
<tr><td>| Symbols: | ATPase, V1 complex, subunit B protein | chr1:7016971-7020290 FORWARD LENGTH=487</td><td>AT1G20260.1 (+2)</td><td>54 kDa</td><td>3</td><td>3</td><td>0</td><td>2</td><td>0</td><td>2</td><td>1</td><td>2</td><td>0</td><td>0</td></tr>
<tr><td>| Symbols: ATGSTF8, ATGSTF5, GST6, GSTF8 | glutathione S-transferase phi 8 | chr2:19558213-19559266 FORWARD LENGTH=263</td><td>AT2G47730.1</td><td>29 kDa</td><td>3</td><td>2</td><td>0</td><td>0</td><td>0</td><td>4</td><td>0</td><td>0</td><td>0</td><td>0</td></tr>
<tr><td>| Symbols: ATPC1 | ATPase, F1 complex, gamma subunit protein | chr4:2350761-2351882 REVERSE LENGTH=373</td><td>AT4G04640.1</td><td>41 kDa</td><td>5</td><td>3</td><td>0</td><td>2</td><td>1</td><td>2</td><td>1</td><td>2</td><td>1</td><td>0</td></tr>
<tr><td>| Symbols: FBA2 | fructose-bisphosphate aldolase 2 | chr4:18163714-18165659 REVERSE LENGTH=398</td><td>AT4G38970.1</td><td>43 kDa</td><td>2</td><td>2</td><td>0</td><td>3</td><td>1</td><td>3</td><td>2</td><td>1</td><td>0</td><td>0</td></tr>
<tr><td>| Symbols: RPL16A | ribosomal protein large subunit 16A | chr2:17791794-17792946 FORWARD LENGTH=182</td><td>AT2G42740.1 (+4)</td><td>21 kDa</td><td>2</td><td>2</td><td>1</td><td>1</td><td>1</td><td>1</td><td>1</td><td>2</td><td>2</td><td>1</td></tr>
<tr><td>| Symbols: | Transketolase | chr3:22454004-22456824 FORWARD LENGTH=741</td><td>AT3G60750.1 (+1)</td><td>80 kDa</td><td>3</td><td>2</td><td>0</td><td>0</td><td>0</td><td>7</td><td>1</td><td>0</td><td>0</td><td>0</td></tr>
<tr><td>| Symbols: | binding | chr5:19317899-19327014 FORWARD LENGTH=1606</td><td>AT5G47690.2 (+1)</td><td>181 kDa</td><td>6</td><td>0</td><td>0</td><td>2</td><td>0</td><td>6</td><td>0</td><td>0</td><td>0</td><td>0</td></tr>
<tr><td>| Symbols: SAC52, RPL10, RPL10A | Ribosomal protein L16p/L10e family protein | chr1:4888270-4889408 FORWARD LENGTH=220</td><td>AT1G14320.1</td><td>25 kDa</td><td>1</td><td>2</td><td>1</td><td>1</td><td>0</td><td>1</td><td>2</td><td>2</td><td>1</td><td>1</td></tr>
<tr><td>| Symbols: CAC3 | acetyl Co-enzyme a carboxylase carboxyltransferase alpha subunit | chr2:15917612-15920749 FORWARD LENGTH=769</td><td>AT2G38040.1 (+1)</td><td>85 kDa</td><td>3</td><td>1</td><td>0</td><td>0</td><td>0</td><td>4</td><td>0</td><td>0</td><td>0</td><td>0</td></tr>
<tr><td>| Symbols: RAN-1, RAN1, ATRAN1 | RAS-related nuclear protein-1 | chr5:6760364-6761747 FORWARD LENGTH=221</td><td>AT5G20010.1 (+2)</td><td>25 kDa</td><td>2</td><td>3</td><td>0</td><td>2</td><td>0</td><td>5</td><td>1</td><td>0</td><td>0</td><td>0</td></tr>
<tr><td>| Symbols: | Ribosomal protein S4 | chr5:4935124-4936334 REVERSE LENGTH=198</td><td>AT5G15200.1</td><td>23 kDa</td><td>2</td><td>2</td><td>1</td><td>2</td><td>1</td><td>2</td><td>1</td><td>0</td><td>0</td><td>0</td></tr>
<tr><td>| Symbols: | Ribosomal protein S26e family protein | chr2:16918506-16919623 FORWARD LENGTH=133</td><td>AT2G40510.1 (+2)</td><td>15 kDa</td><td>2</td><td>2</td><td>0</td><td>2</td><td>0</td><td>2</td><td>2</td><td>2</td><td>1</td><td>0</td></tr>
<tr><td>| Symbols: EMB2296 | Ribosomal protein L2 family | chr2:7837151-7838160 FORWARD LENGTH=258</td><td>AT2G18020.1</td><td>28 kDa</td><td>1</td><td>1</td><td>1</td><td>0</td><td>1</td><td>1</td><td>1</td><td>1</td><td>1</td><td>0</td></tr>
<tr><td>| Symbols: | Ribosomal protein L2 family | chr4:17097613-17098656 FORWARD LENGTH=258</td><td>AT4G36130.1</td><td>28 kDa</td><td>1</td><td>1</td><td>0</td><td>0</td><td>0</td><td>0</td><td>0</td><td>1</td><td>0</td><td>0</td></tr>
<tr><td>| Symbols: | Histone superfamily protein | chr1:2369212-2369523 FORWARD LENGTH=103</td><td>AT1G07660.1 (+8)</td><td>11 kDa</td><td>1</td><td>1</td><td>1</td><td>3</td><td>1</td><td>3</td><td>1</td><td>1</td><td>1</td><td>1</td></tr>
<tr><td>| Symbols: | oxidoreductases, acting on the aldehyde or oxo group of donors, NAD or NADP as acceptor;copper ion binding | chr2:8613168-8615649 FORWARD LENGTH=401</td><td>AT2G19940.1 (+1)</td><td>44 kDa</td><td>5</td><td>1</td><td>0</td><td>1</td><td>0</td><td>3</td><td>1</td><td>0</td><td>0</td><td>0</td></tr>
<tr><td>| Symbols: ATRPS5B, RPS5B | ribosomal protein 5B | chr2:15647883-15649042 REVERSE LENGTH=207</td><td>AT2G37270.1 (+3)</td><td>23 kDa</td><td>3</td><td>1</td><td>0</td><td>0</td><td>0</td><td>0</td><td>2</td><td>2</td><td>2</td><td>1</td></tr>
<tr><td>| Symbols: | Thioredoxin superfamily protein | chr3:3672189-3673937 FORWARD LENGTH=266</td><td>AT3G11630.1</td><td>29 kDa</td><td>0</td><td>1</td><td>0</td><td>0</td><td>0</td><td>2</td><td>1</td><td>2</td><td>0</td><td>0</td></tr>
<tr><td>| Symbols: PIP1B, TMP-A, ATHH2, PIP1;2 | plasma membrane intrinsic protein 1B | chr2:18910450-18911703 FORWARD LENGTH=286</td><td>AT2G45960.1 (+2)</td><td>31 kDa</td><td>1</td><td>1</td><td>0</td><td>2</td><td>1</td><td>2</td><td>1</td><td>1</td><td>0</td><td>0</td></tr>
<tr><td>| Symbols: RPL27A, RPL27AB | Ribosomal protein L18e/L15 superfamily protein | chr1:8263007-8263447 FORWARD LENGTH=146</td><td>AT1G23290.1 (+1)</td><td>16 kDa</td><td>1</td><td>1</td><td>0</td><td>1</td><td>0</td><td>1</td><td>2</td><td>1</td><td>1</td><td>0</td></tr>
<tr><td>| Symbols: | Ribosomal protein L18ae/LX family protein | chr2:14532916-14534161 REVERSE LENGTH=178</td><td>AT2G34480.1</td><td>21 kDa</td><td>1</td><td>0</td><td>0</td><td>1</td><td>0</td><td>0</td><td>0</td><td>0</td><td>0</td><td>0</td></tr>
<tr><td>| Symbols: | Ribosomal protein L18ae/LX family protein | chr3:4910773-4911933 FORWARD LENGTH=178</td><td>AT3G14600.1</td><td>21 kDa</td><td>0</td><td>0</td><td>0</td><td>0</td><td>0</td><td>0</td><td>0</td><td>0</td><td>0</td><td>0</td></tr>
<tr><td>| Symbols: EMB2719, HAP15 | PAM domain (PCI/PINT associated module) protein | chr1:7001409-7004154 REVERSE LENGTH=488</td><td>AT1G20200.1</td><td>56 kDa</td><td>0</td><td>1</td><td>0</td><td>0</td><td>0</td><td>0</td><td>0</td><td>0</td><td>0</td><td>0</td></tr>
<tr><td>| Symbols: | PAM domain (PCI/PINT associated module) protein | chr1:28524623-28526718 REVERSE LENGTH=487</td><td>AT1G75990.1</td><td>56 kDa</td><td>0</td><td>0</td><td>0</td><td>0</td><td>0</td><td>0</td><td>0</td><td>0</td><td>0</td><td>0</td></tr>
<tr><td>| Symbols: APS1 | ATP sulfurylase 1 | chr3:8112837-8114734 FORWARD LENGTH=463</td><td>AT3G22890.1</td><td>51 kDa</td><td>2</td><td>0</td><td>0</td><td>2</td><td>0</td><td>2</td><td>0</td><td>0</td><td>0</td><td>0</td></tr>
<tr><td>| Symbols: APS2, ASA1 | Pseudouridine synthase/archaeosine transglycosylase-like family protein | chr1:6914835-6916657 REVERSE LENGTH=476</td><td>AT1G19920.1</td><td>54 kDa</td><td>0</td><td>0</td><td>0</td><td>0</td><td>0</td><td>0</td><td>0</td><td>0</td><td>0</td><td>0</td></tr>
<tr><td>| Symbols: APS3 | Pseudouridine synthase/archaeosine transglycosylase-like family protein | chr4:8413443-8415311 REVERSE LENGTH=465</td><td>AT4G14680.1</td><td>52 kDa</td><td>0</td><td>0</td><td>0</td><td>0</td><td>0</td><td>0</td><td>0</td><td>0</td><td>0</td><td>0</td></tr>
<tr><td>| Symbols: | Tetratricopeptide repeat (TPR)-like superfamily protein | chr3:22565440-22566678 REVERSE LENGTH=412</td><td>AT3G60980.1</td><td>47 kDa</td><td>0</td><td>0</td><td>0</td><td>0</td><td>0</td><td>0</td><td>5</td><td>5</td><td>0</td><td>0</td></tr>
<tr><td>| Symbols: SPA2 | SPA1-related 2 | chr4:6772163-6776675 FORWARD LENGTH=1036</td><td>AT4G11110.1</td><td>115 kDa</td><td>5</td><td>6</td><td>0</td><td>0</td><td>0</td><td>0</td><td>0</td><td>0</td><td>0</td><td>0</td></tr>
<tr><td>| Symbols: PORB | protochlorophyllide oxidoreductase B | chr4:13725648-13727107 FORWARD LENGTH=401</td><td>AT4G27440.1 (+1)</td><td>43 kDa</td><td>3</td><td>2</td><td>0</td><td>1</td><td>0</td><td>1</td><td>1</td><td>0</td><td>0</td><td>0</td></tr>
<tr><td>| Symbols: ATPE | ATP synthase epsilon chain | chrC:52265-52663 REVERSE LENGTH=132</td><td>ATCG00470.1</td><td>14 kDa</td><td>2</td><td>2</td><td>0</td><td>1</td><td>0</td><td>1</td><td>1</td><td>2</td><td>0</td><td>0</td></tr>
<tr><td>| Symbols: RPL14 | ribosomal protein L14 | chrC:80696-81064 REVERSE LENGTH=122</td><td>ATCG00780.1</td><td>14 kDa</td><td>2</td><td>2</td><td>0</td><td>1</td><td>0</td><td>2</td><td>1</td><td>2</td><td>0</td><td>0</td></tr>
<tr><td>| Symbols: | C2 calcium/lipid-binding plant phosphoribosyltransferase family protein | chr5:2127200-2129584 REVERSE LENGTH=794</td><td>AT5G06850.1</td><td>91 kDa</td><td>0</td><td>0</td><td>0</td><td>2</td><td>0</td><td>5</td><td>0</td><td>0</td><td>0</td><td>0</td></tr>
<tr><td>| Symbols: FDH | formate dehydrogenase | chr5:4777043-4779190 FORWARD LENGTH=384</td><td>AT5G14780.1</td><td>42 kDa</td><td>2</td><td>1</td><td>0</td><td>1</td><td>0</td><td>3</td><td>0</td><td>1</td><td>0</td><td>0</td></tr>
<tr><td>| Symbols: TOC1, APRR1, PRR1, AtTOC1 | CCT motif -containing response regulator protein | chr5:24675540-24678176 FORWARD LENGTH=618</td><td>AT5G61380.1</td><td>69 kDa</td><td>2</td><td>2</td><td>2</td><td>0</td><td>3</td><td>0</td><td>1</td><td>0</td><td>1</td><td>1</td></tr>
<tr><td>| Symbols: ELF4-L2 | ELF4-like 2 | chr1:27344988-27345347 FORWARD LENGTH=119</td><td>AT1G72630.1</td><td>13 kDa</td><td>0</td><td>1</td><td>0</td><td>1</td><td>0</td><td>0</td><td>1</td><td>1</td><td>1</td><td>1</td></tr>
</table>

| Symbols: STV1, RPL24B, RPL24 | Ribosomal protein L24e family protein | chr3:19660749-19661912 REVERSE LENGTH=163	AT3G53020.1	19 kDa	2	2	0	1	0	1	2	2	0	0
| Symbols: | glycine-rich protein | chr4:11944050-11945565 REVERSE LENGTH=356	AT4G22740.1 (+1)	39 kDa	2	2	0	0	0	2	1	0	0	0
| Symbols: | Ribosomal L28e protein family | chr2:8511752-8512995 FORWARD LENGTH=143	AT2G19730.1 (+2)	16 kDa	1	1	0	0	0	2	0	0	2	0
| Symbols: | Ribosomal protein S11 family protein | chr2:15169925-15171159 FORWARD LENGTH=150	AT2G36160.1	16 kDa	1	1	0	1	0	1	0	0	0	0
| Symbols: | Ribosomal protein S11 family protein | chr3:3623757-3624866 REVERSE LENGTH=150	AT3G11510.1	16 kDa	0	1	0	0	0	0	0	0	0	0
| Symbols: | Coatomer, beta' subunit | chr3:5411699-5418313 REVERSE LENGTH=909	AT3G15980.1 (+3)	102 kDa	1	1	0	0	0	2	0	0	0	0
| Symbols: | structural molecules | chr1:30084522-30091949 FORWARD LENGTH=1135	AT1G79990.1 (+2)	129 kDa	0	0	0	0	0	0	0	0	0	0
| Symbols: XIK, ATXIK, XI-17 | Myosin family protein with Dil domain | chr5:6927064-6936825 REVERSE LENGTH=1545	AT5G20490.1 (+1)	175 kDa	1	1	0	0	0	0	0	0	0	0
| Symbols: MYA1, ATMYA1, XI-1 | myosin 1 | chr1:6039453-6049309 FORWARD LENGTH=1520	AT1G17580.1	173 kDa	1	0	0	0	0	0	0	0	0	0
| Symbols: XIC, ATXIC | Myosin family protein with Dil domain | chr1:2779963-2788325 FORWARD LENGTH=1538	AT1G08730.1	175 kDa	0	0	0	0	0	0	0	0	0	0
| Symbols: HSP81-2, ERD8, HSP90.2, AtHsp90.2 | heat shock protein 81-2 | chr5:22686923-22689433 FORWARD LENGTH=699	AT5G56030.1 (+1)	80 kDa	3	2	0	1	0	1	1	0	0	0
| Symbols: PIP3, PIP3A, PIP2;7, SIMIP | plasma membrane intrinsic protein 3 | chr4:16708672-16709958 FORWARD LENGTH=280	AT4G35100.1 (+1)	30 kDa	2	2	1	1	1	0	1	0	0	0
| Symbols: RPS4 | chloroplast ribosomal protein S4 | chrC:45223-45828 REVERSE LENGTH=201	ATCG00380.1	23 kDa	1	1	0	0	0	0	2	2	0	0
| Symbols: LOS2, ENO2 | Enolase | chr2:15321081-15323786 REVERSE LENGTH=444	AT2G36530.1	48 kDa	1	1	0	1	0	2	1	0	1	0
| Symbols: | ATPase, V0/A0 complex, subunit C/D | chr3:10778025-10780350 FORWARD LENGTH=351	AT3G28715.1	41 kDa	2	1	0	0	0	0	1	0	0	0
| Symbols: | ATPase, V0/A0 complex, subunit C/D | chr3:10773144-10775594 REVERSE LENGTH=351	AT3G28710.1	41 kDa	1	1	0	1	0	1	0	0	0	0
| Symbols: cpHsc70-1 | chloroplast heat shock protein 70-1 | chr4:12590094-12593437 FORWARD LENGTH=718	AT4G24280.1	77 kDa	0	0	0	0	0	1	0	0	0	0
| Symbols: CPHSC70-2EAT SHOCK PROTEIN 70-2, HSC70-7, cpHsc70-2 | chloroplast heat shock protein 70-2 | chr5:20303470-20306295 FORWARD LENGTH=718	AT5G49910.1	77 kDa	0	0	0	1	0	0	0	0	0	0
| Symbols: LUH | LEUNIG_homolog | chr2:13867235-13871844 FORWARD LENGTH=787	AT2G32700.1 (+6)	86 kDa	0	0	0	1	0	3	0	0	0	0
| Symbols: emb1138 | DEAD box RNA helicase (RH3) | chr5:9285540-9288871 REVERSE LENGTH=747	AT5G26742.1 (+1)	81 kDa	3	2	0	1	0	0	0	0	0	0
| Symbols: RPS11 | ribosomal protein S11 | chrC:78960-79376 REVERSE LENGTH=138	ATCG00750.1	15 kDa	2	2	0	1	0	1	2	2	0	0
| Symbols: | Ribosomal protein S4 (RPS4A) family protein | chr2:7546598-7548138 FORWARD LENGTH=261	AT2G17360.1 (+3)	30 kDa	2	0	1	1	0	1	1	0	0	0
| Symbols: PFL, RPS18A, PFL1 | Ribosomal protein S13/S18 family | chr1:8067990-8069163 FORWARD LENGTH=152	AT1G22780.1 (+2)	18 kDa	0	1	1	2	0	1	0	1	1	0
| Symbols: P40, AP40, RP40, RPSAA | 40s ribosomal protein SA | chr1:27243148-27244842 REVERSE LENGTH=298	AT1G72370.1 (+1)	32 kDa	1	2	0	1	0	1	0	0	0	0
| Symbols: RPSAb | 40s ribosomal protein SA B | chr3:1309544-1310846 REVERSE LENGTH=280	AT3G04770.2	31 kDa	0	0	0	0	0	0	0	0	0	0
| Symbols: CLPC, ATHSP93-V, HSP93-V, DCA1, CLPC1 | CLPC homologue 1 | chr5:20715710-20719800 REVERSE LENGTH=929	AT5G50920.1	?	1	0	0	0	0	0	0	0	0	0
| Symbols: ATCLPC, ATHSP93-III, HSP93-III | Clp ATPase | chr3:18122363-18126008 REVERSE LENGTH=952	AT3G48870.1 (+1)	106 kDa	0	0	0	0	0	0	0	0	0	0
| Symbols: SPA4 | SPA1-related 4 | chr1:19783748-19786690 FORWARD LENGTH=794	AT1G53090.1 (+1)	89 kDa	2	5	0	0	0	0	0	0	0	0
| Symbols: | AAA-type ATPase family protein | chr2:8692736-8694837 FORWARD LENGTH=443	AT2G20140.1 (+1)	49 kDa	5	3	0	0	0	0	1	0	0	0
| Symbols: RPT6A, ATSUG1 | regulatory particle triple-A ATPase 6A | chr5:6752144-6754918 FORWARD LENGTH=419	AT5G19990.1 (+1)	47 kDa	0	0	0	1	0	2	0	0	0	0
| Symbols: | Lactate/malate dehydrogenase family protein | chr1:1189418-1191267 REVERSE LENGTH=332	AT1G04410.1	36 kDa	0	2	0	1	0	1	0	0	0	0
| Symbols: | Aldolase superfamily protein | chr3:19627383-19628874 REVERSE LENGTH=358	AT3G52930.1	39 kDa	0	1	0	0	0	1	0	0	1	0
| Symbols: | Aldolase superfamily protein | chr5:963389-964982 REVERSE LENGTH=359	AT5G03690.2	39 kDa	0	0	0	0	0	0	0	0	0	0
| Symbols: | Aldolase superfamily protein | chr4:13391566-13392937 FORWARD LENGTH=358	AT4G26530.1 (+1)	38 kDa	0	0	0	0	0	0	0	0	0	0
| Symbols: CHLI1, CH42, CH-42, CHL11, CHLI-1 | P-loop containing nucleoside triphosphate hydrolases superfamily protein | chr4:10201897-10203361 REVERSE LENGTH=424	AT4G18480.1	46 kDa	1	0	0	0	0	1	0	0	0	0
| Symbols: CHLI2, CHL I2, CHLI-2 | magnesium chelatase i2 | chr5:18628095-18629565 FORWARD LENGTH=418	AT5G45930.1	46 kDa	0	0	0	0	0	0	0	0	0	0
| Symbols: | transducin family protein / WD-40 repeat family protein | chr3:23431009-23437241 REVERSE LENGTH=1104	AT3G63460.1 (+2)	120 kDa	1	1	0	1	0	2	0	0	0	0
| Symbols: | vacuolar ATP synthase subunit H family protein | chr3:14228846-14232228 REVERSE LENGTH=441	AT3G42050.1	50 kDa	2	1	0	1	0	1	0	0	0	0
| Symbols: RPL18 | ribosomal protein L18 | chr3:1621511-1622775 FORWARD LENGTH=187	AT3G05590.1 (+1)	21 kDa	2	1	1	1	0	2	1	1	0	0
| Symbols: RPT5A, ATS6A.2 | regulatory particle triple-A ATPase 5A | chr3:1603540-1605993 FORWARD LENGTH=424	AT3G05530.1	47 kDa	2	1	0	0	0	1	1	1	0	0
| Symbols: AGT, AGT1, SGAT | alanine:glyoxylate aminotransferase | chr2:5539417-5540902 REVERSE LENGTH=401	AT2G13360.1 (+1)	44 kDa	1	1	1	0	1	2	0	0	0	0
| Symbols: | Ribosomal protein L22p/L17e family protein | chr1:9515230-9516725 FORWARD LENGTH=176	AT1G27400.1 (+1)	20 kDa	0	1	2	0	1	2	0	0	0	0
| Symbols: VHA-A | vacuolar ATP synthase subunit A | chr1:29660463-29664575 FORWARD LENGTH=623	AT1G78900.1 (+1)	69 kDa	1	0	0	0	0	2	0	0	0	0
| Symbols: RPT1A | regulatory particle triple-A 1A | chr1:20065921-20068324 REVERSE LENGTH=426	AT1G53750.1	48 kDa	2	1	0	1	0	1	0	0	0	0
| Symbols: ATJ2, J2 | DNAJ homologue 2 | chr5:7303798-7305668 REVERSE LENGTH=419	AT5G22060.1	46 kDa	0	0	0	0	0	0	0	0	0	0
| Symbols: ATJ3, ATJ | DNAJ homologue 3 | chr3:15869115-15871059 REVERSE LENGTH=420	AT3G44110.1 (+1)	46 kDa	0	0	0	0	0	1	0	0	0	0
| Symbols: RPS1, ARRPS1 | ribosomal protein S1 | chr5:11619262-11621223 REVERSE LENGTH=416	AT5G30510.1	45 kDa	1	0	0	3	0	4	0	0	0	0
| Symbols: ACCD | acetyl-CoA carboxylase carboxyl transferase subunit beta | chrC:57075-58541 FORWARD LENGTH=488	ATCG00500.1	56 kDa	2	1	0	0	0	1	0	0	0	0
| Symbols: RPS8 | ribosomal protein S8 | chrC:80068-80472 REVERSE LENGTH=134	ATCG00770.1	15 kDa	1	2	0	1	0	1	1	1	0	0
| Symbols: SSR16 | small subunit ribosomal protein 16 | chr4:16535084-16536092 REVERSE LENGTH=113	AT4G34620.1	13 kDa	1	1	0	1	0	1	1	1	2	0
| Symbols: | Sucrose-6F-phosphate phosphohydrolase family protein | chr2:15053952-15055776 FORWARD LENGTH=422	AT2G35840.1 (+2)	48 kDa	1	0	0	0	0	2	0	0	0	0
| Symbols: | GTP binding Elongation factor Tu family protein | chr4:1295751-1298354 REVERSE LENGTH=454	AT4G02930.1	49 kDa	3	0	0	1	0	3	0	0	0	0
| Symbols: ECT2 | evolutionarily conserved C-terminal region 2 | chr3:4385274-4388220 REVERSE LENGTH=667	AT3G13460.1 (+3)	72 kDa	3	2	0	0	0	0	0	0	0	0
| Symbols: | Glycine cleavage T-protein family | chr1:4001801-4003245 FORWARD LENGTH=408	AT1G11860.1 (+2)	44 kDa	1	1	0	1	0	1	2	0	0	0
| Symbols: NOP56 | homolog of nucleolar protein NOP56 | chr1:20984544-20986893 REVERSE LENGTH=522	AT1G56110.1	59 kDa	1	0	0	0	0	2	0	0	0	0
| Symbols: RPS15A | ribosomal protein S15A | chr1:2408413-2409065 REVERSE LENGTH=130	AT1G07770.1 (+2)	15 kDa	1	1	1	0	1	3	0	0	0	0
| Symbols: | Ribosomal protein S8 family protein | chr2:16517588-16518265 REVERSE LENGTH=136	AT2G39590.1	15 kDa	0	0	0	0	0	0	0	0	0	0
| Symbols: RPN1A, ATRPN1A | 26S proteasome regulatory subunit S2 1A | chr2:8859211-8864699 FORWARD LENGTH=891	AT2G20580.1	?	2	1	0	0	0	0	0	0	0	0
| Symbols: PAC1 | 20S proteasome alpha subunit C1 | chr3:7792819-7793571 REVERSE LENGTH=250	AT3G22110.1	27 kDa	0	2	0	0	0	1	1	1	0	0
| Symbols: ATBBC1, BBC1, RSU2 | breast basic conserved 1 | chr3:18166971-18168047 REVERSE LENGTH=206	AT3G49010.1 (+3)	24 kDa	0	0	0	1	0	2	1	0	0	0
| Symbols: | Tetratricopeptide repeat (TPR)-like superfamily protein | chr3:22535729-22536940 FORWARD LENGTH=403	AT3G60960.1	46 kDa	0	0	0	0	0	0	1	2	0	0
| Symbols: CPN60A, CH-CPN60A, SLP | chaperonin-60alpha | chr2:11926603-11929184 FORWARD LENGTH=586	AT2G28000.1	62 kDa	2	1	0	0	0	0	0	0	0	0
| Symbols: ATTIP49A, RIN1 | P-loop containing nucleoside triphosphate hydrolases superfamily protein | chr5:7391026-7394071 REVERSE LENGTH=458	AT5G22330.1	50 kDa	0	2	0	1	0	1	0	0	0	0
| Symbols: RPL16 | ribosomal protein L16 | chrC:81189-82652 REVERSE LENGTH=135	ATCG00790.1	15 kDa	0	0	0	0	0	1	0	0	0	0
| Symbols: PUB12, AtPUB12 | PLANT U-BOX 12 | chr2:12367001-12370608 REVERSE LENGTH=962	AT2G28830.1	107 kDa	0	0	0	0	0	0	0	0	0	0
| Symbols: | NAD(P)-binding Rossmann-fold superfamily protein | chr3:11193767-11194948 REVERSE LENGTH=298	AT3G29250.1	32 kDa	0	0	0	1	0	3	0	0	0	0
| Symbols: ILA | ILITYHIA | chr1:24065232-24081908 REVERSE LENGTH=2610	AT1G64790.1 (+1)	285 kDa	3	0	0	0	0	0	0	0	0	0
| Symbols: APUM5, PUM5 | pumilio 5 | chr3:7059098-7062660 REVERSE LENGTH=961	AT3G20250.1	107 kDa	2	0	0	1	0	0	0	0	0	0
| Symbols: ATTPS7, TPS7, ATTPSA | trehalose-phosphatase/synthase 7 | chr1:1955413-1958153 FORWARD LENGTH=851	AT1G06410.1	97 kDa	1	0	0	0	0	3	0	0	0	0
| Symbols: | transcription regulators | chr1:373335-386682 FORWARD LENGTH=2431	AT1G02080.1 (+1)	270 kDa	2	1	0	0	0	0	0	0	0	0
| Symbols: ATGSTF9, GLUTTR, ATGSTF7, GSTF9 | glutathione S-transferase PHI 9 | chr2:13139132-13140057 FORWARD LENGTH=215	AT2G30860.1 (+1)	24 kDa	0	2	0	1	0	0	0	0	0	0
| Symbols: | Ribosomal protein L35Ae family protein | chr1:27928415-27929466 REVERSE LENGTH=112	AT1G74270.1	13 kDa	1	0	0	1	0	1	0	0	0	0
| Symbols: ATU2AF35B, U2AF35B | Zinc finger C-x8-C-x5-C-x3-H type family protein | chr5:17170445-17171296 REVERSE LENGTH=283	AT5G42820.1 (+1)	33 kDa	1	0	0	0	0	0	0	0	0	0
| Symbols: ATU2AF35A | U2 snRNP auxiliary factor small subunit, putative | chr1:9615302-9616042 FORWARD LENGTH=246	AT1G27650.2	29 kDa	0	0	0	0	0	0	0	0	0	0
| Symbols: | Zinc finger C-x8-C-x5-C-x3-H type family protein | chr3:16328221-16328448 REVERSE LENGTH=75	AT3G44785.1	9 kDa	0	0	0	0	0	0	0	0	0	0
| Symbols: PIL6, PIF5 | phytochrome interacting factor 3-like 6 | chr3:21828189-21829895 REVERSE LENGTH=442	AT3G59060.1 (+3)	49 kDa	0	0	0	0	0	0	1	0	0	0
| Symbols: PIF4, SRL2, AtPIF4 | phytochrome interacting factor 4 | chr2:17887003-17888823 FORWARD LENGTH=430	AT2G43010.1 (+1)	48 kDa	0	0	0	0	0	0	0	0	0	0
| Symbols: | Ribosomal protein L31e family protein | chr2:8513577-8514346 FORWARD LENGTH=119	AT2G19740.1 (+2)	14 kDa	0	0	0	1	0	1	0	0	0	0
| Symbols: | Ribosomal protein L31e family protein | chr5:22944003-22944767 REVERSE LENGTH=85	AT5G56710.2	10 kDa	0	0	0	0	0	0	0	0	0	0
| Symbols: | Coatomer epsilon subunit | chr1:10858546-10860173 REVERSE LENGTH=292	AT1G30630.1	33 kDa	0	0	0	2	0	2	0	0	0	0
| Symbols: SHM4 | serine hydroxymethyltransferase 4 | chr4:8048013-8050021 REVERSE LENGTH=471	AT4G13930.1	52 kDa	2	1	0	0	0	0	0	0	0	0
| Symbols: RPL22 | ribosomal protein L22 | chrC:83467-83949 REVERSE LENGTH=160	ATCG00810.1	19 kDa	0	0	0	1	0	2	0	0	0	0
| Symbols: PBE1 | 20S proteasome beta subunit E1 | chr1:4452641-4454663 FORWARD LENGTH=274	AT1G13060.1 (+2)	30 kDa	0	2	0	0	0	0	0	0	0	0
| Symbols: PSBB | photosystem II reaction center protein B | chrC:72371-73897 FORWARD LENGTH=508	ATCG00680.1	56 kDa	1	1	0	0	0	0	2	0	0	0
| Symbols: NRPB1, RPB1, RNA_POL_II_LSRNA_POL_II_LS, RNA_POL_II_LS | RNA polymerase II large subunit | chr4:16961115-16967892 REVERSE LENGTH=1839	AT4G35800.1	205 kDa	2	0	0	0	0	0	0	0	0	0

<table>
<tr><td>| Symbols: EMB2761 | threonyl-tRNA synthetase, putative / threonine--tRNA ligase, putative | chr2:1698466-1701271 REVERSE LENGTH=650</td><td>AT2G04842.1</td><td>75 kDa</td><td>0</td><td>0</td><td>0</td><td>0</td><td>0</td><td>2</td><td>0</td><td>0</td><td>0</td><td>0</td></tr>
<tr><td>| Symbols: ELF4-L4 | ELF4-like 4 | chr1:5997932-5998276 FORWARD LENGTH=114</td><td>AT1G17455.1 (+1)</td><td>13 kDa</td><td>0</td><td>0</td><td>0</td><td>0</td><td>0</td><td>0</td><td>1</td><td>1</td><td>0</td><td>0</td></tr>
<tr><td>| Symbols: | AAA-type ATPase family protein | chr1:17009220-17011607 FORWARD LENGTH=399</td><td>AT1G45000.1 (+2)</td><td>45 kDa</td><td>1</td><td>1</td><td>0</td><td>0</td><td>0</td><td>0</td><td>1</td><td>0</td><td>0</td><td>0</td></tr>
<tr><td>| Symbols: PHOT1, NPH1, JK224, RPT1 | phototropin 1 | chr3:16818557-16823960 FORWARD LENGTH=996</td><td>AT3G45780.1 (+1)</td><td>112 kDa</td><td>2</td><td>0</td><td>0</td><td>0</td><td>0</td><td>1</td><td>0</td><td>0</td><td>0</td><td>0</td></tr>
<tr><td>| Symbols: PHOT2, NPL1 | phototropin 2 | chr5:23524771-23529993 FORWARD LENGTH=915</td><td>AT5G58140.1 (+3)</td><td>102 kDa</td><td>0</td><td>0</td><td>0</td><td>0</td><td>0</td><td>0</td><td>0</td><td>0</td><td>0</td><td>0</td></tr>
<tr><td>| Symbols: ZFN1 | zinc finger protein 1 | chr3:614075-615916 FORWARD LENGTH=397</td><td>AT3G02830.1</td><td>44 kDa</td><td>2</td><td>0</td><td>0</td><td>0</td><td>0</td><td>0</td><td>0</td><td>0</td><td>0</td><td>0</td></tr>
<tr><td>| Symbols: | Ribosomal protein L35Ae family protein | chr1:15651585-15652427 REVERSE LENGTH=111</td><td>AT1G41880.1</td><td>13 kDa</td><td>0</td><td>0</td><td>0</td><td>1</td><td>0</td><td>1</td><td>0</td><td>0</td><td>0</td><td>0</td></tr>
<tr><td>| Symbols: SPA3 | SPA1-related 3 | chr3:5169327-5172480 REVERSE LENGTH=837</td><td>AT3G15354.1</td><td>93 kDa</td><td>0</td><td>1</td><td>0</td><td>0</td><td>0</td><td>0</td><td>0</td><td>0</td><td>0</td><td>0</td></tr>
<tr><td>| Symbols: PRP39 | Tetratricopeptide repeat (TPR)-like superfamily protein | chr1:1051803-1056550 FORWARD LENGTH=768</td><td>AT1G04080.1 (+1)</td><td>85 kDa</td><td>2</td><td>0</td><td>0</td><td>0</td><td>0</td><td>0</td><td>0</td><td>0</td><td>0</td><td>0</td></tr>
<tr><td>| Symbols: ABA1, LOS6, NPQ2, ATABA1, ZEP, IBS3, ATZEP | zeaxanthin epoxidase (ZEP) (ABA1) | chr5:26753745-26757090 REVERSE LENGTH=667</td><td>AT5G67030.1 (+1)</td><td>74 kDa</td><td>2</td><td>0</td><td>0</td><td>0</td><td>0</td><td>0</td><td>0</td><td>0</td><td>0</td><td>0</td></tr>
</table>
