# Supplementary material for: Cross‐species complementation reveals conserved functions for EARLY FLOWERING 3 between monocots and dicots
Source: Plant Direct. 2017 Oct 16;1(4):e00018. doi: 10.1002/pld3.18 (PMC6508535; doi:10.1002/pld3.18)
Supplement: Supplementary file 2 [file PLD3-1-e00018-s002.pdf]

Reviewer Comments:
Reviewer #1:
Comments to the Author (Required):
This revised version of the manuscript addresses all the issues raised in my review.

Reviewer #2:
Comments to the Author (Required):
This manuscript "Cross-Species Complementation Reveals Conserved Functions For EARLY FLOWERING 3 Between Monocots And Dicots" addresses potential conservation of EARLY FLOWERING 3 (ELF3) protein across diverse species, from Arabidopsis thaliana to Brachopodium distachyon and Setaria viridis.
The authors use trascriptomics to characterise the diurnal regulation in these species, as well as functional testing in light signalling and the circadian clock by introduction of Brachopodium distachyon and Setaria viridis ELF3 in elf3 mutant Arabidopsis thaliana. Yeast 2 hybrid assays are further used to show the potential of putative ELF3 Brachopodium distachyon and Setaria viridis proteins to directly interact with Arabidopsis thaliana Evening Complex proteins. Further they address protein interactions of Brachopodium distachyon and Setaria viridis ELF3 following their expression in planta in Arabidopsis thaliana elf3 mutants. Together these results show the competence of Brachopodium distachyon and Setaria viridis proteins to directly interact with circadian and/or light signalling proteins in planta and substitute for their Arabidopsis thaliana counterparts, supporting the notion that the function of dicot and monocot ELF3 protein show certain biochemical and functional conservation.
In general the work is interesting and well done. I suggest that the authors add an additional (new) reference on fitness i.e. Rubin, M. J., M. T. Brock, A. M. Davis, Z. M. German, M. Knapp, S. M. Welch, S. L. Harmer, J. N. Maloof, S. J. Davis and C. Weinig (2017) Circadian rhythms vary over the growing season and correlate with fitness components. Molecular Ecology on-line, DOI: 10.1111/mec.14287.
